# Glabridin‐Gold(I) Complex as a Novel Immunomodulatory Agent Targeting TrxR and MAPK Pathways for Synergistic Enhancement of Antitumor Immunity

**DOI:** 10.1002/advs.202504729

**Published:** 2025-08-21

**Authors:** Zhaoran Wang, Meiyu Wang, Qiong Chen, Mengshi Wang, Fuwei Li, Lin Lv, Zhenfan Wen, Zhongren Xu, Yixia Yang, Chunyang Bi, Wukun Liu

**Affiliations:** ^1^ Jiangsu Collaborative Innovation Center of Chinese Medicinal Resources Industrialization School of Medicine Nanjing University of Chinese Medicine Nanjing 210023 P. R. China; ^2^ School of Traditional Chinese Medicine Jiangsu College of Nursing Huai'an 223005 P. R. China; ^3^ Shanghai General Hospital Shanghai Jiaotong University Shanghai 200080 P. R. China

**Keywords:** Immunomodulatory agent, immunosuppressive microenvironment, metal complex, tumor immunotherapy

## Abstract

Metal‐based drugs have been utilized as immunomodulatory agents in combination with cancer immunotherapies to induce tumor immunogenicity. However, the immunosuppressive tumor microenvironment significantly hinders the efficacy of these immunomodulatory agents from promoting antitumor immune responses. Herein, a novel metal‐based immunomodulatory agent, **6d**, is developed by integrating *N*‐heterocyclic carbene gold(I) [NHC‐Au(I)] with the natural product glabridin (GLA). Complex **6d** aims to promote tumor immunogenicity while suppressing immunosuppression, by targeting thioredoxin reductase (TrxR) and the mitogen‐activated protein kinase (MAPK) pathways. Notably, **6d** enhances dendritic cell (DC) maturation while reducing myeloid‐derived suppressor cells (MDSCs), M2‐type macrophages, and regulatory T cells (Tregs) in liver cancer. Moreover, **6d** exhibits a synergistic effect of gold center and GLA, suppressing programmed cell death 1 ligand 1 (PD‐L1) expression in tumor cells while promoting granzyme B (GzmB) production in T cells. These findings suggest that dual inhibition of TrxR and MAPK may provide a synergistic strategy to stimulate antitumor immunity while mitigating the immunosuppressive tumor microenvironment. Overall, this study warrants further researches to determine therapeutic efficacy of **6d** as an immunomodulatory agent in combination with cancer immunotherapies.

## Graphical abstract

Complex **6d** could promote tumor immunogenicity by targeting thioredoxin reductase (TrxR) and remodel immunosuppressive microenvironment by the mitogen‐activated protein kinase (MAPK) pathway inhibition simultaneously. This study provides a novel approach to develop metal immunomodulatory agents by designing gold complexes incorporating natural products, and suggests a possible strategy to promote antitumor immune responses while avoiding immunosuppressive microenvironment activation.

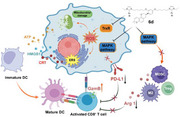

## 1 Introduction

Immunotherapy, including immune checkpoint inhibitors and T cell‐based therapies, has garnered significant attention in tumor treatment by restoring the antitumor immune response.^[^ ^1^ ^]^ However, the tumor microenvironment poses barriers that limit the efficacy of these therapies.^[^ ^2^ ^]^ To overcome these challenges, small‐molecule immunomodulatory agents have been utilized to enhance immunotherapy outcomes by combination strategies.^[^ ^3^ ^]^ Metal complexes and materials can stimulate the antitumor immune response by restoring immune surveillance of cancer cells, alleviating immune suppression in immune cells, and inducing immunogenic cell death (ICD).^[^ ^4^ ^]^ Among these, oxaliplatin (OXA) has been identified as an ICD inducer, capable of enhancing tumor cell immunogenicity and restoring tumor antigen presentation.^[^ ^5^ ^]^ Hepatic arterial infusion chemotherapy employs OXA to release tumor antigens,^[^ ^6^ ^]^ while immune checkpoint inhibitors targeting programmed cell death protein 1 (PD‐1) are combined with OXA to promote antigen presentation and T cell activation.^[^ ^7^ ^]^ Currently, the adverse reactions and resistance associated with platinum‐based complexes are driving the search for alternative metal complexes, including gold complexes.^[^ ^8^ ^]^ Overexpressed thioredoxin reductase (TrxR) in various cancer cells is a promising therapeutic target,^[^ ^9^ ^]^ and gold complexes, exemplified by auranofin (AF), inhibit TrxR to elevate reactive oxygen species (ROS) levels for cancer treatment.^[^ ^10^ ^]^ Additionally, gold complexes can enhance tumor immunogenicity through ROS‐induced endoplasmic reticulum stress (ERS) and subsequent damage‐associated molecular patterns (DAMPs).^[^ ^11^ ^]^

However, the immunity‐related effects of metal complexes do not always contribute to tumor suppression. In some cases, immunotherapy may instead exacerbate the acquired activation of the immunosuppressive microenvironment, leading to T cell dysfunction, antigen loss, increased immune checkpoint expression, and the recruitment of immunosuppressive cells.^[^ ^12^ ^]^ For instance, OXA can activate the tumor immunosuppressive microenvironment in liver cancer by upregulating programmed cell death 1 ligand 1 (PD‐L1) and recruiting myeloid‐derived suppressor cells (MDSCs).^[^ ^13^ ^]^ Similarly, AF has been reported to upregulate PD‐L1 expression, leading to immune evasion by stimulating the Ras‐Raf‐MEK‐ERK mitogen‐activated protein kinase (MAPK) pathway.^[^ ^14^ ^]^ Therefore, enhancing immune response rates by simultaneously remodeling the immunosuppressive microenvironment and improving tumor immunogenicity is considered a promising therapeutic strategy.^[^ ^15^ ^]^

Metal complexes coupled with natural products or their derivatives have gained increasing attention, as this strategy elicits a robust antitumor immune response.^[^ ^16^ ^]^ Natural products have been identified as potential immunomodulatory agents capable of remodeling the tumor immunosuppressive microenvironment.^[^ ^17^ ^]^ Among them, glabridin (GLA), an active ingredient in liquorice, has been extensively studied for its therapeutic potential in liver diseases.^[^ ^18^ ^]^ GLA has been reported to inhibit the phosphorylation of mitogen‐activated protein kinase kinases 1/2 (MEK1/2) and extracellular signal‐regulated kinases 1/2 (ERK1/2) in the MAPK pathway.^[^ ^19^ ^]^ Growing evidence suggests that MAPK pathway overactivation mediates crosstalk between tumors, the tumor microenvironment, and the immune system, contributing to immune escape mechanisms such as the activation of immunosuppressive cells and the upregulation of PD‐L1 expression.^[^ ^20^ ^]^ Thus, pharmacological disruption of this pathway could help restore the antitumor immune microenvironment. Consequently, GLA may regulate the tumor immunosuppressive microenvironment by inhibiting the MAPK pathway. Additionally, GLA exhibits immunomodulatory properties, including the regulation of macrophage polarization and inhibition of arginase‐1 (Arg‐1).^[^ ^21^ ^]^ Arg‐1 plays a critical role in establishing an immunosuppressive microenvironment by suppressing T cell proliferation.^[^ ^22^ ^]^

**Scheme 1:**
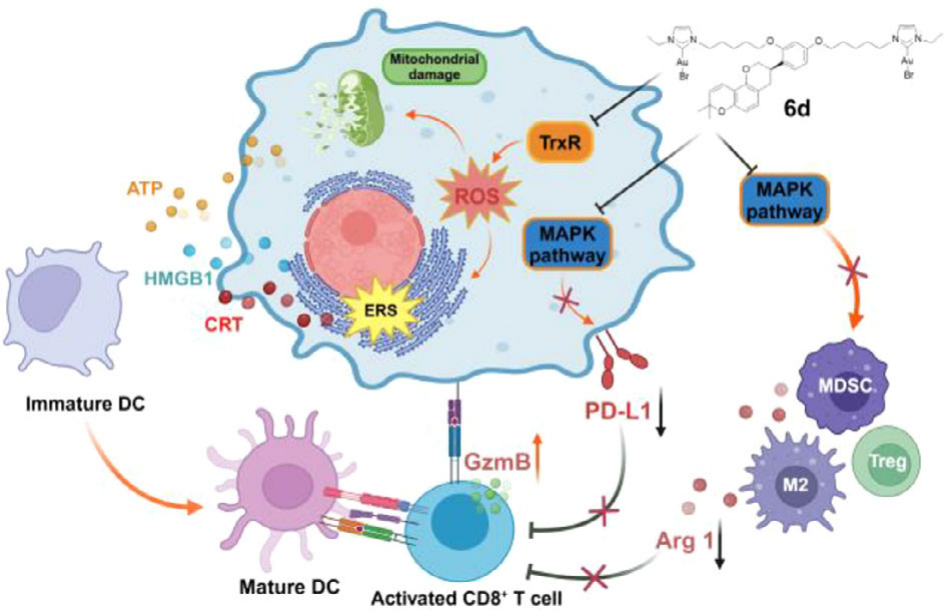
The glabridin‐gold(I) complex **6d** enhances tumor immunogenicity by targeting TrxR while simultaneously remodeling the immunosuppressive microenvironment through MAPK pathway inhibition (Image created with BioRender; M2 is M2‐type macrophage in this image).

In this study, a gold complex, **6d,** containing GLA, was designed to develop a novel immunomodulatory agent targeting both TrxR and MAPK, with the goal of restoring tumor immunogenicity and remodeling the immunosuppressive microenvironment simultaneously. The results demonstrate that **6d** promotes dendritic cell (DC) maturation while suppressing MDSCs, M2‐type macrophages, and regulatory T cells (Tregs). Notably, the synergistic effect of the gold center and GLA downregulates PD‐L1 expression in tumor cells and enhances granzyme B (GzmB) expression in T cells. To the best of our knowledge, this is the first example of a gold complex designed for the simultaneous inhibition of TrxR and MAPK, with the potential to promote antitumor immune responses by inducing tumor immunogenicity and remodeling the immunosuppressive microenvironment. This study provides a novel strategy for developing metal‐based immunomodulatory agents by incorporating natural products into gold complexes (**Scheme** **1**).

**Scheme 2:**
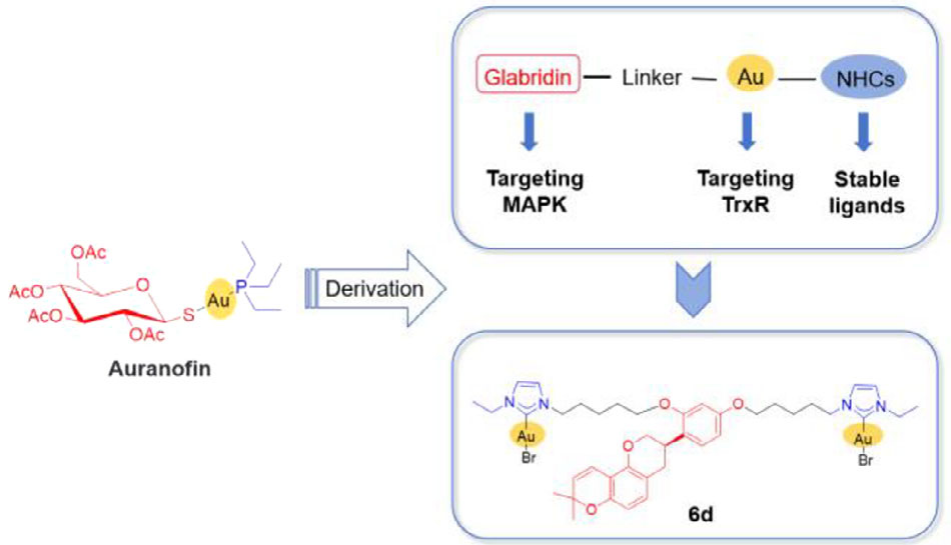
Construction of the glabridin‐gold(I) complex **6d**, designed to target both MAPK and TrxR.

## 2 Results and Discussion

### 2.1 Design, Synthesis, and Characterization

The classical gold‐based drug AF exhibits anticancer activity by inhibiting TrxR.^[^ ^10^ ^]^ Previous structure‐activity relationship (SAR) studies indicate that the phosphine ligand plays a crucial role in its biological potency.^[^ ^23^ ^]^ The comparable donor properties of *N*‐heterocyclic carbenes (NHCs) to phosphines make NHCs attractive alternatives.^[^ ^24^ ^]^ Additionally, the strong σ‐donor and antioxidant properties of NHCs enhance the chemical and thermal stability of gold complexes.^[^ ^25^ ^]^ Moreover, the aurophilicity between two gold atoms in binuclear NHC‐Au(I) complexes can influence the electronic structure of the complexes and improve their stability under physiological conditions. GLA, an active ingredient in liquorice, targets the MAPK pathway. Therefore, we designed and synthesized nine binuclear and two mononuclear NHC‐Au(I) complexes containing GLA with varying alkane chain lengths. Overall, our goal is to develop a novel immunomodulatory agent targeting both TrxR and MAPK through structural modifications (**Scheme** **2**).

To obtain **3a**–**3c,** dibromoalkanes (**2a**–**2c**) with a ‐OH group on GLA formed an ether bond through an etherification reaction in the presence of potassium carbonate. Subsequently, the corresponding NHC ligands, as previously reported (**4a**–**4c**),^[^ ^26^ ^]^ were refluxed with **3a**–**3c** to yield the ligands (**5a**–**5i**). Finally, the binuclear NHC‐Au(I) complexes **6a**–**6i** were synthesized through ligands and gold coordination (Scheme S1, Supporting Information). Additionally, mononuclear NHC‐Au(I) complexes **6j** and **6k** were synthesized as controls using a similar method (Scheme S2, Supporting Information).

All complexes were characterized by ^1^H and ^13^C nuclear magnetic resonance (NMR) spectroscopy and electrospray ionization mass spectrometry (ESI‐MS). The purity of the NHC‐Au(I) complexes was confirmed by high‐performance liquid chromatography (HPLC). Detailed data on the complexes are provided in the Supporting Information (Figure S1–S33, Supporting Information).

### 2.2 Stability Research

First, we tested the stability of the dominant complex **6d** and AF in DMSO‐*d_6_* and DMSO‐*d_6_*/D_2_O (v_1_/v_2_ = 9:1) over 72 h using ^1^H NMR. As expected, both complexes remained stable in these solutions for 72 h (Figure S34–S37, Supporting Information), with no significant changes in their ^1^H NMR spectra. Furthermore, we evaluated the stability of **6d** (5 mM) and AF (5 mM) in DMSO‐*d_6_*/D_2_O (v_1_/v_2_ = 9:1) containing GSH (5 mM). The ^1^H NMR spectra revealed that **6d** began to change after 12 h (Figure S38, Supporting Information), whereas AF showed new peaks after just 2 h (Figure S39, Supporting Information). These results indicate that **6d** exhibits superior stability compared to AF in the presence of GSH, aligning with our design expectations. In addition, complex **6d** also showed a high metabolic stability in whole blood within 2 h (Table S1, Supporting Information).

### 2.3 Cytotoxicity Analysis

**Figure 1:**
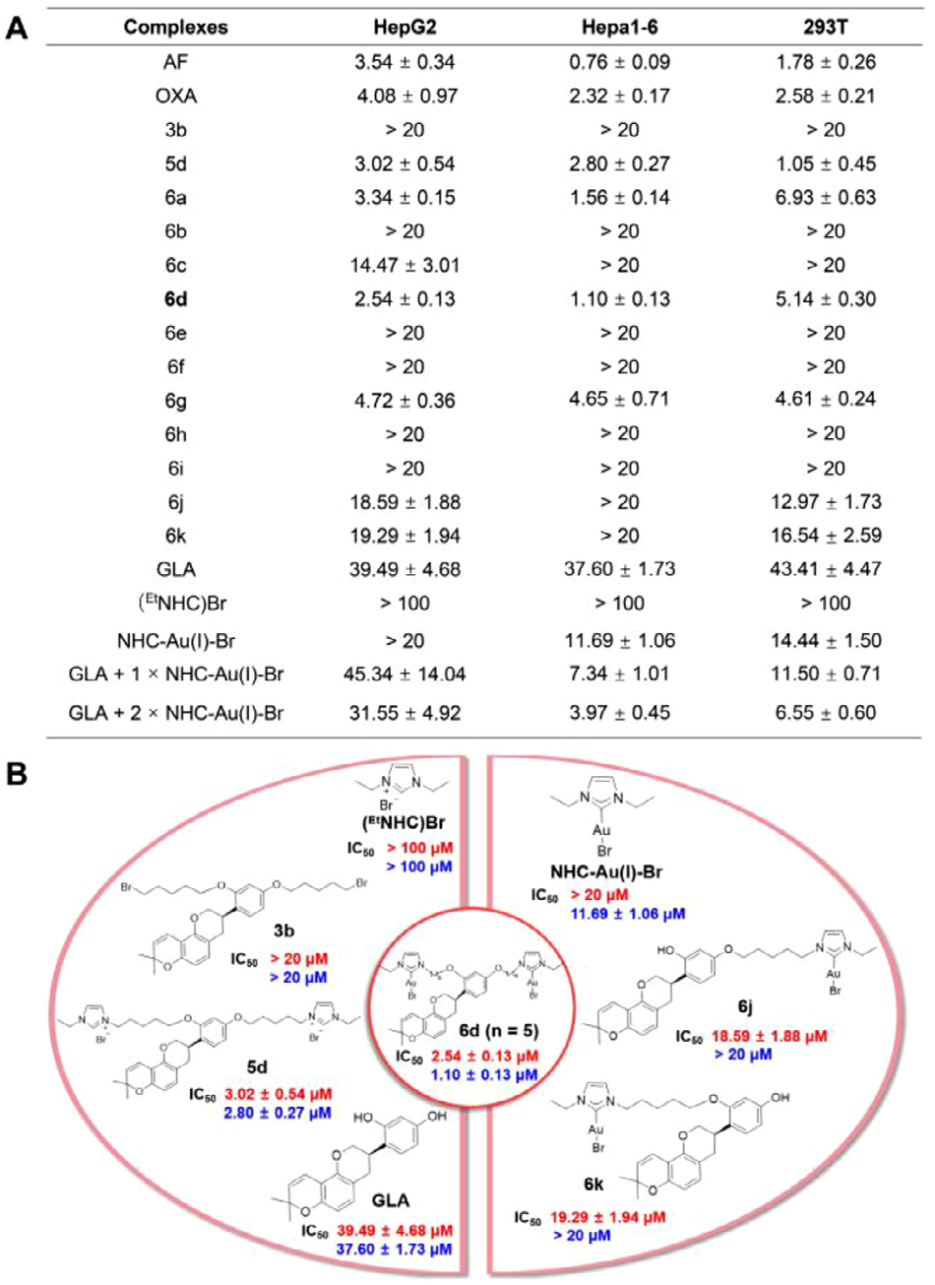
Antiproliferative activity of the complexes. A) IC_50_ values of the complexes in HepG2, Hepa1‐6, and 293T cell lines (Mean ± SD [µM], 72 h MTT assay). B) IC_50_ values of complex **6d** and its possible decomposition products in HepG2 (red) and Hepa1‐6 (blue) cell lines at 72 h.

Glabridin‐gold(I) complexes are designed to restore the immunogenicity of tumor cells through the ICD effect, requiring the release of DAMPs from dying cells to enhance antigen presentation.^[^ ^27^ ^]^ Therefore, the dominant complex in this study was selected based on its ability to promote tumor cell death. The methylthiazolyldiphenyl‐tetrazolium bromide (MTT) assay was used to evaluate the antiproliferative activity of these complexes on liver cancer cell lines (HepG2 and Hepa1‐6) and the normal kidney cell line (293T) (**Figure** **1**A). Nine glabridin‐gold(I) complexes were tested to identify the dominant complex, with AF and OXA serving as positive controls. After 72 h, complex **6d** (IC_50_, 2.54 ± 0.13 µM) demonstrated superior antiproliferative activity compared to AF (IC_50_, 3.54 ± 0.34 µM) and OXA (IC_50_, 4.08 ± 0.97 µM) in the human liver cancer HepG2 cell line. Notably, complex **6d** also exhibited lower cytotoxicity in the 293T cell line (IC_50_, 5.14 ± 0.30 µM) compared to liver cancer cell lines. Complex **6d** could be sufficiently dissolved under the experimental conditions of this study according to the solubility (Figure S40, Supporting Information) and log *P_ow_* analysis in experimental section. Additionally, GLA, NHC‐Au(I)‐Br, their direct mixtures (1:1 and 1:2), ligands, and gold complexes **6j**–**6k** were analyzed as controls.

Complex **6d** displayed greater cytotoxicity than these groups, including the direct ligand mixtures (Figure 1B), suggesting that TrxR targeting by the gold center further enhanced its antitumor activity. Moreover, some imidazole salt derivatives have been reported to exhibit antitumor effects.^[^ ^28^ ^]^ In this study, NHC and GLA were linked via an alkane chain of optimal length to create an amphiphilic structure, which may explain the significant activity enhancement of ligand **5d**.^[^ ^29^ ^]^ Furthermore, the activity followed the order GLA < **5d** < **6d**, and **6d** was 16/34 times more toxic than GLA toward HepG2 and Hepa 1–6 cells respectively, indicating that the introduction of NHC‐Au(I) was crucial for enhancing the complex's antiproliferative activity. Additionally, these findings suggest that complex **6d**, in its intact molecular form, demonstrates superior activity compared to its potential decomposition products. Based on this, we speculate that complex **6d** can reach the target site intact to exert its function. Therefore, complex **6d** was selected as the dominant complex and further investigated for its immune regulation mechanism in this study.

### 2.4 TrxR Inhibition, Oxidative Stress, and ICD Effect

**Figure 2:**
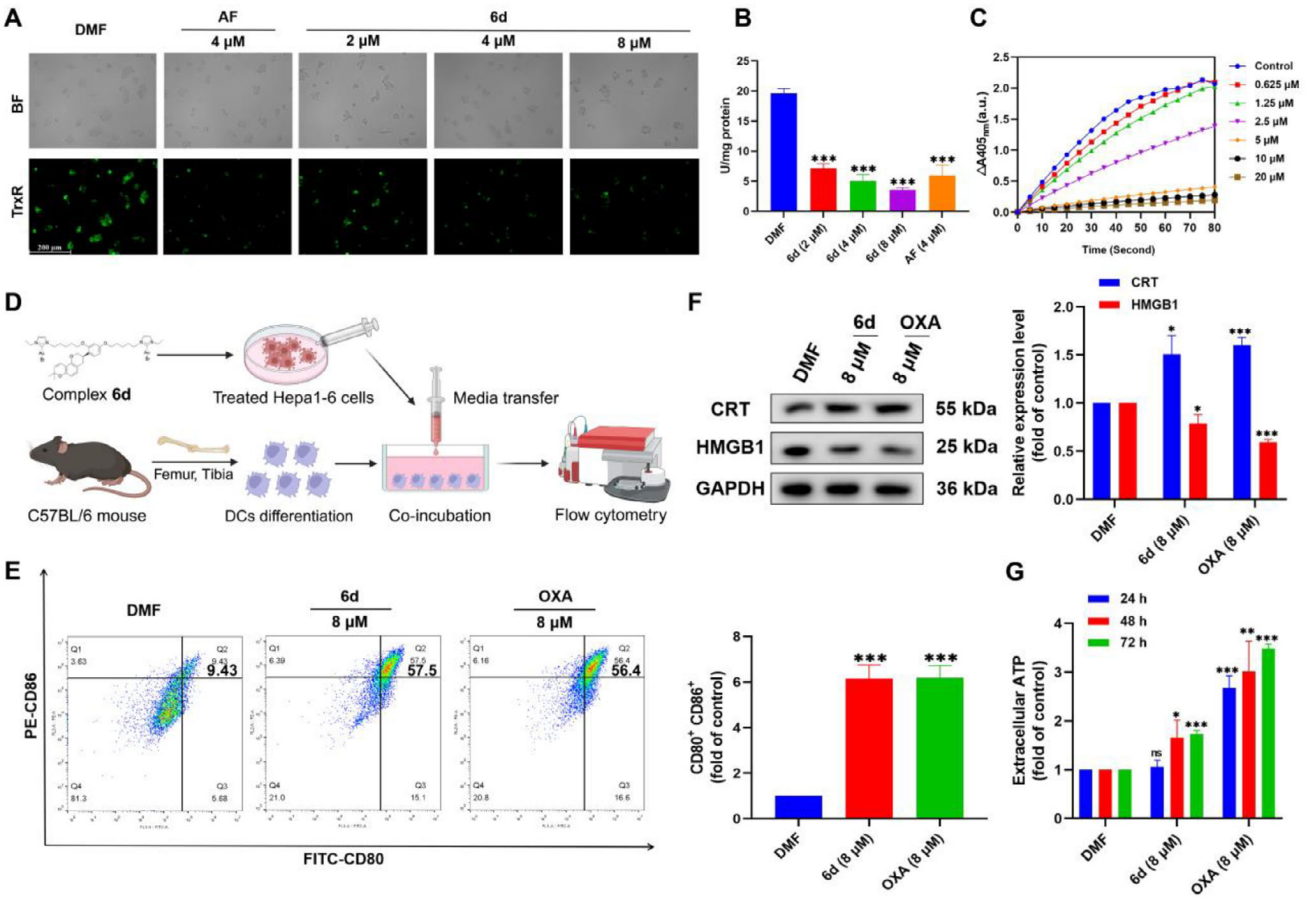
TrxR inhibitory activity and ICD effects of complex **6d**. A) Fluorescence analysis of TrxR activity in HepG2 cells after 24 h of **6d** treatment using a TrxR probe (scale bar = 200 µm). B) Intracellular TrxR activity assay in HepG2 cells after incubation with **6d**, 24 h measured using the TrxR Activity Assay Kit. C) Inhibition of purified TrxR by **6d**. D) Schematic workflow of the DC maturation assay (Image created with BioRender). E) Flow cytometry analysis of DC maturation following **6d** treatment for 24 h. F) Western blot analysis of CRT and HMGB1 expression after 24 h of **6d** treatment. G) Time‐dependent measurement of ATP release from HepG2 cells after **6d** treatment using the ATP Assay Kit. Data are presented as mean ± SD (n = 3); Student's *t*‐test, compared with DMF group; ns > 0.05, *p < 0.05, **p < 0.01, and ***p < 0.001.

Gold complexes are known to inhibit TrxR activity by binding to cysteine thiol/selenocysteine selenol at the enzyme's active sites.^[^ ^11b^ ^]^ The cellular thermal shift assay (CETSA) and isothermal dose‐response (ITDR) assay showed the significant binding between **6d** and target TrxR (Figure S41, Supporting Information). Then, to assess the TrxR inhibition ability of complex **6d**, the TrxR probe (**Figure** **2**A), TrxR activity assay kit (Figure 2B), and 5,5′‐dithiobis (2‐nitrobenzoic acid) (DTNB) assay (Figure 2C) were used separately. The results demonstrated that complex **6d** suppressed TrxR activity in a concentration‐dependent manner. Additionally, western blot analysis showed that complex **6d** did not downregulate TrxR expression levels (Figure S42, Supporting Information), indicating that it inhibits TrxR activity by binding to the enzyme's active site rather than degrading the protein. TrxR suppression can lead to ROS upregulation by disrupting redox homeostasis in cancer cells.^[^ ^30^ ^]^ Therefore, we examined whether ROS levels increased in cancer cells following treatment with complex **6d**. After 6 h of treatment, both dihydroethidium (DHE) and 2′,7′‐dichlorodihydrofluorescein diacetate (DCFH‐DA) probes indicated an elevated ROS level (Figure S43, Supporting Information). Since cancer cells generally maintain a higher redox balance for metabolism than normal cells, they are more susceptible to oxidative stress. Consequently, increased ROS levels may result in further cellular damage due to oxidative stress in HepG2 cells.

Oxidative stress can also lead to mitochondrial damage by reducing mitochondrial membrane potential (MMP).^[^ ^31^ ^]^ After 24 h of treatment with complex **6d**, the 5,5′,6,6′‐tetrachloro‐1,1′,3,3′‐tetraethyl‐imidacarbocyanine iodide (JC‐1) probe indicated decreased MMP in HepG2 cells, as reflected by a reduction in aggregates (red) and an increase in monomers (green) (Figure S44A, Supporting Information). Flow cytometry analysis further confirmed that complex **6d** promoted mitochondrial damage in HepG2 cells after 24 h (Figure S44B,C, Supporting Information). Mitochondrial damage can lead to apoptosis, and concentration‐dependent apoptosis was observed through annexin V‐EGFP/PI staining (Figure S44D, Supporting Information). Moreover, oxidative stress can induce ERS by accumulating unfolded proteins in the endoplasmic reticulum (ER). Since the ER serves as an essential calcium ion storage pool, ERS can trigger calcium release from the ER into the cytoplasm. This calcium release was detected using the Fluo‐4 AM probe in HepG2 cells after 24 h of **6d** treatment (Figure S45A, Supporting Information). Furthermore, ERS can upregulate the expression of related proteins, such as C/EBP homologous protein (CHOP) and calnexin. Western blot and immunofluorescence analyses confirmed these findings in HepG2 cells after 24 h of **6d** treatment (Figure S45B–F, Supporting Information).

The ROS/ERS pathway has been reported to induce the ICD effect by stimulating DAMPs, including adenosine triphosphate (ATP) excretion, calreticulin (CRT) exposure, and high mobility group box 1 (HMGB1) release.^[^ ^32^ ^]^ These DAMPs promote the maturation of DCs and the presentation of tumor antigens, facilitating cytotoxic T lymphocyte activation.^[^ ^11b^ ^]^ To simulate the in vivo DC maturation regulation of complex **6d**, immature DCs were extracted and isolated from the bone marrow of the leg bones of immunocompetent mice. The isolated immature DCs were then incubated in the medium of Hepa1‐6 cells treated with **6d** (Figure 2D). Flow cytometry analysis demonstrated that complex **6d** significantly induced DC maturation and CRT exposure after 24 h (Figure 2E and Figure S46A, Supporting Information). Immunofluorescence imaging further confirmed CRT exposure on the cell membrane and HMGB1 release from the cell nucleus following 24 h of **6d** treatment (Figure S46B,C, Supporting Information). Western blot analysis also showed CRT upregulation and HMGB1 downregulation in HepG2 cells (Figure 2F). CRT and HMGB1 function as “eat me” and “present me” signals in the ICD effect, respectively.^[^ ^27^ ^]^ After 48 h of treatment, ATP secretion into the extracellular medium was detected using an ATP assay kit (Figure 2G). DAMPs support mature DCs in presenting antigens, promoting T cell infiltration and stimulation. Therefore, complex **6d** enhances the immunogenicity of liver cancer cells, thereby inducing a tumor immune response. Notably, compared to CRT exposure and HMGB1 secretion observed after 24 h, ATP release required 48 h, serving as a “find me” signal. This result suggests that complex **6d** requires at least 48 h to induce a complete DAMP response.

### 2.5 MAPK Inhibition and PD‐L1 Downregulation

**Figure 3:**
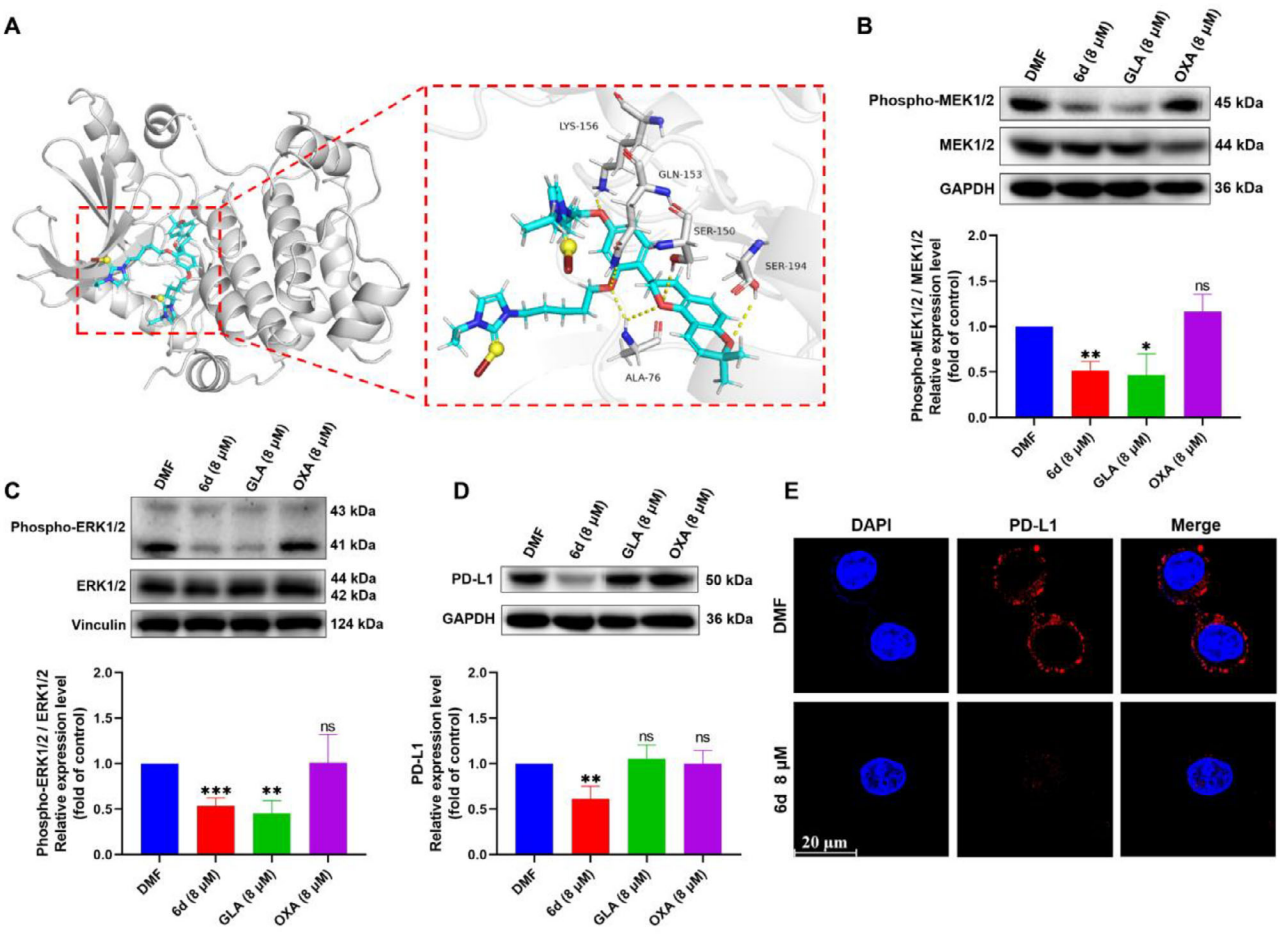
Inhibition of the MAPK pathway and PD‐L1 expression by complex **6d**. A) 3D binding model analysis of MEK1 (white) with **6d** (cyan), showing key residues as white sticks and H‐bonds as yellow dashed lines (docking score: −6.324 kcal mol^−1^). B) Western blot analysis of phosphorylated MEK1/2 levels after 24 h of **6d** treatment. C) Western blot analysis of phosphorylated ERK1/2 levels following 24 h incubation with **6d**. D) Western blot analysis of PD‐L1 expression after 24 h of **6d** treatment. E) Immunofluorescence analysis of PD‐L1 inhibition on the HepG2 cell surface following **6d** treatment (scale bar = 20 µm). Data are presented as mean ± SD (n = 3); Student's *t*‐test, compared with DMF group; ns > 0.05, *p < 0.05, **p < 0.01, and ***p < 0.001.

GLA, the ligand used in complex **6d**, has been reported to significantly suppress the expression of phosphorylated MEK1/2 and ERK1/2 in the HepG2 cell line.^[^ ^19a,b^ ^]^ To further explore the interaction between **6d** and MEK1, molecular docking was performed using MEK1 protein (PDB: 3ZLX). In the docking model, the protein was represented as a white cartoon, while complex **6d** was illustrated with cyan sticks, and key residues were highlighted with white sticks (**Figure** **3**A). Docking analysis revealed that **6d** exhibited strong binding energy with MEK1 (a lower docking score indicates stronger binding energy, and a score below −5 kcal mol^−1^ is considered strong). Complex **6d** was found to bind to the ATP‐binding active pocket of MEK1 through the formation of 6 H‐bonds with ALA‐76, SER‐150, GLN‐153, LYS‐156, and SER‐194. These interactions play a crucial role in stabilizing the complex‐protein interaction and may significantly influence the biological function of MEK1. Additionally, the CETSA and ITDR assay also demonstrated the significant binding between **6d** and target MEK1/2 (Figure S47, Supporting Information).

Western blot analysis was then performed to evaluate the ability of complex **6d** to inhibit the MAPK pathway in the HepG2 cell line. The results demonstrated that **6d** significantly inhibited the phosphorylation of MEK1/2 and ERK1/2 (Figure 3B,C). The inhibitory effect of **6d** on the MAPK pathway was comparable to that of GLA, whereas OXA showed no effect on MAPK pathway activation after 24 h of treatment. Furthermore, the activation of the MAPK pathway in tumors has been reported to play a key role in promoting an immunosuppressive tumor microenvironment. Therefore, in addition to stimulating an immune response, complex **6d** may also inhibit the activation of the immunosuppressive microenvironment, further enhancing its antitumor potential.

OXA has been used as an ICD inducer for liver cancer therapy; however, liver cancer has been found to develop immune escape mechanisms to resist OXA by upregulating PD‐L1 after prolonged drug administration. Inhibition of the MAPK pathway has been reported to prevent the upregulation of PD‐L1 in cancer cells.^[^ ^20b^ ^]^ Additionally, most TrxR inhibitors have been shown to affect PD‐L1 expression, with those capable of inactivating the MAPK pathway demonstrating a downregulatory effect on PD‐L1.^[^ ^33^ ^]^ Therefore, complex **6d** may also influence PD‐L1 expression.

Interestingly, western blot analysis revealed that **6d** downregulated PD‐L1 expression, whereas GLA alone did not exhibit this effect (Figure 3D). This finding suggests that the synergistic interaction between GLA and the gold center contributes to PD‐L1 downregulation. Immunofluorescence and flow cytometry analyses further confirmed that complex **6d** reduced PD‐L1 expression on the membrane surface of HepG2 cells after 24 h (Figure 3E; Figure S48, Supporting Information). Thus, **6d** downregulates both whole‐cell and membrane PD‐L1 expression through the crosstalk of multiple pathways, including the simultaneous inhibition of TrxR and MAPK pathways. Furthermore, western blot analysis also showed that OXA did not affect PD‐L1 expression after 24 h of treatment. This suggests that liver cancer cells may require multiple doses and a longer treatment cycle to develop OXA‐induced immune escape.

To further investigate the synergistic effects of GLA and the gold center on PD‐L1, this study explored the relevant downstream molecular mechanisms involving the TrxR and MAPK pathways (Figure S49, Supporting Information). TrxR inhibitors, such as AF, can upregulate phosphorylated ERK1/2 and downregulate phosphorylated Akt via oxidative stress.^[^ ^14^, ^34^ ^]^ Both phosphorylated ERK1/2 and Akt could promote the expression of hypoxia‐inducible factor 1α (HIF‐1α), which plays a crucial role in upregulating PD‐L1.^[^ ^35^ ^]^ Western blot analysis (Figure S49, Supporting Information) showed that AF promoted HIF‐1α expression and the subsequent upregulation of PD‐L1 by significantly enhancing ERK1/2 phosphorylation, although AF simultaneously inhibited Akt phosphorylation. Contrary to AF and similar to GLA, complex **6d** downregulated ERK1/2 phosphorylation by inhibiting the MAPK pathway. In addition, GLA at 8 µM did not significantly affect Akt phosphorylation, whereas **6d** inhibited Akt phosphorylation like AF. Consequently, complex **6d** significantly inhibited the expression of HIF‐1α and PD‐L1. These results suggest that the synergy between the gold center and GLA leads to downregulation of PD‐L1 expression by concurrently inhibiting the downstream activities of the TrxR and MAPK pathways, including phosphorylation of ERK1/2 and Akt.

### 2.6 Inhibition of MAPK Pathway and PD‐L1 Expression by **6d** In Vivo

**Figure 4:**
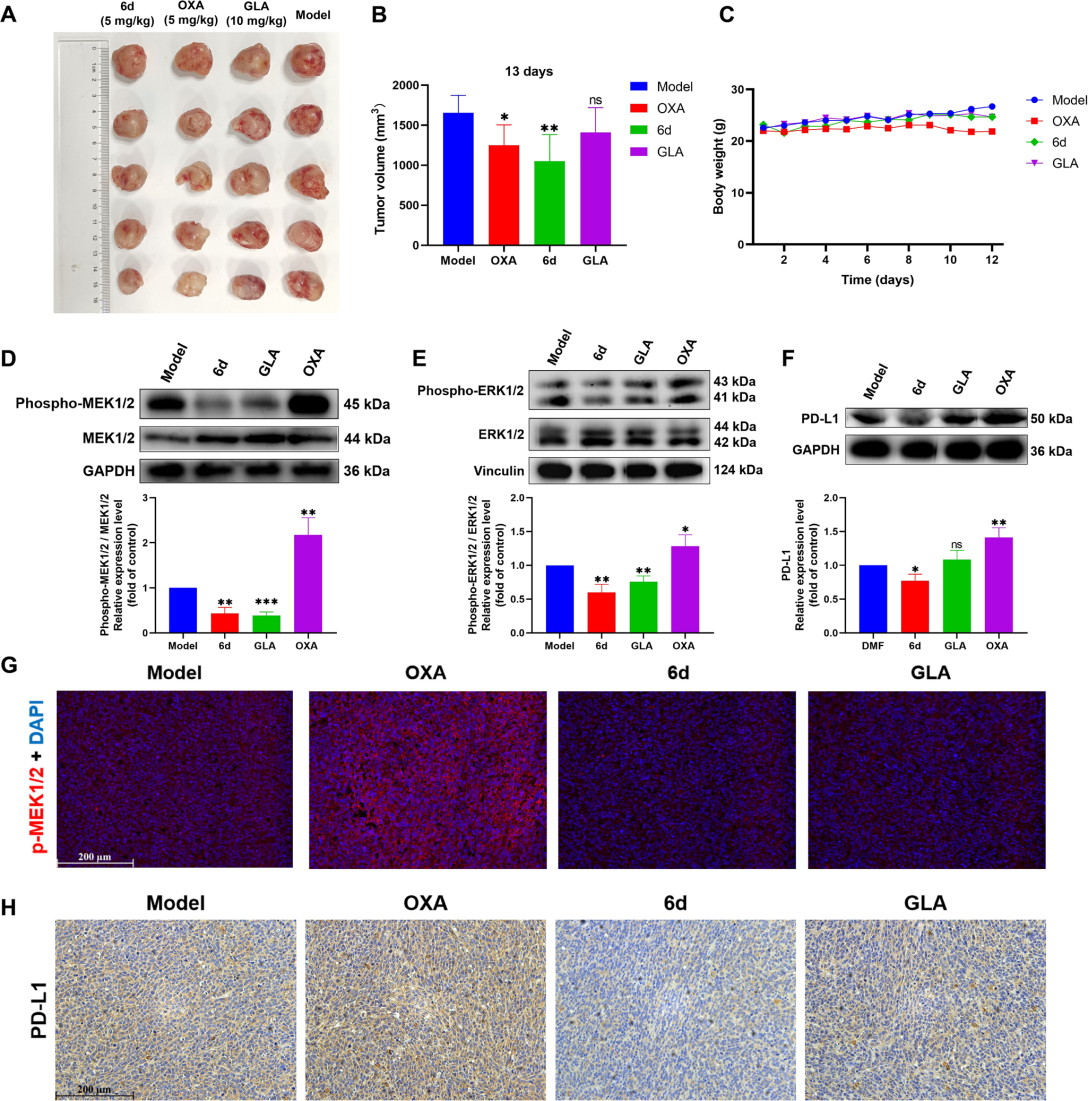
In vivo inhibition of the MAPK pathway and PD‐L1. A) Collected tumor images from mice treated with normal saline (Model group), OXA (OXA group), complex **6d** (**6d** group), and GLA (GLA group). B) In vitro tumor volume recorded after tumor collection on day 13 (n = 5). C) Daily recorded body weight of mice (n = 5). D) Western blot analysis of phosphorylated MEK1/2 levels in tumor tissue (n = 3). E) Western blot analysis of phosphorylated ERK1/2 levels in tumor tissue (n = 3). F) Western blot analysis of PD‐L1 expression in tumor tissue (n = 3). G) Immunofluorescence analysis of phosphorylated MEK1/2 in tumor tissue (scale bar = 200 µm). H) Immunohistochemistry analysis of PD‐L1 expression in tumor tissue (scale bar = 200 µm). Data are presented as mean ± SD; Student's *t*‐test, compared with Model group; ns > 0.05, *p < 0.05, **p < 0.01, and ***p < 0.001.

Based on the analysis of in vitro results, in vivo experiments were conducted using C57BL/6 mice bearing Hepa1‐6 tumors to evaluate the ability of complex **6d** to remodel the immunosuppressive microenvironment and restore tumor immunogenicity. Acute toxicity experiment showed that 5 mg kg^−1^ was a safe dose for complex **6d** (Figure S50, Supporting Information). Therefore, mice were administered intraperitoneal injections of 5 mg kg^−1^ of **6d** and OXA every two days, while GLA was injected simultaneously at a dose of 10 mg kg^−1^. GLA has been reported to exhibit antitumor activity, with 10 mg kg^−1^ being the suggested minimum effective dose.^[^ ^36^ ^]^ Normal saline was used as a negative control in the model group. Since the ICD effect requires tumor cell death to induce DAMPs and restore immunogenicity, tumor volume changes were recorded to assess the extent of induced tumor cell death through statistical significance analysis. After 11 days of treatment, **6d** treatment resulted in a significant reduction in tumor volume growth compared with the model group (Figure S51, Supporting Information). Given that in vitro experiments demonstrated that **6d** required 48 h to release ATP for complete DAMP induction, tumor and organ tissues were collected for further analysis on day 13. Hematoxylin and eosin (H&E) staining results confirmed that all drug‐treated groups induced tumor tissue damage (Figure S52A, Supporting Information). Both **6d** and OXA exhibited significant tumor growth inhibition compared with the model group after 13 days of treatment, whereas GLA did not show an obvious effect on tumor growth (**Figure** **4**A,B). These findings indicate that, consistent with in vitro results, the addition of the gold(I) ligand in **6d** enhanced its antitumor activity compared with GLA alone. Furthermore, statistical analysis revealed that **6d** and GLA had no significant effect on mice body weight, whereas the OXA group showed a decrease in body weight after nine days of treatment (Figure 4C). Kaplan‐Meier curves showed the 100% survival of **6d** group (Figure S53, Supporting Information). Additionally, H&E staining results demonstrated that **6d** and GLA did not cause noticeable damage to organ tissues, while OXA exhibited an abnormal effect on lung tissue (Figure S52B, Supporting Information). Therefore, complex **6d** may have lower in vivo toxicity compared to OXA, making it a potentially safer therapeutic option.

Next, the effect of complex **6d** on the tumor immunosuppressive microenvironment was analyzed. Both complex **6d** and GLA significantly inhibited the activation of the MAPK pathway, including the phosphorylation of MEK1/2 and ERK1/2. In contrast, in vivo analysis showed that OXA promoted the phosphorylation of this pathway, as demonstrated by western blot (Figure 4D,E). Activation of the MAPK pathway has been reported to contribute to drug resistance following OXA treatment.^[^ ^37^ ^]^ Additionally, the upregulation of the MAPK pathway is associated with the activation of the tumor immunosuppressive microenvironment. These findings suggest that complex **6d** exerts a different impact on immunosuppressive microenvironment activation compared with OXA. Consistent with in vitro results, complex **6d** also inhibited in vivo PD‐L1 expression, as shown by western blot (Figure 4F). Moreover, immunofluorescence (Figure 4G) and immunohistochemistry (Figure 4H) respectively further confirmed the results of western blot. Multiple dosing and long‐term therapy lead to the activation of the immunosuppressive microenvironment to resist OXA in liver cancer, with PD‐L1 upregulation being a key feature of immune resistance. Notably, after 13 days of treatment, OXA upregulated PD‐L1 expression in vivo, indicating that OXA‐induced immunosuppressive microenvironment activation may have already occurred due to repeated dosing. Further analysis suggests that other mechanisms of acquired immune resistance, such as the activation of MDSCs, may have been triggered in the OXA group. Given the opposing effects observed, complex **6d** and GLA also warrant further investigation regarding their potential impact on the immunosuppressive microenvironment, particularly their role in overcoming immune resistance.

### 2.7 Inhibition of Immunosuppressive Cells by **6d** In Vivo

**Figure 5:**
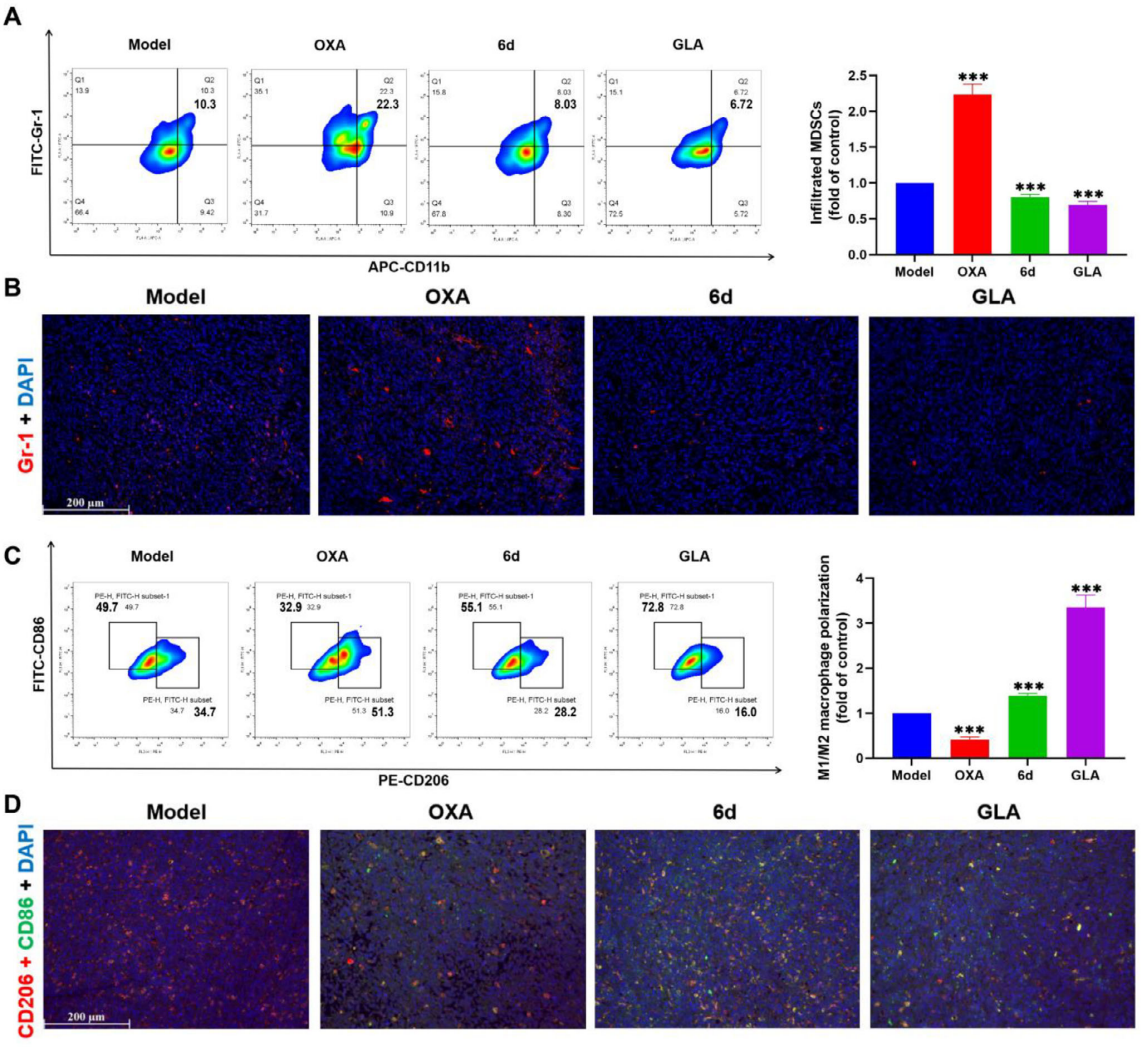
In vivo inhibition of the immunosuppressive microenvironment by **6d**. A) Flow cytometry analysis of infiltrated MDSCs in tumor tissue. B) Immunofluorescence analysis of MDSC infiltration in tumor tissue (scale bar = 200 µm). C) Flow cytometry analysis of macrophage polarization in tumor tissue. D) Immunofluorescence analysis of macrophage polarization in tumor tissue (scale bar = 200 µm). Data are presented as mean ± SD (n = 3); Student's *t*‐test, compared with Model group; ***p < 0.001.

Liver cancer cells have been reported to upregulate PD‐L1 and recruit MDSCs to resist OXA treatment, with Gr‐1 serving as a biomarker for MDSCs.^[^ ^38^ ^]^ After 13 days of treatment, in addition to PD‐L1 upregulation, the OXA group also exhibited a higher level of MDSC infiltration compared with the model group, as demonstrated by flow cytometry, immunofluorescence, and immunohistochemistry analyses (**Figure** **5A,B**; Figure S54, Supporting Information). The increased infiltration of MDSCs can lead to the activation of other immunosuppressive cells, such as Tregs and M2‐type macrophages.^[^ ^39^ ^]^ Immunohistochemistry results further confirmed the presence of a higher number of infiltrated Tregs in the tumor tissue of the OXA group, as indicated by Foxp3 detection (Figure S54, Supporting Information).^[^ ^40^ ^]^

Additionally, flow cytometry analysis of the M1/M2 macrophage ratio (Figure 5C) revealed that OXA promoted M2‐type polarization of macrophages, which serve as another class of immunosuppressive cells that support tumor progression. Moreover, immunofluorescence analysis (Figure 5D) of merged images of M1‐type macrophages (CD86, green) and M2‐type macrophages (CD206, red) showed a red‐biased merge in the OXA group, further confirming M2‐type macrophage polarization. In contrast, both **6d** and GLA significantly promoted M1‐type macrophage polarization while reducing the infiltration of MDSCs and Tregs in the tumor microenvironment. MDSCs and M2‐type macrophages can express Arg‐1, which contributes to the formation of immune response barriers within the tumor microenvironment.^[^ ^22^, ^41^ ^]^ Notably, immunofluorescence analysis (Figure S55, Supporting Information) demonstrated that **6d** and GLA significantly resulted in downregulation of Arg‐1 expression, and OXA promoted the expression of Arg‐1.

### 2.8 Stimulation of Antitumor Immune Response by **6d** In Vivo

**Figure 6:**
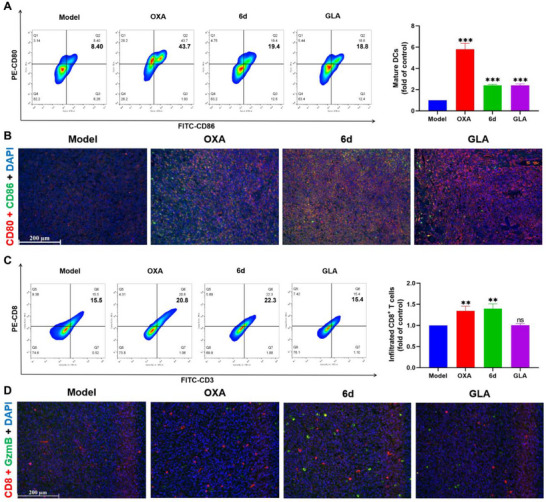
In vivo stimulation of the antitumor immune response by **6d**. A) Flow cytometry analysis of mature DCs in the spleen. B) Immunofluorescence analysis of mature DCs in tumor tissue (scale bar = 200 µm). C) Flow cytometry analysis of infiltrated CD8^+^ T cells in tumor tissue. D) Immunofluorescence analysis of CD8^+^ T cell activation in tumor tissue (scale bar = 200 µm). Data are presented as mean ± SD (n = 3); Student's *t*‐test, compared with Model group; ns > 0.05, **p < 0.01, and ***p < 0.001.

Then, the effects of **6d** on the antitumor immune response were evaluated by comparison with the OXA and GLA groups. According to flow cytometry and immunofluorescence results, **6d**, OXA, and GLA induced DC maturation in spleen (**Figure** **6**A) and tumor (Figure 6B) tissues. Immunofluorescence analysis showed that both **6d** and OXA promoted CRT exposure, whereas GLA did not induce CRT exposure on the cell membrane (Figure S56, Supporting Information). Thus, GLA may not trigger DAMPs for antigen presentation. Previous studies have shown that MAPK pathway activation is inversely correlated with DC maturation hallmarks.^[^ ^20a^ ^]^ Therefore, GLA may directly stimulate DC maturation by inhibiting MAPK pathways without DAMPs. Additionally, flow cytometry (Figure 6C) and immunohistochemistry (Figure S57, Supporting Information) analyses indicated that GLA did not enhance T lymphocyte infiltration compared with **6d** and OXA. These findings further suggest that GLA did not induce antigen presentation in vivo for an antitumor immune response. In contrast, both **6d** and OXA promoted T lymphocyte infiltration in vivo through antigen presentation induced by DAMPs.

However, infiltrated T cells in tumor tissues must express GzmB to exert an antitumor immune response.^[^ ^42^ ^]^ While OXA significantly increased T lymphocyte infiltration, GzmB expression levels did not increase obviously in the OXA group compared with the model group (Figure 6D). These results suggest that activated tumor immunosuppressive microenvironments may inhibit T lymphocyte activation after OXA treatment. Compared with the OXA group, complex **6d** significantly upregulated GzmB expression (Figure S58, Supporting Information), stimulating an antitumor immune response in addition to increasing infiltrated T lymphocytes.

## 3 Conclusion

In this study, we propose an immunomodulatory strategy based on the simultaneous inhibition of TrxR and MAPK to enhance tumor immunogenicity and remodel the immunosuppressive microenvironment. We developed a novel metal‐based immunomodulatory agent, **6d**, by designing and synthesizing a gold complex incorporating the natural product GLA. Gold complex **6d** promotes DC maturation and tumor antigen presentation through a ROS‐mediated ERS‐driven ICD effect following TrxR inhibition. Concurrently, MAPK pathway inhibition by **6d** suppresses the infiltration of MDSCs and Tregs while inducing M1‐type macrophage polarization. Notably, the combination of a gold center with GLA prevents tumor cells from expressing PD‐L1 and promotes T cells to produce GzmB. These findings highlight the promising potential of **6d** in combination strategies of cancer immunotherapy by eliminating immune barriers within the tumor microenvironment. This strategy offers a feasible approach for designing and synthesizing metal‐based immunomodulatory agents that can avoid activating the immunosuppressive microenvironment. Furthermore, it suggests that the simultaneous inhibition of TrxR and MAPK may be a novel avenue for further exploration in immunotherapy.

## 4 Experimental Section

### Materials and Instrumentations

All chemical reagents, solvents, raw materials, and consumables were sourced from commercial suppliers in China and utilized directly without additional purification. ^1^H and ^13^C NMR spectra were characterized by the Bruker Avance III HD 500 MHz spectrometer (Karlsruhe, Germany). ESI‐MS data were obtained by Thermo Scientific TSQ Quantis (MA, USA) and Thermo Fisher Q Exactive (Germany). The ≥ 95% purity of the gold complex was proved through HPLC utilizing a Waters ACQUITY (MA, USA) fitted with a C18 column.

Calreticulin Rabbit Polyclonal antibody, HMGB1 Rabbit Recombinant antibody, Calnexin Rabbit Polyclonal antibody, Granzyme B Rabbit Polyclonal antibody, PD‐L1/CD274 Mouse Monoclonal antibody, HIF‐1 alpha Polyclonal antibody, Alpha Tubulin Mouse Monoclonal antibody, HRP‐conjugated Goat Anti‐Mouse IgG (H+L), HRP‐conjugated Goat Anti‐Rabbit IgG (H+L), CoraLite488‐conjugated Goat Anti‐Rabbit IgG (H+L), and CoraLite594‐conjugated Goat Anti‐Rabbit IgG (H+L) were purchased from Proteintech Group, Inc. (USA). Recombinant Anti‐GAPDH antibody (Rabbit mAb) was purchased from Wuhan Servicebio Technology Co., Ltd (China). DDIT3/CHOP Rabbit polyclonal Antibody was purchased from Affinity Biosciences (Australia). Anti‐TXNRD1 Rabbit monoclonal Antibody was purchased from Boster Biological Technology (USA). p‐MEK‐1/2 antibody was purchased from Santa Cruz Biotechnology, Inc (USA). Phospho‐Erk1 (T202) + Erk2 (T185) Recombinant Rabbit Monoclonal Antibody, Phospho‐MEK1/2 (S218 + S222) Recombinant Rabbit Monoclonal Antibody, MEK1/2 Recombinant Rabbit Monoclonal Antibody, and Vinculin Recombinant Rabbit Monoclonal Antibody were purchased from Hangzhou HUABIO Biotechnology (China). AKT1/2/3 Antibody, Phospho‐Akt (Ser473) Antibody and ERK1/2 Rabbit Monoclonal Antibody was purchased from Abmart (Shanghai) Co., Ltd. Anti‐Mouse Ly‐6G and Ly‐6C antibody were purchased from BD Biosciences (USA). CD11c Monoclonal Antibody (N418), CD80 (B7‐1) Monoclonal Antibody (16‐10A1), CD86 (B7‐2) Monoclonal Antibody (GL1), CD3e Monoclonal Antibody (145‐2C11), CD4 Monoclonal Antibody (GK1.5), CD8a Monoclonal Antibody (53‐6.7), CD45 Monoclonal Antibody (30‐F11), CD11b Monoclonal Antibody (M1/70), CD206 (MMR) Monoclonal Antibody (MR6F3), Dulbecco's modified eagle medium (DMEM), and RPMI 1640 medium were purchased from Thermo Fisher Scientific Inc (USA). β‐NADPH, MTT, Collagenase IV, and DNase I were purchased from Shanghai yuanye Bio‐Technology Co., Ltd (China). DHE, hydrochloride (DAPI), protease inhibitor, and JC‐1 Mitochondrial membrane potential assay kit were purchased from APExBIO Technology LLC (USA). DCFH‐DA, Triton X‐100, and Oxidized TrxR Activity Assay Kit were purchased from Beijing Solarbio Science & Technology Co., Ltd (China). Fluo‐4 AM probe, Enhanced ATP Assay Kit, and Red Blood Cell Lysis Buffer were purchased from Beyotime Biotechnology (China). Purified TrxR was provided by Prof. Jianqiang Xu. The TrxR probe was synthesized by ourselves according to the previous research.^[^ ^43^ ^]^ DTNB was purchased from MedChemExpress (USA). Annexin V‐EGFP/PI Apoptosis Detection kit was purchased from Abbkine Scientific Co., Ltd (China). PMSF was purchased from Jiangsu Keygen Biotech Corp.,Ltd (China). Fetal bovine serum (FBS) was purchased from Genial Biological Inc (USA). Bovine serum albumin (BSA) was purchased from Guangzhou saiguo biotech Co., Ltd (China). Penicillin−streptomycin, phosphatase inhibitor, RIPA buffer, and BCA Protein Assay kit were purchased from New Cell & Molecular biotech Co., Ltd (China). rmGM‐CSF, and IL‐4 were purchased from Novoprotein Scientific Inc (China).

Flow cytometry analysis was performed by BD Accuri^TM^ C6 Plus Flow cytometer (USA). H&E staining, immunofluorescence and immunohistochemistry images were collected by Leica DM4B Upright Microscopes or Leica SP8 Confocal Laser Scanning Microscope (Germany). Fluorescent probe analysis was carried out by Leica DMi8 Inverted Microscopes (Germany). Western blot analysis was performed by Azure C500 Imaging System (USA). OD values were collected by ALLSHENG AMR‐100 Microplate Reader (China).

### Synthesis and Characterization

Synthesis and characterization of **3a‐3e**, **5a‐5k,** and **6a‐6k** are detailed in supporting information.

### Stability Analysis

5 mM of complex **6d** was incubated with DMSO‐*d_6_* or the mixed solution (DMSO‐*d_6_*/D_2_O = 9:1) or the same mixed solution with 5 mM of GSH for different times (0 – 72 h), and the ^1^H NMR spectra were acquired. The stability of AF was determined by the same method.

### 1‐Octanol/Water Partition Coefficients (log P_ow_)

The log *P_ow_* of **6d** was determined by shake flask method and ultraviolet‐visible spectrophotometry. Equal volumes of deionized water and 1‐octanol were placed in glass bottle and shaken on a flat shaker for 24 h to saturate the two phases. Then centrifuge for separation. Prepare the stock solution of **6d** (5 mM) with *N,N*‐dimethylformamide (DMF). Dilute the original solution with 1‐octanol‐saturated water to an appropriate concentration (0.2 ≤ A ≤ 0.8 at λ_max_) and record its ultraviolet‐visible spectrum (A_0_). Subsequently, the above‐mentioned 1‐octanol‐saturated water containing **6d** (7.5 mL) was mixed with water‐saturated 1‐octanol (7.5 mL). Shake at room temperature for 2 h, then centrifuge for separation. Record the ultraviolet‐visible spectrum (A_1_) of the 1‐octanol‐saturated water. Then calculate the partition coefficient as *P_ow_* = (A_0_ – A_1_)/A_1_. The procedure was repeated three times and the results are shown as mean ± standard deviation (SD). The results show that the log *P_ow_* of **6d** is – 0.03 ± 0.01.

### Solubility of **6d**

Weigh a certain amount of **6d**, dissolve it in a PBS: DMF = 200:1 solution, and mix to obtain the high‐concentration solution. Then dilute the original solution to prepare solutions of different concentrations. The ultraviolet absorbance values at the maximum absorption wavelength (237 nm) were measured in sequence, and the standard curve was plotted (Figure S40, Supporting Information). Prepare the saturated **6d** solution by the same method. After centrifugation, take the supernatant and dilute it 7 times. Then measure the absorbance at 237 nm and calculate the solubility for **6d** is 108 µg mL^−1^.

### Docking

Structure of active ingredient complexes were generated from Chemdraw 20.0, and imported into ChemBio3D 14.0 software to adjust the spatial conformation of active ingredients, calculate the optimization of energy, and save in mol2 format. After AutoDockTools 1.5.6 processing, the files were saved in pdbq format. The 3D crystal structure of the target protein was downloaded from the Uniprot or PDB database. The water molecule and organic matter in the target protein were removed by Notepad2, and then the target protein was imported into AutoDockTools1.5.6 for hydrogenation, charge distribution, and atomic type addition. The pdbqt format file was saved. AutoDockVina was used for covalent molecular docking, and the docking results were plotted with Pymol 2.6.1.

### Cell Viability Assay

All cells were incubated at 37 °C by DMEM complete medium (containing 1% penicillin−streptomycin and 12% FBS) in 5% CO_2_ incubator. 2–5 × 10^3^ cells were cultured in 96‐well plates and each well contained 100 µL complete medium. OXA was dissolved in water, and all other complexes were dissolved in DMF. After cells adhered in the wells, different concentrations of complexes were diluted in 100 µL complete medium and added in the wells. The final volume in the each well was 200 µL and the content of DMF was maximum 0.5%. After 72 h incubation, the antiproliferative activity was tested by MTT assay.

### Purified TrxR Activity Assay

Different concentrations of complex **6d** solution and 1.8 U purified TrxR were added to the wells (25 µL per well separately). Consequently, the reaction solution (20 mg mL^−1^ BSA, 500 mM EDTA‐2Na, and 40 mg mL^−1^ NADPH, diluted in a 1:1 mixture of PBS and water) was added to the 96 wells (225 µL per well). Finally, 25 mg mL^−1^ DTNB solution was added (25 µL per well) and OD values were immediately measured at 405 nm. The linear growth at 405 nm was calculated to show the purified TrxR activity. Final concentrations of complex **6d** were 0.625, 1.25, 2.5, 5, 10, 20 µM.

### Intracellular TrxR Activity Assay

1 × 10^6^ HepG2 cells were cultured in 6 cm culture dishes, and treated with DMF (0.1%), AF (4 µM) or different concentrations of **6d** (2, 4, 8 µM) after cell adhesion. 24 h later, the intracellular TrxR activity was analyzed by the TrxR activity detection kit.

In addition, intracellular TrxR activity was detected by TrxR probe. 5 × 10^4^ HepG2 cells were seeded in 12‐well plate. After 24 h, the complete medium was removed, and the each well was washed twice by phosphate buffer saline (PBS). 10 µM TrxR probe (dissolved in PBS) was added for 1 h incubation. Then, the probe was removed, and washing twice was performed by PBS. The fluorescence microscopy was applied to capture the cell images under the channel of FITC.

### ROS Levels Assay

HepG2 cells were seeded in 12‐well plate (5 × 10^4^ cells per well). 4 mM *N*‐acetylcysteine (NAC) was applied for 1 h pretreatment. DMF (0.1%), AF (4 µM) or different concentrations of **6d** (2, 4, 8 µM) were added in the wells for 6 h incubation. 10 µM DHE or DCFH‐DA (dissolved in DMEM) probes were applied for 30 min staining. The cells were photographed by the fluorescence microscopy after washing twice.

### MMP Measurement

12‐well plate was applied to incubate HepG2 cells (5 × 10^4^ cells per wells). After overnight, cells were treated with DMF (0.1%), AF (4 µM) or different concentrations of **6d** (2, 4, 8 µM) for 24 h. 2.5 µg mL^−1^ JC‐1 probe was added in the wells after removing original liquid. 15 min later, PBS was applied to wash cells for two times. Then, the fluorescence images were captured by the fluorescence microscopy.

### Apoptosis Assay

4 × 10^5^ HepG2 cells was seeded in 6‐well plates for overnight incubation. DMF (0.1%), AF (4 µM) or different concentrations of **6d** (2, 4, 8 µM) were added in the wells for 24 h. PBS was applied to wash cells for two times, and cells were collected subsequently. After washing twice by PBS, Annexin V‐EGFP/PI Apoptosis Detection kit was used to dye the cells. Then, the apoptosis ratio was analyzed by flow cytometer.

### ATP Release Assay

6‐well plate was applied to incubate 2 × 10^5^ HepG2 cells. DMF (0.1%), OXA (8 µM) or **6d** (8 µM) were added in the wells for 24, 48 or 72 h. After treatment, the liquid was collected for extracellular ATP release assay by ATP Assay kit.

### DC Maturation Assay

The monocytes were extracted from the leg bone marrow of a C57BL/6 mouse and incubated in the 1640 complete medium with rmGM‐CSF (20 ng mL^−1^) and IL‐4 (20 ng mL^−1^). The medium was replaced partially every other day, and immature DCs were differentiated after 7 days. DMF (0.1%), OXA (8 µM) or **6d** (8 µM) were utilized to treat 4 × 10^5^ Hepa1‐6 cells in 6‐well plates for 24 h. The medium from treated Hepa1‐6 cells was collected and coincubated with the differentiated DCs for 24 h. After cells collection, the coincubated DCs were labeled with CD11c antibody, CD80 antibody and CD86 antibody. Then, the DC maturation ratio was analyzed by a flow cytometer.

### Western Blot Analysis

HepG2 cells were incubated in 6 cm culture dishes and treated with different complexes. The treated cells were lysed with lysis buffer (RIPA:PMSF:phosphatase inhibitor:protease inhibitor = 100:1:1:1). The lysed cells were further broken by sonication, and proteins were isolated by centrifugation. The protein concentrations were detected by BCA assay. 10% SDS PAGE gel was usually applied to separate the different kDa of proteins, and more than 200 kDa of proteins were separated using 8% SDS PAGE gel. After seperation, proteins were transferred onto polyvinylidene difluoride (PVDF) membranes. The target proteins were labeled by various specific primary antibodies at 4 °C overnight. Then, the membranes were treated with anti‐rabbit or anti‐mouse secondary antibody for 2 h. Tris buffered saline with tween 20 (TBST) was applied to wash the membranes after each operation above. The target proteins’ bands were detected by the enhanced chemiluminescence (ECL) procedure.

### CETSA and ITDR Assay

The treated HepG2 cells in 10 cm culture dishes were collected and washed with PBS. The collected cells were repeatedly frozen and thawed for 3 times by liquid nitrogen and 37 °C water bath. Then, proteins were isolated by centrifugation. The isolated proteins were divided into different tubes and incubated with same concentration of **6d** at different temperature. Western blot was applied for CETSA by analyzing the band intensity of different temperature groups.

The temperature, which results in significant band differences between **6d** and control group, was selected for ITDR assay. The isolated proteins were divided into different tubes and incubated with different concentration of **6d** at same temperature. The binding between **6d** and target protein was assessed by analyzing the band intensity of different concentration groups.

### Immunofluorescence Analysis

5 × 10^4^ HepG2 cells were treated with complexes in 24‐well plates for 24 h. Subsequently, cells were fixed with 4% paraformaldehyde. Various specific antibodies were added for incubation at 4 °C overnight after cells were permeabilized with Triton X‐100 (except for membrane proteins) and blocked with BSA. Then, the cells were incubated with fluorescently‐labeled secondary antibodies in darkness for 2 h. After that, DAPI was added to stain nuclear in darkness. PBS was applied for washing after each operation above (Tris buffered saline (TBS) was applied for washing if phosphorylated protein was analyzed). The cell images were captured by the fluorescence microscopy.

### Flow Cytometric Analysis

4 × 10^5^ HepG2 cells were incubated in 6‐well plates, and complexes were added in the wells. The cells were collected after incubation, and the treated cells were labeled with probes or fluorescently‐labeled antibodies. A flow cytometer was applied to analyze the labeled cells.

### In Vivo Antitumor Assays

All mice were 6–8 weeks old males and procured from Hangzhou Medical College, located in Hangzhou, China. All procedures involving animal experiments were conducted according to the guidelines approved by the institutional and local ethics committee of Nanjing University of Chinese Medicine (The assigned animal ethics approval number is 202308A006, and the accreditation number is SYXK‐(Su) 2018‐0049). 2 × 10^6^ Hepa1‐6 cells in 100 µL DMEM were subcutaneously seeded in underarms of C57BL/6 mice. After 6 days, these mice were divided randomly into four groups, which were Model (normal saline), OXA (5 mg kg^−1^), GLA (10 mg kg^−1^) and **6d** (5 mg kg^−1^) group respectively for intraperitoneal injection once every two days. Meanwhile, the mice weights and tumor volume were recorded, and the calculation formula of tumor volume was Volume = Width^2^ × Length/2. After 13 days, all mice were sacrificed for collection of tumors, hearts, livers, spleens, lungs and kidneys. Collagenase IV (1 mg mL^−1^) and DNase I (1 µg mL^−1^) were applied to digest a part of the tumor tissue into cell suspension, and lysis buffer was used to remove red blood cells. The prepared cells above were stained with antibodies (anti‐CD3e‐FITC, and anti‐CD8a‐PE; anti‐CD11c‐APC, anti‐CD80‐PE, and anti‐CD86‐FITC; anti‐CD45‐Percp, anti‐CD11b‐APC, and anti‐Ly‐6G/Ly‐6C(Gr‐1)‐FITC; anti‐CD11b‐APC, anti‐CD206‐PE, and CD86‐FITC). CD3^+^CD8^+^ T cells, CD80^+^CD86^+^ DCs, CD11b^+^Gr‐1^+^ MDSCs, CD206^−^CD86^+^ M1‐type macrophages and CD206^+^CD86^−^ M2‐type macrophages were analyzed by flow cytometry. Then, another part of tumor tissues were fixed with 4% paraformaldehyde, embedded in paraffin, cut into slices and stained with antibodies for immunofluorescence and immunohistochemistry analysis. H&E stain assays were applied for all tissue samples.

### Statistical Analysis

GraphPad Prism8 Software was employed to analyze data, and all calculated values were presented as the mean ± SD. All statistical analysis was performed after the experiment data were repeated at least three times. Unpaired two‐tailed Student's *t*‐test was used to compare two experimental groups, and significance was considered according to *p < 0.05, **p < 0.01, and ***p < 0.001 (ns = not significant).

## Conflict of Interest

The authors declare no conflict of interest.

## Supporting information



Supporting Information

## Data Availability

The data that support the findings of this study are available in the supplementary material of this article.
