# Supplementary material for: Glabridin‐Gold(I) Complex as a Novel Immunomodulatory Agent Targeting TrxR and MAPK Pathways for Synergistic Enhancement of Antitumor Immunity
Source: Adv Sci (Weinh). 2025 Aug 21;12(41):e04729. doi: 10.1002/advs.202504729 (PMC12591170; doi:10.1002/advs.202504729)
Supplement: Supplementary file 1 — Supporting Information [file ADVS-12-e04729-s001.docx]

**Supporting Information**

**Glabridin-Gold(I) Complex as a Novel Immunomodulatory Agent Targeting TrxR and MAPK Pathways for Synergistic Enhancement of Antitumor Immunity**

Zhaoran Wang,^+^ Meiyu Wang,^+^ Qiong Chen, Mengshi Wang, Fuwei Li, Lin Lv, Zhenfan Wen, Zhongren Xu, Yixia Yang, Chunyang Bi, and Wukun Liu*

**Table of Contents**

**Synthesis and Characterization** .................................................................................3

**Supporting Tables and Figures** ................................................................................13

**Author Contributions** .................................................................................................41

**Synthesis and Characterization**

1. Synthesis of **3a-3e**.

Synthesis of **3a-3c:** Glabridin (0.463 mmol) was dissolved in anhydrous dimethylformamide, then 1,4-dibromobutane, 1,5-dibromopentane or 1,6-dibromohexane (2.778 mmol) and potassium carbonate (1.850 mmol) were added. The mixture was kept under stirring for 2 h at room temperature, followed by extraction with dichloromethane and water. Organic phases were collected, and dried with anhydrous sodium sulfate. Silica gel (PE / EA) was applied for the purification of product.

Synthesis of **3d-3e:** Glabridin (1.230 mmol), 1,5-dibromopentane (1.850 mmol) and potassium carbonate (2.470 mmol) were dissolved in acetonitrile and refluxed at 55 ℃ for 6 h. Silica gel (PE / EA) was applied for the purification of product.

1.1. *(R)-3-(2,4-bis(4-bromobutoxy)phenyl)-8,8-dimethyl-3,4-dihydro-2H,8H- pyrano[2,3-f]chromene (****3a****)*. Colorless oil, yield 35.8 %.^1^H NMR (500 MHz, Chloroform-*d*) *δ* 7.03 (d, *J* = 8.2 Hz, 1H, Ar**H**), 6.85 (d, *J* = 8.2 Hz, 1H, CH=C**H**), 6.68 (d, *J* = 9.9 Hz, 1H, Ar**H**), 6.49-6.44 (m, 2H, Ar**H**), 6.39 (d, *J* = 8.2 Hz, 1H, Ar**H**), 5.58 (d, *J* = 9.9 Hz, 1H, C**H**=CH), 4.35 (ddd, *J* = 10.2, 3.5, 2.0 Hz, 1H), 4.01 (ddd, *J* = 12.1, 6.3, 3.6 Hz, 5H, OC**H**_2_), 3.60-3.54 (m, 1H), 3.50 (dt, *J* = 7.8, 6.6 Hz, 4H, BrC**H**_2_), 2.96 (ddd, *J* = 15.7, 10.8, 1.1 Hz, 1H), 2.86 (ddd, *J* = 16.0, 5.3, 2.0 Hz, 1H), 2.12-2.05 (m, 4H), 2.02-1.94 (m, 4H), 1.45 (s, 3H, C**H**_3_), 1.44 (s, 3H, C**H**_3_). ^13^C NMR (126 MHz, Chloroform-*d*) *δ* 158.99 (OAr**C**), 157.58 (OAr**C**), 152.02 (OAr**C**), 149.98 (OAr**C**), 129.32, 129.00, 127.80, 122.24, 117.10, 114.57, 110.00, 108.76, 105.07, 99.94, 75.71, 70.35 (O**C**H_2_), 67.11 (O**C**H_2_), 67.06 (O**C**H_2_), 33.53, 33.44, 31.65, 31.58, 30.88, 30.34, 29.83, 29.62, 29.58, 28.05, 27.97, 27.96, 27.76.

1.2. *(R)-3-(2,4-bis((5-bromopentyl)oxy)phenyl)-8,8-dimethyl-3,4-dihydro-2H,8H- pyrano[2,3-f]chromene* ***(3b)****.* Colorless oil, yield 64.2 %. ^1^H NMR (500 MHz, Chloroform-*d*) *δ* 7.00 (d, *J* = 8.2 Hz, 1H, Ar**H**), 6.82 (d, *J* = 8.2 Hz, 1H, CH=C**H**), 6.65 (d, *J* = 9.9 Hz, 1H, Ar**H**), 6.48-6.41 (m, 2H, Ar**H**), 6.36 (d, *J* = 8.2 Hz, 1H, Ar**H**), 5.56 (d, *J* = 9.9 Hz, 1H, C**H**=CH), 4.34 (d, *J* = 10.3 Hz, 1H), 4.04-3.92 (m, 5H, OC**H**_2_), 3.56 (td, *J* = 10.3, 5.0 Hz, 1H), 3.43 (dt, *J* = 13.0, 6.7 Hz, 4H, BrC**H**_2_), 2.95 (dd, *J* = 15.7, 10.9 Hz, 1H), 2.83 (dd, *J* = 15.7, 5.2 Hz, 1H), 1.93 (h, *J* = 7.2 Hz, 4H), 1.82 (h, *J* = 7.1 Hz, 4H), 1.67-1.59 (m, 4H), 1.43 (s, 3H, C**H**_3_), 1.42 (s, 3H, C**H**_3_). ^13^C NMR (126 MHz, Chloroform-*d*) *δ* 159.10 (OAr**C**), 157.72 (OAr**C**), 152.00 (OAr**C**), 150.00 (OAr**C**), 129.32, 128.99, 127.75, 122.14, 117.12, 114.66, 109.99, 108.71, 104.97, 99.99, 75.70, 70.38 (O**C**H_2_), 67.80 (O**C**H_2_), 67.78 (O**C**H_2_), 33.71, 33.66, 32.61, 32.48, 31.71, 30.87, 28.61, 28.55, 27.96, 27.74, 25.03, 25.00.

1.3. *(R)-3-(2,4-bis((6-bromohexyl)oxy)phenyl)-8,8-dimethyl-3,4-dihydro-2H,8H- pyrano[2,3-f]chromene (****3c****)*.Colorless oil, yield 57.9 %. ^1^H NMR (500 MHz, Chloroform-*d*) *δ* 7.00 (d, *J* = 8.5 Hz, 1H, Ar**H**), 6.82 (d, *J* = 8.2 Hz, 1H, CH=C**H**), 6.69 -6.60 (m, 1H, Ar**H**), 6.47-6.41 (m, 2H, Ar**H**), 6.38-6.33 (m, 1H, Ar**H**), 5.56 (dd, *J* = 9.9, 2.1 Hz, 1H, C**H**=CH), 4.33 (dd, *J* = 10.3, 3.0 Hz, 1H), 4.02-3.92 (m, 5H, OC**H**_2_), 3.55 (tt, *J* = 9.4, 3.8 Hz, 1H), 3.45-3.37 (m, 4H, BrC**H**_2_), 2.96 (dd, *J* = 15.8, 10.9 Hz, 1H), 2.83 (dd, *J* = 15.7, 5.1 Hz, 1H), 1.89 (dq, *J* = 13.1, 6.8 Hz, 4H), 1.80 (q, *J* = 7.0 Hz, 4H), 1.53-1.46 (m, 8H), 1.43 (s, 3H, C**H**_3_), 1.42 (d, *J* = 2.0 Hz, 3H, C**H**_3_). ^13^C NMR (126 MHz, Chloroform-*d*) *δ* 159.18 (OAr**C**), 157.80 (OAr**C**), 151.99 (OAr**C**), 150.00 (OAr**C**), 129.31, 128.98, 127.70, 122.03, 117.11, 114.68, 109.99, 108.71, 104.89, 100.01, 75.69, 70.40 (O**C**H_2_), 67.95 (O**C**H_2_), 67.92 (O**C**H_2_), 33.91, 33.87, 32.81, 32.79, 31.75, 30.82, 29.26, 29.19, 28.05, 27.99, 27.96, 27.74, 25.55, 25.46.

1.4. *(R)-5-((5-bromopentyl)oxy)-2-(8,8-dimethyl-3,4-dihydro-2H,8H-pyrano[2,3-f] chromen-3-yl)phenol (****3d****)*. Colorless oil, yield 15.3%. ^1^H NMR (500 MHz, Chloroform-*d*) *δ* 7.00 (d, *J* = 8.5 Hz, 1H, Ar**H**), 6.83 (d, *J* = 8.2 Hz, 1H, CH=C**H**), 6.65 (d, *J* = 9.9 Hz, 1H, Ar**H**), 6.46 (dt, *J* = 8.6, 2.1 Hz, 1H, Ar**H**), 6.40-6.32 (m, 2H, Ar**H**), 5.56 (dd, *J* = 9.9, 1.8 Hz, 1H, C**H**=CH), 4.38 (dt, *J* = 10.8, 2.3 Hz, 1H), 4.02 (td, *J* = 10.2, 1.8 Hz, 1H), 3.94-3.88 (m, 2H), 3.49 (td, *J* = 10.8, 10.3, 5.0 Hz, 1H), 3.44 (td, *J* = 6.8, 1.7 Hz, 2H), 2.99 (dd, *J* = 15.7, 10.9 Hz, 1H), 2.86 (dd, *J* = 15.7, 5.1 Hz, 1H), 1.93 (p, *J* = 7.1 Hz, 2H), 1.79 (p, *J* = 6.8 Hz, 2H), 1.64-1.61 (m, 2H), 1.43 (s, 3H), 1.41 (s, 3H). ^13^C NMR (126 MHz, Chloroform-*d*) *δ* 158.87 (OAr**C**), 154.43 (OAr**C**), 151.99 (OAr**C**), 149.89 (OAr**C**), 129.33, 129.08, 128.30, 119.97, 117.09, 114.53, 110.06, 108.84, 106.82, 102.76, 75.77, 70.16 (O**C**H_2_), 67.80 (O**C**H_2_), 33.72, 32.59, 31.87, 30.74, 28.53, 27.90, 27.68, 24.96.

1.5. *(R)-3-((5-bromopentyl)oxy)-4-(8,8-dimethyl-3,4-dihydro-2H,8H-pyrano[2,3-f] chromen-3-yl)phenol (****3e****)*. Colorless oil, yield 22.1%. ^1^H NMR (500 MHz, Chloroform-*d*) *δ* 6.95 (d, *J* = 8.2 Hz, 1H, Ar**H**), 6.82 (d, *J* = 8.2 Hz, 1H, CH=C**H**), 6.65 (d, *J* = 9.9 Hz, 1H, Ar**H**), 6.41 (d, *J* = 2.5 Hz, 1H, Ar**H**), 6.37 (d, *J* = 8.3 Hz, 2H, Ar**H**), 5.56 (d, *J* = 10.0 Hz, 1H, CH=C**H**), 4.81 (s, 1H), 4.33 (dt, *J* = 10.1, 2.8 Hz, 1H), 4.00 (d, *J* = 10.1 Hz, 1H), 3.95 (t, *J* = 6.4 Hz, 2H), 3.55 (tt, *J* = 9.7, 4.3 Hz, 1H), 3.42 (t, *J* = 6.7 Hz, 2H), 2.94 (dd, *J* = 15.7, 10.8 Hz, 1H), 2.83 (dd, *J* = 15.8, 5.3 Hz, 1H), 1.92 (p, *J* = 6.9 Hz, 2H), 1.82 (p, *J* = 6.7 Hz, 2H), 1.63 (d, *J* = 7.5 Hz, 2H), 1.43 (s, 3H), 1.42 (s, 3H). ^13^C NMR (126 MHz, Chloroform-*d*) *δ* 159.10 (OAr**C**), 157.73 (OAr**C**), 152.00 (OAr**C**), 150.01 (OAr**C**), 129.33, 129.00, 127.76, 122.14, 117.12, 114.67, 110.00, 108.72, 104.95, 99.98, 75.71, 70.39 (O**C**H_2_), 67.79 (O**C**H_2_), 33.73, 33.68, 32.62, 32.49, 31.71, 30.88, 28.61, 28.56, 27.96, 27.75, 25.04, 25.01.

2. Synthesis of **5a-5k**.

Synthesis of **5a-5i: 3a-3c** (0.336 mmol) and corresponding N-heterocyclic carbene **4a**-**4c** (3.360 mmol) were dissolved in acetonitrile and refluxed at 80 ℃ for 2-5 days until detecting the complete reaction through thin layer chromatography (TLC). The product was purified on silica gel (DCM / MeOH).

Synthesis of **5j-5k: 3d-3e** (0.153 mmol) and N-heterocyclic carbene **4a** (1.530 mmol) were dissolved in acetonitrile and refluxed at 80 ℃ for 2-5 days until detecting the complete reaction through TLC. The product was purified on silica gel (DCM / MeOH).

2.1. *(R)-3,3'-(((4-(8,8-dimethyl-3,4-dihydro-2H,8H-pyrano[2,3-f]chromen-3-yl) -1,3-phenylene)bis(oxy))-di-(butane-4,1-diyl))-di-(1-ethyl-1H-imidazol-3-ium)dibromide (****5a****)*. A white solid, yield 57.3 %. ^1^H NMR (500 MHz, DMSO-*d_6_*) *δ* 9.37 (s, 1H, NC**H**N), 9.33 (s, 1H, NC**H**N), 7.86 (dt, *J* = 6.0, 1.9 Hz, 2H, NC**H**=C**H**N), 7.80 (d, *J* = 1.6 Hz, 2H, NC**H**=C**H**N), 7.07 (d, *J* = 8.5 Hz, 1H, Ar**H**), 6.83 (d, *J* = 8.3 Hz, 1H, CH=C**H**), 6.57 (d, *J* = 2.4 Hz, 1H, Ar**H**), 6.50 (t, *J* = 8.7 Hz, 2H, Ar**H**), 6.30 (d, *J* = 8.2 Hz, 1H, Ar**H**), 5.66 (d, *J* = 9.9 Hz, 1H, C**H**=CH), 4.27 (td, *J* = 7.1, 4.5 Hz, 4H), 4.20 (ddd, *J* = 14.6, 9.3, 5.6 Hz, 5H), 4.03 (t, *J* = 6.6 Hz, 2H), 4.01-3.97 (m, 3H), 3.17 (d, *J* = 5.1 Hz, 1H), 2.90 (dd, *J* = 15.7, 10.7 Hz, 1H), 2.78-2.72 (m, 1H), 1.97 (t, *J* = 7.5 Hz, 4H), 1.72 (dq, *J* = 15.4, 8.7, 7.5 Hz, 4H), 1.45-1.40 (m, 6H, C**H**_3_), 1.35 (s, 3H, C**H**_3_), 1.34 (s, 3H, C**H**_3_). ^13^C NMR (126 MHz, DMSO-*d_6_*) *δ* 158.46 (OAr**C**), 156.97 (OAr**C**), 151.27 (OAr**C**), 149.19 (OAr**C**), 135.76 (N**C**HN), 135.72 (N**C**HN), 129.31, 127.64, 122.41, 122.29, 122.17, 121.20, 116.25, 114.45, 109.03, 108.25, 105.51, 99.83, 75.28, 69.60(O**C**H_2_), 67.04 (O**C**H_2_), 66.84 (O**C**H_2_), 48.57, 48.48, 44.23, 40.02, 39.86, 39.69, 39.52, 39.35, 39.19, 39.02, 30.70, 30.05, 27.40, 27.24, 26.32, 25.41, 15.01, 14.99.

2.2. *(R)-3,3'-(((4-(8,8-dimethyl-3,4-dihydro-2H,8H-pyrano[2,3-f]chromen-3-yl)- 1,3-phenylene)bis(oxy))-di-(butane-4,1-diyl))-di-(1-ethyl-4,5-di-(4-methoxyphenyl)-1H-imidazol-3-ium)dibromide (****5b****)*. A white solid, yield 63.4 %. ^1^H NMR (500 MHz, DMSO-*d_6_*) *δ* 9.52 (d, *J* = 14.2 Hz, 2H, NC**H**N), 7.98 (d, *J* = 1.8 Hz, 2H, NC**H**=C**H**N), 7.95 (d, *J* = 1.8 Hz, 2H, NC**H**=C**H**N), 7.33-7.30 (m, 5H, Ar**H**), 7.05 (d, *J* = 8.2 Hz, 1H, Ar**H**), 7.02-6.98 (m, 4H, Ar**H**), 6.95 (d, *J* = 8.4 Hz, 2H, Ar**H**), 6.83 (dd, *J* = 14.6, 8.4 Hz, 3H, CH=C**H**, Ar**H**), 6.48 (d, *J* = 9.9 Hz, 1H, Ar**H**), 6.41 (s, 1H, Ar**H**), 6.31 (d, *J* = 8.2 Hz, 1H, Ar**H**), 5.61 (d, *J* = 9.9 Hz, 1H, C**H**=CH), 4.20-4.12 (m, 5H), 4.07 (dd, *J* = 7.9, 5.7 Hz, 4H), 3.96 (t, *J* = 10.1 Hz, 1H), 3.85 (dt, *J* = 19.7, 6.2 Hz, 4H), 3.77 (d, *J* = 3.8 Hz, 6H, OC**H**_3_), 3.72 (s, 3H, OC**H**_3_), 3.66 (s, 3H, OC**H**_3_), 2.89 (dd, *J* = 15.9, 10.7 Hz, 1H), 2.74-2.68 (m, 1H), 1.77 (h, *J* = 7.0 Hz, 4H), 1.66 (p, *J* = 6.9 Hz, 4H), 1.32 (s, 3H, C**H**_3_), 1.30 (s, 3H, C**H**_3_), 1.28 (s, 3H, C**H**_3_), 1.25 (s, 3H, C**H**_3_). ^13^C NMR (126 MHz, DMSO-*d_6_*) *δ* 160.26 (Ar**C**OCH_3_), 160.24 (Ar**C**OCH_3_), 160.20 (Ar**C**OCH_3_), 160.17 (Ar**C**OCH_3_), 158.34 (OAr**C**), 156.86 (OAr**C**), 151.28 (OAr**C**), 149.17 (OAr**C**), 134.94 (2C, N**C**HN), 132.12, 132.06, 131.09, 131.04, 130.96, 129.32, 129.24, 127.55, 121.01, 117.18, 117.12, 117.08, 116.18, 114.42, 114.39, 114.29, 109.09, 108.31, 105.42, 99.52, 75.23, 69.56 (O**C**H_2_), 66.62 (2C, O**C**H_2_), 55.24 (2C, O**C**H_3_), 55.19 (O**C**H_3_), 55.14 (O**C**H_3_), 46.76, 42.56, 30.65, 30.02, 27.37, 27.11, 26.10, 25.76, 25.49, 25.25, 14.72, 14.66.

2.3. *(R)-3,3'-(((4-(8,8-dimethyl-3,4-dihydro-2H,8H-pyrano[2,3-f]chromen-3-yl)- 1,3-phenylene)bis(oxy))-di-(butane-4,1-diyl))-di-(1-ethyl-4,5-di-(4-fluorophenyl)-1H-imidazol-3-ium)dibromide (****5c****)*. A white solid, yield 40.9 %. ^1^H NMR (500 MHz, Chloroform-*d*) *δ* 10.77 (s, 1H, NC**H**N), 10.69 (s, 1H, NC**H**N), 7.36-7.30 (m, 5H, Ar**H**), 7.25 (t, *J* = 4.5 Hz, 3H, Ar**H**), 7.16-7.04 (m, 8H, Ar**H**), 6.97 (d, *J* = 8.4 Hz, 1H, Ar**H**), 6.83 (d, *J* = 8.2 Hz, 1H, CH=C**H**), 6.67-6.61 (m, 2H, Ar**H**), 6.38 (dd, *J* = 12.1, 8.3 Hz, 2H, Ar**H**), 5.55 (d, *J* = 9.9 Hz, 1H, C**H**=CH), 4.36 (dddd, *J* = 27.9, 18.3, 13.4, 5.1 Hz, 9H), 4.17 (s, 2H), 4.05 (t, *J* = 5.9 Hz, 2H), 3.96 (t, *J* = 10.4 Hz, 1H), 3.46 (td, *J* = 10.8, 5.3 Hz, 1H), 2.95 (dd, *J* = 15.7, 11.4 Hz, 1H), 2.83-2.76 (m, 1H), 2.05 (q, *J* = 7.3 Hz, 4H), 1.87 (s, 4H), 1.52 (t, *J* = 7.0 Hz, 6H, C**H**_3_), 1.43 (s, 3H, C**H**_3_), 1.39 (s, 3H, C**H**_3_). ^13^C NMR (126 MHz, Chloroform-*d*) *δ* 164.81 (2C, Ar**C**-F), 162.80 (2C, Ar**C**-F), 158.80 (OAr**C**), 157.42 (OAr**C**), 151.97 (OAr**C**), 149.86 (OAr**C**), 137.04 (N**C**HN), 136.97 (N**C**HN), 132.88, 132.81, 132.75, 132.72, 131.32, 131.28, 131.20, 129.28, 129.08, 127.58, 121.53, 121.03, 116.99, 116.91, 116.88, 116.82, 116.73, 116.70, 114.68, 109.92, 108.73, 106.41, 99.89, 75.67, 70.36 (O**C**H_2_), 67.23 (O**C**H_2_), 67.19 (O**C**H_2_), 47.79, 47.63, 43.71, 31.72, 30.87, 27.87, 27.64, 27.15, 26.95, 26.00, 25.89, 15.78, 15.74.

2.4. *(R)-3,3'-(((4-(8,8-dimethyl-3,4-dihydro-2H,8H-pyrano[2,3-f]chromen-3-yl)- 1,3-phenylene)bis(oxy))-di-(pentane-5,1-diyl))-di-(1-ethyl-1H-imidazol-3-ium)dibromide(****5d****)*. Colorless oil, yield 86.8 %. ^1^H NMR (500 MHz, Chloroform-*d*) *δ* 10.40 (d, *J* = 4.2 Hz, 2H, NC**H**N), 7.55 (d, *J* = 7.5 Hz, 2H, NC**H**=C**H**N), 7.41 (s, 1H, NCH=C**H**N), 7.30 (s, 1H, NC**H**=CHN), 6.95 (d, *J* = 8.4 Hz, 1H, Ar**H**), 6.81 (d, *J* = 8.2 Hz, 1H, C**H**=CH), 6.62 (d, *J* = 9.9 Hz, 1H, Ar**H**), 6.48 (d, *J* = 2.4 Hz, 1H, Ar**H**), 6.39 (dd, *J* = 8.4, 2.4 Hz, 1H, Ar**H**), 6.35 (d, *J* = 8.2 Hz, 1H, Ar**H**), 5.55 (d, *J* = 9.9 Hz, 1H, CH=C**H**), 4.59 (s, 2H), 4.44-4.31 (m, 8H, NC**H**_2_), 4.28 (dt, *J* = 10.6, 2.7 Hz, 1H), 4.00 (td, *J* = 6.5, 3.1 Hz, 2H), 3.97-3.92 (m, 2H), 3.44 (tt, *J* = 10.6, 4.3 Hz, 1H), 2.91 (dd, *J* = 15.7, 11.1 Hz, 1H), 2.81-2.74 (m, 1H), 2.02 (t, *J* = 7.8 Hz, 4H), 1.80 (q, *J* = 7.1 Hz, 2H), 1.62-1.56 (m, 5H), 1.55 (d, *J* = 7.5 Hz, 6H, C**H**_3_), 1.40 (s, 3H, C**H**_3_), 1.39 (s, 3H, C**H**_3_). ^13^C NMR (126 MHz, Chloroform-*d*) *δ* 159.02 (OAr**C**), 157.57 (OAr**C**), 151.95 (OAr**C**), 149.89 (OAr**C**), 136.71 (N**C**HN), 136.66 (N**C**HN), 129.39, 129.24, 127.65, 122.48, 122.35, 122.08, 121.83, 121.62, 116.95, 114.78, 109.91, 108.75, 105.56, 100.02, 75.74, 70.36 (O**C**H_2_), 67.70 (O**C**H_2_), 67.56 (O**C**H_2_), 50.05, 49.97, 45.38, 31.66, 30.77, 29.99, 29.89, 28.53, 28.44, 27.79, 23.01, 22.89, 15.79, 15.69.

2.5. *(R)-3,3'-(((4-(8,8-dimethyl-3,4-dihydro-2H,8H-pyrano[2,3-f]chromen-3-yl)- 1,3-phenylene)bis(oxy))-di-(pentane-5,1-diyl))-di-(1-ethyl-4,5-di-(4-methoxyphenyl)-1H-imidazol-3-ium)dibromide (****5e****)*. A white solid, yield 79.8 %. ^1^H NMR (500 MHz, Chloroform-*d*) *δ* 10.67 (s, 2H, NC**H**N), 7.12 (q, *J* = 7.6, 7.1 Hz, 8H, Ar**H**), 6.94 (d, *J* = 8.4 Hz, 1H, Ar**H**), 6.89 (t, *J* = 7.0 Hz, 6H, Ar**H**), 6.84 (d, *J* = 8.1 Hz, 2H, Ar**H**), 6.78 (d, *J* = 8.3 Hz, 1H, C**H**=CH), 6.59 (d, *J* = 9.9 Hz, 1H, Ar**H**), 6.47 (s, 1H, Ar**H**), 6.38 (d, *J* = 8.4 Hz, 1H, Ar**H**), 6.33 (d, *J* = 8.2 Hz, 1H, Ar**H**), 5.52 (d, *J* = 9.9 Hz, 1H, CH=C**H**), 4.27 (q, *J* = 6.9, 6.2 Hz, 8H), 3.93 (dt, *J* = 11.3, 6.2 Hz, 6H), 3.79 (d, *J* = 3.5 Hz, 9H, OC**H**_3_), 3.74 (s, 3H, OC**H**_3_), 3.45 (dq, *J* = 11.3, 6.5, 5.3 Hz, 1H), 2.90 (dd, *J* = 15.6, 11.0 Hz, 1H), 2.76 (dd, *J* = 15.9, 5.1 Hz, 1H), 1.87 (q, *J* = 7.1 Hz, 4H), 1.74 (dt, *J* = 14.7, 7.2 Hz, 4H), 1.45 (q, *J* = 7.1 Hz, 10H), 1.40 (s, 3H, C**H**_3_), 1.38 (s, 3H, C**H**_3_). ^13^C NMR (126 MHz, Chloroform-*d*) *δ* 160.98 (4C, Ar**C**OCH_3_), 159.07 (OAr**C**), 157.57 (OAr**C**), 151.89 (OAr**C**), 149.90 (OAr**C**), 136.56 (2C, N**C**HN), 131.87, 131.83, 131.70, 131.53, 129.30, 128.96, 127.57, 121.52, 117.04, 116.94, 116.89, 114.89, 114.85, 114.69, 109.89, 108.66, 105.63, 99.71, 75.64, 70.31 (O**C**H_2_), 67.57 (O**C**H_2_), 67.50 (O**C**H_2_), 55.50 (O**C**H_3_), 55.48 (2C, O**C**H_3_), 55.44 (O**C**H_3_), 47.81, 47.73, 43.39, 31.59, 30.74, 29.82, 29.61, 28.50, 28.43, 27.84, 27.72, 22.96, 22.88, 15.97, 15.93.

2.6. *(R)-3,3'-(((4-(8,8-dimethyl-3,4-dihydro-2H,8H-pyrano[2,3-f]chromen-3-yl)- 1,3-phenylene)bis(oxy))-di-(pentane-5,1-diyl))-di-(1-ethyl-4,5-di-(4-fluorophenyl)-1H-imidazol-3-ium) dibromide (****5f****).* A white solid, yield 44.1 %. ^1^H NMR (500 MHz, Chloroform-*d*) *δ* 10.68 (s, 2H, NC**H**N), 7.29 (t, *J* = 7.0 Hz, 8H, Ar**H**), 7.09 (qd, *J* = 8.4, 4.9 Hz, 8H, Ar**H**), 6.94 (d, *J* = 8.3 Hz, 1H, Ar**H**), 6.79 (d, *J* = 8.2 Hz, 1H, C**H**=CH), 6.58 (d, *J* = 9.9 Hz, 1H, Ar**H**), 6.52 (s, 1H, Ar**H**), 6.38 (d, *J* = 8.5 Hz, 1H, Ar**H**), 6.33 (d, *J* = 8.2 Hz, 1H, Ar**H**), 5.52 (d, *J* = 9.9 Hz, 1H, CH=C**H**), 4.28 (ddd, *J* = 26.1, 11.4, 6.2 Hz, 9H), 3.99 (q, *J* = 5.8 Hz, 2H), 3.96-3.90 (m, 3H), 3.45 (tt, *J* = 10.1, 4.2 Hz, 1H), 2.90 (dd, *J* = 15.7, 11.1 Hz, 1H), 2.76 (dd, *J* = 15.8, 5.1 Hz, 1H), 1.91 (h, *J* = 7.1 Hz, 4H), 1.74 (dq, *J* = 21.0, 6.8 Hz, 4H), 1.47 (p, *J* = 7.4, 6.6 Hz, 10H), 1.40 (s, 3H, C**H**_3_), 1.38 (s, 3H, C**H**_3_). ^13^C NMR (126 MHz, Chloroform-*d*) *δ* 164.81 (2C, Ar**C**-F), 162.80 (2C, Ar**C**-F), 159.06 (OAr**C**), 157.59 (OAr**C**), 151.92 (OAr**C**), 149.87 (OAr**C**), 137.13 (N**C**HN), 137.09 (N**C**HN), 132.79, 132.73, 131.29, 131.14, 129.31, 129.06, 127.60, 121.52, 120.87, 116.98, 116.85, 116.80, 114.67, 109.90, 108.71, 105.93, 99.75, 75.68, 70.33 (O**C**H_2_), 67.55 (O**C**H_2_), 67.46 (O**C**H_2_), 48.13, 48.03, 43.73, 31.64, 30.76, 29.79, 29.68, 29.50, 28.40, 28.36, 27.85, 27.69, 22.96, 22.88, 15.87, 15.84.

2.7. *(R)-3,3'-(((4-(8,8-dimethyl-3,4-dihydro-2H,8H-pyrano[2,3-f]chromen-3-yl)- 1,3-phenylene)bis(oxy))-di-(hexane-6,1-diyl))-di-(1-ethyl-1H-imidazol-3-ium)dibromide (****5g****).* Colorless oil, yield 61.2 %. ^1^H NMR (500 MHz, DMSO-*d_6_*) *δ* 9.26 (s, 1H, NC**H**N), 9.21 (s, 1H, NC**H**N), 7.82 (ddd, *J* = 7.9, 3.6, 1.9 Hz, 3H, NC**H**=C**H**N), 7.77 (t, *J* = 1.8 Hz, 1H, NC**H**=C**H**N), 7.06 (d, *J* = 8.4 Hz, 1H, Ar**H**), 6.84 (d, *J* = 8.3 Hz, 1H, C**H**=CH), 6.54-6.50 (m, 2H, Ar**H**), 6.47 (dd, *J* = 8.4, 2.4 Hz, 1H, Ar**H**), 6.30 (d, *J* = 8.2 Hz, 1H, Ar**H**), 5.65 (d, *J* = 10.0 Hz, 1H, CH=C**H**), 4.19 (q, *J* = 7.2 Hz, 6H), 4.15-4.10 (m, 3H), 4.03-3.96 (m, 3H), 3.94 (t, *J* = 6.4 Hz, 2H), 2.92 (dd, *J* = 15.7, 10.8 Hz, 1H), 2.77-2.72 (m, 1H), 1.82 (dt, *J* = 15.3, 7.7 Hz, 4H), 1.71 (tt, *J* = 14.6, 6.4 Hz, 4H), 1.42 (td, *J* = 7.3, 5.3 Hz, 10H), 1.35-1.30 (m, 10H). ^13^C NMR (126 MHz, DMSO-*d_6_*) *δ* 158.66 (OAr**C**), 157.13 (OAr**C**), 151.25 (OAr**C**), 149.18 (OAr**C**), 135.70 (N**C**HN), 135.65 (N**C**HN), 129.30, 128.65, 127.62, 122.40, 122.36, 122.12, 120.96, 116.24, 114.49, 109.01, 108.23, 105.31, 99.65, 75.26, 69.60 (O**C**H_2_), 67.55 (O**C**H_2_), 67.30 (O**C**H_2_), 48.77, 48.73, 44.20, 30.88, 30.05, 29.28, 28.46, 28.42, 27.39, 27.24, 25.27, 25.22, 24.97, 24.92, 15.05, 15.03.

2.8. *(R)-3,3'-(((4-(8,8-dimethyl-3,4-dihydro-2H,8H-pyrano[2,3-f]chromen-3-yl)- 1,3-phenylene)bis(oxy))-di-(hexane-6,1-diyl))-di-(1-ethyl-4,5-di-(4-methoxyphenyl)-1H-imidazol-3-ium)dibromide (****5h****)*. A white solid, yield 82.2 %. ^1^H NMR (500 MHz, Chloroform-*d*) *δ* 10.73 (d, *J* = 11.4 Hz, 2H, NC**H**N), 7.15-7.08 (m, 8H, Ar**H**), 6.96 (d, *J* = 8.4 Hz, 1H, Ar**H**), 6.92-6.86 (m, 8H, Ar**H**), 6.81 (d, *J* = 8.3 Hz, 1H, C**H**=CH), 6.62 (d, *J* = 9.9 Hz, 1H, Ar**H**), 6.44 (d, *J* = 2.3 Hz, 1H, Ar**H**), 6.39 (dd, *J* = 8.4, 2.1 Hz, 1H, Ar**H**), 6.33 (d, *J* = 8.2 Hz, 1H, Ar**H**), 5.54 (d, *J* = 9.9 Hz, 1H, CH=C**H**), 4.30-4.20 (m, 9H), 3.99-3.86 (m, 6H), 3.80 (d, *J* = 5.4 Hz, 9H, OC**H**_3_), 3.76 (s, 2H), 3.49 (tt, *J* = 10.2, 4.8 Hz, 1H), 2.92 (dd, *J* = 15.7, 11.0 Hz, 1H), 2.82-2.76 (m, 1H), 1.80 (d, *J* = 7.3 Hz, 3H), 1.72-1.67 (m, 5H), 1.46 (dt, *J* = 7.1, 3.8 Hz, 7H), 1.39 (d, *J* = 10.5 Hz, 9H), 1.34 (s, 1H), 1.32 (s, 1H), 1.29 (s, 1H), 1.28 (s, 1H). ^13^C NMR (126 MHz, Chloroform-*d*) *δ* 161.01 (4C, Ar**C**OCH_3_), 159.16 (OAr**C**), 157.70 (OAr**C**), 151.90 (OAr**C**), 149.95 (OAr**C**), 136.55 (2C, N**C**HN), 131.89, 131.70, 131.54, 129.32, 128.96, 127.60, 121.67, 117.07, 117.02, 116.99, 114.87, 114.84, 109.91, 108.62, 105.32, 99.84, 75.64, 70.36 (O**C**H_2_), 67.83 (2C, O**C**H_2_), 55.49 (2C, O**C**H_3_), 55.47 (2C, O**C**H_3_), 47.79, 47.74, 43.35, 31.68, 30.81, 30.31, 30.15, 28.99, 28.80, 27.84, 27.73, 26.07, 25.88, 25.45, 25.36, 15.96.

2.9. *(R)-3,3'-(((4-(8,8-dimethyl-3,4-dihydro-2H,8H-pyrano[2,3-f]chromen-3-yl)- 1,3-phenylene)bis(oxy))-di-(hexane-6,1-diyl))-di-(1-ethyl-4,5-di-(4-fluorophenyl)-1H-imidazol-3-ium) dibromide (****5i****).* A white solid, yield 28.6 %. ^1^H NMR (500 MHz, DMSO-*d_6_*) *δ* 9.68 (s, 1H, NC**H**N), 9.63 (s, 1H, NC**H**N), 7.47 (ddq, *J* = 20.2, 8.3, 5.4, 5.0 Hz, 8H, Ar**H**), 7.34-7.24 (m, 8H, Ar**H**), 7.05 (d, *J* = 8.4 Hz, 1H, Ar**H**), 6.82 (d, *J* = 8.3 Hz, 1H, C**H**=CH), 6.53-6.48 (m, 2H, Ar**H**), 6.47-6.41 (m, 1H, Ar**H**), 6.29 (d, J = 8.2 Hz, 1H, Ar**H**), 5.64 (d, *J* = 9.9 Hz, 1H, CH=C**H**), 4.19 (d, *J* = 10.5 Hz, 1H), 4.08 (tt, *J* = 15.8, 7.2 Hz, 8H), 3.99 (t, *J* = 10.1 Hz, 1H), 3.89 (dt, *J* = 19.4, 6.5 Hz, 4H), 3.16 (s, 1H), 2.90 (dd, *J* = 15.8, 10.7 Hz, 1H), 2.72 (dd, *J* = 15.8, 5.1 Hz, 1H), 1.63 (dh, *J* = 14.7, 7.5, 7.1 Hz, 8H), 1.34-1.25 (m, 20H). ^13^C NMR (126 MHz, DMSO-*d_6_*) *δ* 163.92 (2C, Ar**C**-F), 161.95 (2C, Ar**C**-F), 158.65 (OAr**C**), 157.10 (OAr**C**), 151.27 (OAr**C**), 149.18 (OAr**C**), 135.48 (2C, N**C**HN), 133.31, 133.24, 133.20, 130.56, 130.52, 129.34, 129.32, 127.63, 121.56, 121.53, 120.93, 116.31, 116.29, 116.27, 116.13, 114.50, 109.04, 108.26, 105.39, 99.58, 75.28, 67.45 (O**C**H_2_), 67.22 (O**C**H_2_), 62.79 (O**C**H_2_), 47.06, 47.00, 42.73, 31.30, 30.84, 30.07, 29.02, 28.76, 28.33, 27.34, 27.24, 25.13, 25.08, 24.78, 24.76, 14.70, 14.67.

2.10. *(R)-3-(5-(4-(8,8-dimethyl-3,4-dihydro-2H,8H-pyrano[2,3-f]chromen-3-yl)-3- hydroxyphenoxy)pentyl)-1-ethyl-1H-imidazol-3-ium bromide(****5j****).* A white solid, yield 88.8%. ^1^H NMR (500 MHz, Chloroform-*d*) *δ* 10.06 (s, 1H, NC**H**N), 7.37 (dd, *J* = 14.8, 2.0 Hz, 2H, NC**H**=C**H**N), 6.99 (d, *J* = 2.4 Hz, 1H, Ar**H**), 6.88 (d, *J* = 8.3 Hz, 1H, CH=C**H**), 6.78 (d, *J* = 8.2 Hz, 1H, Ar**H**), 6.60 (d, *J* = 9.8 Hz, 1H, Ar**H**), 6.33 (d, *J* = 8.2 Hz, 1H, Ar**H**), 6.28 (dd, *J* = 8.4, 2.4 Hz, 1H, Ar**H**), 5.54 (d, *J* = 9.9 Hz, 1H, CH=C**H**), 4.30 (p, *J* = 7.4 Hz, 5H), 3.96 (t, *J* = 10.1 Hz, 1H), 3.86 (t, *J* = 6.3 Hz, 2H), 3.43-3.35 (m, 1H), 2.93 (dd, *J* = 15.7, 10.8 Hz, 1H), 2.76 (dd, *J* = 15.7, 5.1 Hz, 1H), 1.96 (p, *J* = 7.6 Hz, 2H), 1.73 (s, 2H), 1.50 (t, *J* = 7.3 Hz, 3H), 1.44 (t, *J* = 7.6 Hz, 2H), 1.41 (s, 3H), 1.40 (s, 3H). ^13^C NMR (126 MHz, Chloroform-*d*) *δ* 158.44 (OAr**C**), 156.20 (OAr**C**), 151.92 (OAr**C**), 149.95 (OAr**C**), 136.43 (N**C**HN), 129.40, 129.14, 127.74, 122.46, 121.83, 120.06, 117.06, 114.82, 109.92, 108.71, 105.94, 103.18, 75.73, 70.22 (O**C**H_2_), 67.62 (O**C**H_2_), 50.02, 45.48, 31.83, 30.50, 29.62, 28.01, 27.84, 27.76, 22.77, 15.60.

2.11. *(R)-3-(5-(2-(8,8-dimethyl-3,4-dihydro-2H,8H-pyrano[2,3-f]chromen-3-yl)-5- hydroxyphenoxy)pentyl)-1-ethyl-1H-imidazol-3-ium bromide (****5k****).* A white solid, yield 78.9%. ^1^H NMR (500 MHz, Chloroform-*d*) *δ* 7.41 (d, *J* = 20.0 Hz, 2H, NC**H**=C**H**N), 6.96 (d, *J* = 2.6 Hz, 1H, Ar**H**), 6.87 (d, *J* = 8.6 Hz, 1H, CH=C**H**), 6.77 (d, *J* = 8.2 Hz, 1H, Ar**H**), 6.59 (d, *J* = 9.9 Hz, 1H, Ar**H**), 6.32 (d, *J* = 8.2 Hz, 1H, Ar**H**), 6.27 (dd, *J* = 8.4, 2.5 Hz, 1H, Ar**H**), 5.53 (d, *J* = 9.8 Hz, 1H, CH=C**H**), 4.28 (p, *J* = 7.0, 6.0 Hz, 5H), 3.94 (t, *J* = 10.2 Hz, 1H), 3.83 (t, *J* = 6.3 Hz, 2H), 3.37 (tt, *J* = 9.3, 4.0 Hz, 1H), 2.92 (dd, *J* = 15.7, 10.8 Hz, 1H), 2.74 (dd, *J* = 15.9, 5.0 Hz, 1H), 1.94 (p, *J* = 7.4 Hz, 2H), 1.69 (t, *J* = 7.0 Hz, 2H), 1.49 (t, *J* = 7.4 Hz, 3H), 1.43 (d, *J* = 7.9 Hz, 2H), 1.40 (s, 3H), 1.39 (s, 3H). ^13^C NMR (126 MHz, Chloroform-*d*) *δ* 158.46 (OAr**C**), 156.16 (OAr**C**), 151.92 (OAr**C**), 149.94 (OAr**C**), 136.34 (N**C**HN), 129.39, 129.14, 127.73, 122.45, 121.81, 120.02, 117.05, 114.83, 109.92, 108.72, 106.00, 103.11, 75.73, 70.22, 67.62, 50.03, 45.47, 31.83, 30.51, 29.61, 28.04, 27.83, 27.76, 22.77, 15.56.

3. Synthesis of **6a-6k**.

Synthesis of **6a-6i:** The ligands **5a-5i** (0.127 mmol) were initially dissolved in dichloromethane, followed by the addition of silver oxide (0.203 mmol). Then the mixture was stirred overnight under N_2_ in the darkness. After adding (CH_3_)_2_SAuCl (0.267 mmol), the solution was kept in the same conditions for 12 h. Subsequently, sodium bromide (1.905 mmol) was added under the same conditions for 12 h. The complexes **6a**-**6i** were separated by chromatography column (PE / EA).

Synthesis of **6j-6k:** The ligands **5j-5k** (0.113 mmol) were initially dissolved in dichloromethane, followed by the addition of silver oxide (0.113 mmol). Then the mixture was stirred overnight under N_2_ in the darkness. After adding (CH_3_)_2_SAuCl (0.124 mmol), the mixture was kept in the same conditions for 12 h. Subsequently, sodium bromide (1.695 mmol) was added under the same conditions for another 12 h. The complexes **6j**-**6k** were separated by chromatography column (PE / EA).

3.1.*[Dibromo-(R)-3,3'-(((4-(8,8-dimethyl-3,4-dihydro-2H,8H-pyrano[2,3-f]chromen-3-yl)-1,3-phenylene)bis(oxy))-di-(butane-4,1-diyl))-di-(1-ethyl-imidazol-2-ylidene)]-di-gold(I) (****6a****).* A yellow solid，yield 30.8 %. ^1^H NMR (500 MHz, Chloroform-*d*) *δ* 7.02 (d, *J* = 1.9 Hz, 1H, NC**H**=C**H**N), 7.00-6.96 (m, 2H, NC**H**=C**H**N), 6.90 (d, *J* = 2.0 Hz, 1H, NC**H**=C**H**N), 6.81 (d, *J* = 6.1 Hz, 2H, C**H**=CH, Ar**H**), 6.63 (d, *J* = 9.8 Hz, 1H, Ar**H**), 6.48 (s, 1H, Ar**H**), 6.44 (dd, *J* = 8.4, 2.4 Hz, 1H, Ar**H**), 6.36 (d, *J* = 8.1 Hz, 1H, Ar**H**), 5.56 (d, *J* = 9.8 Hz, 1H, CH=C**H**), 4.31 (d, *J* = 10.2 Hz, 1H), 4.24 (dt, *J* = 12.6, 7.2 Hz, 6H), 4.19-4.15 (m, 2H), 4.02 (dt, *J* = 11.7, 5.7 Hz, 4H), 3.96 (t, *J* = 10.3 Hz, 1H), 3.47 (td, *J* = 10.4, 4.8 Hz, 1H), 2.95 (dd, *J* = 15.7, 11.3 Hz, 1H), 2.82-2.75 (m, 1H), 2.05 (dq, *J* = 13.4, 7.7 Hz, 4H), 1.85-1.77 (m, 4H), 1.45 (dd, *J* = 14.2, 7.2 Hz, 6H), 1.42 (s, 3H, C**H**_3_), 1.41 (s, 3H, C**H**_3_). ^13^C NMR (126 MHz, DMSO-*d_6_*) *δ* 170.93 (**C**-Au), 170.89 (**C**-Au), 158.46 (OAr**C**), 156.95 (OAr**C**), 151.23 (OAr**C**), 149.23 (OAr**C**), 129.34, 129.20, 127.55, 121.52, 121.43, 121.14, 121.12, 120.99, 116.36, 114.46, 109.02, 108.19, 105.52, 99.72, 75.25, 69.57 (O**C**H_2_), 66.97 (O**C**H_2_), 66.82 (O**C**H_2_), 50.07, 49.96, 45.63, 45.60, 30.95, 30.72, 29.97, 27.41, 27.25, 27.19, 25.59, 25.55, 16.44, 16.40. ESI-MS (+) [m/z]: 1099.28 [M-Br] ^+^. Purity > 95 % by HPLC.

3.2.*[Dibromo(R)-3,3'-(((4-(8,8-dimethyl-3,4-dihydro-2H,8H-pyrano[2,3-f]chromen- 3-yl)-1,3-phenylene)bis(oxy))-di-(butane-4,1-diyl))-di-(1-ethyl-4,5-di-(4-methoxyphenyl)-imidazol-2-ylidene)]-di-gold(I) (****6b****).* A yellow solid，yield 57.5 %. ^1^H NMR (500 MHz, Chloroform-*d*) *δ* 7.11-7.06 (m, 6H, Ar**H**), 7.01 (d, *J* = 8.7 Hz, 2H, Ar**H**), 6.95 (d, *J* = 9.0 Hz, 1H, Ar**H**), 6.86 (dd, *J* = 8.8, 2.9 Hz, 4H, Ar**H**), 6.81 (dd, *J* = 8.6, 6.7 Hz, 3H, C**H**=CH, Ar**H**), 6.69 (d, *J* = 8.7 Hz, 2H, Ar**H**), 6.60 (d, *J* = 9.9 Hz, 1H, Ar**H**), 6.36-6.33 (m, 3H, Ar**H**), 5.49 (d, *J* = 9.9 Hz, 1H, CH=C**H**), 4.30 (ddd, *J* = 10.3, 3.5, 1.9 Hz, 1H), 4.23-4.13 (m, 8H), 3.94 (t, *J* = 10.2 Hz, 1H), 3.88 (q, *J* = 5.9 Hz, 2H), 3.83 (t, *J* = 6.3 Hz, 2H), 3.80 (d, *J* = 2.7 Hz, 6H, OC**H**_3_), 3.74 (d, *J* = 1.9 Hz, 3H, OC**H**_3_), 3.66 (d, *J* = 3.5 Hz, 3H, OC**H**_3_), 3.45 (tt, *J* = 10.5, 4.1 Hz, 1H), 2.92 (dd, *J* = 15.8, 11.0 Hz, 1H), 2.80 (dd, *J* = 15.8, 4.8 Hz, 1H), 1.89 (p, *J* = 7.4 Hz, 4H), 1.70 (q, *J* = 7.0 Hz, 4H), 1.40 (s, 3H, C**H**_3_), 1.32 (s, 3H, C**H**_3_), 1.31-1.27 (m, 6H, C**H**_3_). ^13^C NMR (126 MHz, Chloroform-*d*) *δ* 172.68 (**C**-Au), 172.57 (**C**-Au), 160.28 (2C, Ar**C**OCH_3_), 160.23 (2C, Ar**C**OCH_3_), 158.92 (OAr**C**), 157.44 (OAr**C**), 151.99 (OAr**C**), 149.98 (OAr**C**), 131.94, 131.13, 131.05, 130.89, 130.87, 129.32, 128.99, 127.60, 121.61, 119.90, 119.86, 119.67, 117.09, 114.69, 114.46, 114.44, 114.42, 110.02, 108.78, 105.59, 99.55, 75.67, 70.37 (O**C**H_2_), 67.20 (O**C**H_2_), 67.06 (O**C**H_2_), 55.41 (O**C**H_3_), 55.39 (2C, O**C**H_3_), 55.37 (O**C**H_3_), 48.62, 44.31, 31.57, 30.82, 29.20, 28.20, 27.92, 27.68, 26.38, 26.15, 17.08, 17.06. ESI-MS (+) [m/z]:1523.90 [M-Br] ^+^. Purity > 95 % by HPLC.

3.3.*[Dibromo(R)-3,3'-(((4-(8,8-dimethyl-3,4-dihydro-2H,8H-pyrano[2,3-f]chromen- 3-yl)-1,3-phenylene)bis(oxy))-di-(butane-4,1-diyl))-di-(1-ethyl-4,5-di-(4-fluorophenyl)-imidazol-2-ylidene)]-di-gold(I) (****6c****).* A yellow solid，yield 64.7 %. ^1^H NMR (500 MHz, DMSO-*d_6_*) *δ* 7.45-7.38 (m, 6H, Ar**H**), 7.32 (ddd, *J* = 8.6, 5.3, 2.6 Hz, 2H, Ar**H**), 7.26 (t, *J* = 8.8 Hz, 4H, Ar**H**), 7.18 (t, *J* = 8.8 Hz, 2H, Ar**H**), 7.05-6.99 (m, 3H, Ar**H**), 6.85 (d, *J* = 8.3 Hz, 1H, C**H**=CH), 6.50 (d, *J* = 9.9 Hz, 1H, Ar**H**), 6.38 (d, *J* = 7.9 Hz, 2H, Ar**H**), 6.30 (d, *J* = 8.2 Hz, 1H, Ar**H**), 5.61 (d, *J* = 9.9 Hz, 1H, CH=C**H**), 4.17 (td, *J* = 13.0, 4.3 Hz, 5H), 4.08 (p, *J* = 6.9 Hz, 4H), 3.96 (t, *J* = 10.1 Hz, 1H), 3.87 (s, 2H), 3.80 (t, *J* = 6.2 Hz, 2H), 2.88 (dd, *J* = 15.8, 10.6 Hz, 1H), 2.74 (dd, *J* = 15.5, 5.1 Hz, 1H), 1.75 (q, *J* = 8.0 Hz, 4H), 1.65-1.57 (m, 4H), 1.33 (s, 3H, C**H**_3_), 1.24 (s, 3H, C**H**_3_), 1.20 (dt, *J* = 9.4, 7.2 Hz, 6H, C**H**_3_). ^13^C NMR (126 MHz, DMSO-*d_6_*) *δ* 171.59 (**C**-Au), 171.54 (**C**-Au), 161.53 (Ar**C**-F), 161.52 (Ar**C**-F), 161.46 (Ar**C**-F), 161.39 (Ar**C**-F), 158.30 (OAr**C**), 156.81 (OAr**C**), 151.26 (OAr**C**), 149.21 (OAr**C**), 133.14, 133.07, 133.01, 132.95, 130.17, 130.07, 129.22, 123.61, 120.95, 116.24, 116.00, 115.92, 115.82, 115.75, 115.63, 114.47, 109.09, 108.28, 105.50, 99.42, 75.21, 69.56 (O**C**H_2_), 68.26 (O**C**H_2_), 66.51 (O**C**H_2_), 48.40, 43.93, 30.71, 30.00, 27.40, 27.07, 26.89, 25.78, 25.46, 16.39, 16.34. ESI-MS (+) [m/z]: 1476.89 [M-Br+H] ^+^. Purity > 95 % by HPLC.

3.4.*[Dibromo(R)-3,3'-(((4-(8,8-dimethyl-3,4-dihydro-2H,8H-pyrano[2,3-f]chromen- 3-yl)-1,3-phenylene)bis(oxy))-di-(pentane-5,1-diyl))-di-(1-ethyl-imidazol-2-ylidene)]-di-gold(I) (****6d****).* A yellowish solid, yield 54.7 %. ^1^H NMR (500 MHz, Chloroform-*d*) *δ* 6.99-6.95 (m, 3H, NC**H**=C**H**N), 6.87 (d, *J* = 1.8 Hz, 1H, NC**H**=C**H**N), 6.83 (d, *J* = 8.2 Hz, 1H, C**H**=CH), 6.77 (d, *J* = 1.8 Hz, 1H, Ar**H**), 6.64 (d, *J* = 9.9 Hz, 1H, Ar**H**), 6.46 (d, *J* = 2.1 Hz, 1H, Ar**H**), 6.43 (dd, *J* = 8.4, 2.1 Hz, 1H, Ar**H**), 6.36 (d, *J* = 8.2 Hz, 1H, Ar**H**), 5.56 (d, *J* = 9.9 Hz, 1H, CH=C**H**), 4.30 (dt, *J* = 10.2, 2.9 Hz, 1H), 4.22 (q, *J* = 6.9 Hz, 3H), 4.16 (q, *J* = 8.1, 7.2 Hz, 5H), 3.99-3.92 (m, 5H), 3.47 (dq, *J* = 10.6, 5.3, 4.4 Hz, 1H), 2.94 (dd, *J* = 15.6, 11.4 Hz, 1H), 2.79 (dd, *J* = 15.7, 3.6 Hz, 1H), 1.93 (p, *J* = 6.8 Hz, 4H), 1.86-1.79 (m, 4H), 1.52 (dt, *J* = 13.8, 7.6 Hz, 4H), 1.46 (dd, *J* = 14.0, 6.7 Hz, 6H, C**H**_3_), 1.42 (s, 3H, C**H**_3_), 1.40 (s, 3H, C**H**_3_). ^13^C NMR (126 MHz, Chloroform-*d*) *δ* 173.71 (**C**-Au), 173.55 (**C**-Au), 159.10 (OAr**C**), 157.63 (OAr**C**), 151.99 (OAr**C**), 149.94 (OAr**C**), 129.39, 129.18, 127.66, 121.67, 120.62, 120.58, 119.95, 119.89, 117.00, 114.80, 109.94, 108.75, 105.27, 99.85, 75.74, 70.43 (O**C**H_2_), 67.71 (O**C**H_2_), 67.55 (O**C**H_2_), 51.39, 51.17, 46.56, 46.49, 31.63, 30.89, 30.82, 30.78, 28.79, 28.71, 27.86, 27.82, 23.22, 23.20, 16.66, 16.59. ESI-MS (+) [m/z]:555.44 [M-2Br+MeOH] ^2+^ . Purity > 95 % by HPLC.

3.5.*[Dibromo(R)-3,3'-(((4-(8,8-dimethyl-3,4-dihydro-2H,8H-pyrano[2,3-f]chromen- 3-yl)-1,3-phenylene)bis(oxy))-di-(pentane-5,1-diyl))-di-(1-ethyl-4,5-di-(4-methoxyphenyl)-imidazol-2-ylidene)]-di-gold(I) (****6e****).* A yellow solid, yield 52.4 %. ^1^H NMR (500 MHz, DMSO-*d_6_*) *δ* 7.27 (d, *J* = 8.7 Hz, 3H, Ar**H**), 7.25-7.20 (m, 5H, Ar**H**), 7.03 (d, *J* = 8.5 Hz, 1H, Ar**H**), 6.94 (dd, *J* = 8.6, 5.8 Hz, 4H, Ar**H**), 6.90 (d, *J* = 8.8 Hz, 2H, Ar**H**), 6.86-6.81 (m, 3H, C**H**=CH, Ar**H**), 6.51 (d, *J* = 9.9 Hz, 1H, Ar**H**), 6.47 (d, *J* = 2.4 Hz, 1H, Ar**H**), 6.42 (dd, *J* = 8.5, 2.4 Hz, 1H, Ar**H**), 6.29 (d, *J* = 8.2 Hz, 1H, Ar**H**), 5.63 (d, *J* = 9.9 Hz, 1H, CH=C**H**), 4.18 (d, *J* = 10.0 Hz, 1H), 4.07 (ddd, *J* = 20.2, 10.7, 5.8 Hz, 8H), 3.96 (t, *J* = 10.0 Hz, 1H), 3.84 (dt, *J* = 13.4, 6.3 Hz, 4H), 3.74 (s, 6H, OC**H**_3_), 3.70 (s, 3H, OC**H**_3_), 3.66 (s, 3H, OC**H**_3_), 3.32 (s, 1H), 2.88 (dd, *J* = 15.9, 10.6 Hz, 1H), 2.73 (dd, *J* = 15.8, 5.0 Hz, 1H), 1.59 (dq, *J* = 21.4, 7.3 Hz, 8H), 1.34 (s, 3H, C**H**_3_), 1.32 (s, 3H, C**H**_3_), 1.31-1.22 (m, 10H). ^13^C NMR (126 MHz, DMSO-*d_6_*) *δ* 170.85 (**C**-Au), 170.78 (**C**-Au), 159.71 (2C, Ar**C**OCH_3_), 159.67 (Ar**C**OCH_3_), 159.65 (Ar**C**OCH_3_), 158.59 (OAr**C**), 157.05 (OAr**C**), 151.24 (OAr**C**), 149.22 (OAr**C**), 132.07, 132.02, 130.67, 130.63, 130.49, 130.47, 129.31, 129.18, 127.55, 120.92, 119.45, 119.42, 119.39, 119.36, 116.37, 114.44, 114.17, 114.10, 114.05, 109.05, 108.23, 105.32, 99.53, 75.25, 69.54 (O**C**H_2_), 67.10 (O**C**H_2_), 66.85 (O**C**H_2_), 55.13 (2C, O**C**H_3_), 55.09 (O**C**H_3_), 55.03 (O**C**H_3_), 48.43, 48.35, 43.75, 30.83, 29.96, 29.75, 29.58, 27.80, 27.38, 27.26, 22.22, 22.16, 16.48, 16.45. ESI-MS (+) [m/z]: 1551.76 [M- Br]^+^. Purity > 95 % by HPLC.

3.6.*[Dibromo(R)-3,3'-(((4-(8,8-dimethyl-3,4-dihydro-2H,8H-pyrano[2,3-f]chromen- 3-yl)-1,3-phenylene)bis(oxy))-di-(pentane-5,1-diyl))-di-(1-ethyl-4,5-di-(4-fluorophenyl)-imidazol-2-ylidene)]-di-gold(I) (****6f****).* A yellow solid, yield 30.8 %. ^1^H NMR (500 MHz, DMSO-*d_6_*) *δ* 7.45-7.41 (m, 4H, Ar**H**), 7.40-7.37 (m, 4H, Ar**H**), 7.26 (dt, *J* = 8.5, 4.3 Hz, 4H, Ar**H**), 7.20 (t, *J* = 8.8 Hz, 2H, Ar**H**), 7.15 (t, *J* = 8.8 Hz, 2H, Ar**H**), 7.03 (d, *J* = 8.5 Hz, 1H, Ar**H**), 6.82 (d, *J* = 8.3 Hz, 1H, C**H**=CH), 6.50 (d, *J* = 9.9 Hz, 1H, Ar**H**), 6.46 (d, *J* = 2.4 Hz, 1H, Ar**H**), 6.42 (dd, *J* = 8.5, 2.3 Hz, 1H, Ar**H**), 6.29 (d, *J* = 8.2 Hz, 1H, Ar**H**), 5.63 (d, *J* = 9.9 Hz, 1H, CH=C**H**), 4.19-4.16 (m, 1H), 4.08 (ddd, *J* = 12.1, 9.3, 6.1 Hz, 8H), 3.96 (t, *J* = 10.0 Hz, 1H), 3.88-3.82 (m, 4H), 3.31 (s, 1H), 2.87 (dd, *J* = 15.8, 10.6 Hz, 1H), 2.73 (dd, *J* = 15.4, 5.1 Hz, 1H), 1.64 (t, *J* = 6.8 Hz, 4H), 1.60-1.53 (m, 4H), 1.33 (s, 3H, C**H**_3_), 1.32 (s, 3H, C**H**_3_), 1.28-1.21 (m, 10H). ^13^C NMR (126 MHz, DMSO-*d_6_*) *δ* 171.57 (**C**-Au), 171.49 (**C**-Au), 163.50 (2C, Ar**C**-F), 161.53 (2C, Ar**C**-F), 158.57 (OAr**C**), 157.02 (OAr**C**), 151.24 (OAr**C**), 149.21 (OAr**C**), 133.08, 130.22, 130.18, 130.06, 130.04, 129.31, 129.18, 127.57, 123.67, 123.64, 120.94, 116.35, 116.01, 115.95, 115.84, 115.77, 114.45, 109.04, 108.23, 105.34, 99.54, 75.25, 69.53 (O**C**H_2_), 67.11 (O**C**H_2_), 66.85 (O**C**H_2_), 48.56, 43.93, 30.81, 29.97, 29.77, 29.63, 27.78, 27.39, 27.23, 22.22, 22.15, 16.41, 16.38.ESI-MS (+) [m/z]: 1503.97 [M-Br]^+^. Purity > 95 % by HPLC.

3.7.*[Dibromo(R)-3,3'-(((4-(8,8-dimethyl-3,4-dihydro-2H,8H-pyrano[2,3-f]chromen- 3-yl)-1,3-phenylene)bis(oxy))-di-(hexane-6,1-diyl))-di-(1-ethyl-imidazol-2-ylidene)]-di-gold(I) (****6g****).* A yellow solid, yield 60.8 %. ^1^H NMR (500 MHz, DMSO-*d_6_*) *δ* 7.52-7.47 (m, 3H, NC**H**=C**H**N), 7.45 (d, *J* = 2.0 Hz, 1H, NC**H**=C**H**N), 7.04 (d, *J* = 8.4 Hz, 1H, Ar**H**), 6.83 (d, *J* = 8.3 Hz, 1H, C**H**=CH), 6.54-6.49 (m, 2H, Ar**H**), 6.45 (dd, *J* = 8.5, 2.3 Hz, 1H, Ar**H**), 6.28 (d, *J* = 8.2 Hz, 1H, Ar**H**), 5.62 (d, *J* = 9.9 Hz, 1H, CH=C**H**), 4.25-4.21 (m, 1H), 4.12 (dd, *J* = 8.5, 5.6 Hz, 6H), 4.06 (t, *J* = 7.1 Hz, 2H), 4.00-3.95 (m, 3H), 3.93 (t, *J* = 6.5 Hz, 2H), 3.41-3.36 (m, 1H), 2.90 (dd, *J* = 15.7, 10.9 Hz, 1H), 2.74 (dd, *J* = 15.1, 5.0 Hz, 1H), 1.81 (dt, *J* = 15.2, 7.4 Hz, 4H), 1.70 (dt, *J* = 15.6, 7.3 Hz, 4H), 1.45 (t, *J* = 7.4 Hz, 4H), 1.35 (ddd, *J* = 18.2, 9.1, 6.1 Hz, 16H). ^13^C NMR (126 MHz, Chloroform-*d*) *δ* 173.79 (**C**-Au), 173.66 (**C**-Au), 159.20 (OAr**C**), 157.77 (OAr**C**), 151.96 (OAr**C**), 149.98 (OAr**C**), 129.37, 129.04, 127.69, 121.72, 120.57, 120.52, 119.92, 119.85, 117.07, 114.80, 109.95, 108.70, 105.24, 99.89, 75.70, 70.43 (O**C**H_2_), 67.97 (O**C**H_2_), 67.81 (O**C**H_2_), 51.34, 51.22, 46.55, 46.52, 31.84, 31.72, 31.10, 31.00, 29.18, 29.08, 27.91, 27.78, 26.16, 26.11, 26.06, 25.83, 25.72, 16.63, 16.61. ESI-MS (+) [m/z]: 1155.85 [M-Br] ^+^. Purity > 95 % by HPLC.

3.8.*[Dibromo(R)-3,3'-(((4-(8,8-dimethyl-3,4-dihydro-2H,8H-pyrano[2,3-f]chromen- 3-yl)-1,3-phenylene)bis(oxy))-di-(hexane-6,1-diyl))-di-(1-ethyl-4,5-di-(4-methoxyphenyl)-imidazol-2-ylidene)]-di-gold(I) (****6h****).* A yellow solid, yield 68.2 %. ^1^H NMR (500 MHz, DMSO-*d_6_*) *δ* 7.26 (dd, *J* = 15.7, 8.4 Hz, 6H, Ar**H**), 7.19 (d, *J* = 8.6 Hz, 2H, Ar**H**), 7.04 (d, *J* = 8.5 Hz, 1H, Ar**H**), 6.96-6.92 (m, 6H, Ar**H**), 6.88 (d, *J* = 8.7 Hz, 2H, Ar**H**), 6.83 (d, *J* = 8.4 Hz, 1H, C**H**=CH), 6.53-6.48 (m, 2H, Ar**H**), 6.44 (dd, *J* = 8.4, 2.4 Hz, 1H, Ar**H**), 6.27 (d, *J* = 8.2 Hz, 1H, Ar**H**), 5.62 (d, *J* = 9.9 Hz, 1H, CH=C**H**), 4.19 (d, *J* = 9.8 Hz, 1H), 4.05 (dq, *J* = 17.8, 10.6, 9.2 Hz, 8H), 3.96 (t, *J* = 10.1 Hz, 1H), 3.87 (dt, *J* = 17.0, 6.4 Hz, 4H), 3.74 (s, 6H, OC**H**_3_), 3.71 (s, 3H, OC**H**_3_), 3.67 (s, 3H, OC**H**_3_), 2.90 (dd, *J* = 15.7, 10.9 Hz, 1H), 2.75-2.69 (m, 1H), 1.58 (p, *J* = 7.0 Hz, 8H), 1.33 (s, 3H, C**H**_3_), 1.30 (s, 3H, C**H**_3_), 1.29-1.19 (m, 14H). ^13^C NMR (126 MHz, Chloroform-*d*) *δ* 172.48 (**C**-Au), 172.46 (**C**-Au), 160.14 (2C, Ar**C**OCH_3_), 160.11 (2C, Ar**C**OCH_3_), 159.10 (OAr**C**), 157.65 (OAr**C**), 151.81 (OAr**C**), 149.89 (OAr**C**), 131.84, 131.82, 130.89, 130.70, 130.65, 129.25, 128.83, 127.50, 121.61, 119.87, 119.83, 117.03, 114.66, 114.55, 114.31, 114.28, 109.82, 108.53, 105.03, 99.74, 75.55, 70.28 (O**C**H_2_), 67.81 (O**C**H_2_), 67.76 (O**C**H_2_), 55.28 (2C, O**C**H_3_), 55.25 (2C, O**C**H_3_), 48.87, 48.76, 44.15, 31.59, 31.09, 30.92, 29.71, 29.01, 28.80, 27.79, 27.65, 26.12, 25.93, 25.42, 25.34, 16.95. ESI-MS (+) [m/z]: 748.47 [M-2Br]^2+^. Purity > 95 % by HPLC.

3.9.*[Dibromo(R)-3,3'-(((4-(8,8-dimethyl-3,4-dihydro-2H,8H-pyrano[2,3-f]chromen- 3-yl)-1,3-phenylene)bis(oxy))-di-(hexane-6,1-diyl))-di-(1-ethyl-4,5-di-(4-fluorophenyl)-imidazol-2-ylidene)]-di-gold(I) (****6i****).* A yellowish solid, yield 45.7 %. ^1^H NMR (500 MHz, DMSO-*d_6_*) *δ* 7.45-7.39 (m, 6H, Ar**H**), 7.36 (dd, *J* = 8.5, 5.4 Hz, 2H, Ar**H**), 7.28-7.22 (m, 6H, Ar**H**), 7.19 (t, *J* = 8.7 Hz, 2H, Ar**H**), 7.04 (d, *J* = 8.4 Hz, 1H, Ar**H**), 6.83 (d, *J* = 8.3 Hz, 1H, C**H**=CH), 6.53- 6.48 (m, 2H, Ar**H**), 6.44 (dd, *J* = 8.5, 2.3 Hz, 1H, Ar**H**), 6.27 (d, *J* = 8.2 Hz, 1H, Ar**H**), 5.63 (d, *J* = 9.9 Hz, 1H, CH=C**H**), 4.19 (d, *J* = 11.1 Hz, 1H), 4.07 (dp, *J* = 22.6, 7.6 Hz, 8H), 3.97 (t, *J* = 10.1 Hz, 1H), 3.90 (t, *J* = 6.3 Hz, 2H), 3.86 (t, *J* = 6.5 Hz, 2H), 3.31 (s, 1H), 2.90 (dd, *J* = 15.7, 11.0 Hz, 1H), 2.75-2.68 (m, 1H), 1.59 (dq, *J* = 14.6, 7.2 Hz, 8H), 1.33 (s, 3H, C**H**_3_), 1.30 (s, 3H, C**H**_3_), 1.30-1.21 (m, 14H). ^13^C NMR (126 MHz, DMSO-*d_6_*) *δ* 171.57 (**C**-Au), 171.50 (**C**-Au), 163.50 (2C, Ar**C**-F), 161.53 (2C, Ar**C**-F), 158.65 (OAr**C**), 157.12 (OAr**C**), 151.24 (OAr**C**), 149.21 (OAr**C**), 133.16, 133.13, 133.09, 130.23, 130.15, 130.06, 129.99, 129.34, 129.22, 127.57, 123.66, 123.63, 120.90, 116.34, 116.00, 115.91, 115.83, 115.74, 114.50, 109.03, 108.19, 105.35, 99.55, 75.24, 69.60 (O**C**H_2_), 67.38 (O**C**H_2_), 67.20 (O**C**H_2_), 48.43, 48.27, 43.91, 31.15, 30.04, 30.00, 29.82, 28.31, 28.07, 27.37, 27.23, 25.25, 25.09, 24.69, 24.59, 16.41, 16.40. ESI-MS (+) [m/z]: 1531.93 [M-Br] ^+^. Purity > 95 % by HPLC.

3.10. *[Bromo(R)-(1-(5-(4-(8,8-dimethyl-3,4-dihydro-2H,8H-pyrano[2,3-f]chromen- 3-yl)-3-hydroxyphenoxy)pentyl)-3-ethyl-1,3-dihydro-2H-imidazol-2-ylidene)]-gold(I) (****6j****).* A purple solid, yield 51.0%. ^1^H NMR (500 MHz, Chloroform-*d*) *δ* 6.97 (d, *J* = 8.5 Hz, 1H, Ar**H**), 6.95-6.92 (m, 2H, NC**H**=C**H**N), 6.82 (d, *J* = 8.2 Hz, 1H, CH=C**H**), 6.64 (d, *J* = 9.9 Hz, 1H, Ar**H**), 6.55 (d, *J* = 2.4 Hz, 1H, Ar**H**), 6.42 (dd, *J* = 8.5, 2.5 Hz, 1H, Ar**H**), 6.36 (d, *J* = 8.2 Hz, 1H, Ar**H**), 5.63 (s, 1H), 5.55 (d, *J* = 9.8 Hz, 1H, CH=C**H**), 4.38 (ddd, *J* = 10.4, 3.5, 2.0 Hz, 1H), 4.21 (q, *J* = 7.3 Hz, 2H), 4.17 (t, *J* = 7.3 Hz, 2H), 4.03 (t, *J* = 10.2 Hz, 1H), 3.95 (t, *J* = 6.1 Hz, 2H), 3.50 (td, *J* = 10.0, 9.4, 4.5 Hz, 1H), 2.98 (dd, *J* = 15.7, 10.8 Hz, 1H), 2.85 (ddd, *J* = 15.8, 5.4, 2.0 Hz, 1H), 1.94 (p, *J* = 7.4 Hz, 2H), 1.82 (q, *J* = 6.8 Hz, 2H), 1.52 (t, *J* = 7.3 Hz, 2H), 1.45 (t, *J* = 7.3 Hz, 3H), 1.42 (s, 3H), 1.41 (s, 3H). ^13^C NMR (126 MHz, Chloroform-*d*) *δ* 169.82 (**C**-Au), 157.47 (OAr**C**), 155.86 (OAr**C**), 151.90 (OAr**C**), 149.83 (OAr**C**), 129.25, 129.04, 127.78, 121.32, 120.54, 119.84, 116.89, 109.84, 108.64, 107.28, 100.34, 75.61, 70.35, 67.41, 51.09, 46.53, 31.56, 30.78, 30.43, 28.29, 27.75, 27.70, 22.71, 16.43. ESI-MS (+) [m/z]: 685.42 [M-Br]^+^. Purity > 95% by HPLC.

3.11. *[Bromo(R)-(1-(5-(2-(8,8-dimethyl-3,4-dihydro-2H,8H-pyrano[2,3-f]chromen- 3-yl)-5-hydroxyphenoxy)pentyl)-3-ethyl-1,3-dihydro-2H-imidazol-2-ylidene)]-gold(I) (****6k****).* A white solid, yield 34.5%. ^1^H NMR (500 MHz, Chloroform-*d*) *δ* 6.94 (d, *J* = 8.3 Hz, 1H, NC**H**=C**H**N), 6.86 (d, *J* = 2.1 Hz, 1H, NC**H**=C**H**N), 6.83 (d, *J* = 8.3 Hz, 1H, CH=C**H**), 6.78 (s, 1H, Ar**H**), 6.64 (d, *J* = 10.0 Hz, 1H, Ar**H**), 6.53 (d, *J* = 2.4 Hz, 1H, Ar**H**), 6.43-6.39 (m, 1H, Ar**H**), 6.37 (d, *J* = 8.2 Hz, 1H, Ar**H**), 5.57 (d, *J* = 9.9 Hz, 1H, CH=C**H**), 4.30 (d, *J* = 10.4 Hz, 1H), 4.18-4.11 (m, 4H), 4.02-3.92 (m, 3H), 3.47 (d, *J* = 12.5 Hz, 1H), 2.93 (dd, *J* = 15.6, 11.3 Hz, 1H), 2.79 (dd, *J* = 16.2, 5.2 Hz, 1H), 1.94 (p, *J* = 7.5 Hz, 2H), 1.85 (q, *J* = 6.9 Hz, 2H), 1.51 (t, *J* = 7.6 Hz, 2H), 1.42 (d, *J* = 5.8 Hz, 9H). ^13^C NMR (126 MHz, Chloroform-*d*) *δ* 173.62 (**C**-Au), 157.65 (OAr**C**), 155.89 (OAr**C**), 152.04 (OAr**C**), 149.96 (OAr**C**), 129.39, 129.19, 127.91, 121.50, 120.60, 119.87, 117.03, 114.80, 109.98, 108.78, 107.32, 100.34, 75.75, 70.47, 67.58, 51.13, 46.54, 30.90, 30.61, 30.34, 29.85, 28.47, 27.88, 27.84, 22.95, 16.56. ESI-MS (+) [m/z]: 685.42 [M-Br]^+^. Purity > 95% by HPLC.

**Supporting Tables and Figures**

1. **Scheme S1.** Synthesis of Gold(I)−NHC Complexes **6a-6i**.

Reagent and conditions: (I) K_2_CO_3_, DMF, RT, 2 h; (II) CH_3_CN, 80 °C, 2-5 days; (III) a. Ag_2_O, DCM, N_2_, darkness, RT, overnight; b. (CH_3_)_2_SAuCl, DCM, N_2_, darkness, RT, 12 h; c. NaBr, DCM, N_2_, darkness, RT, 12 h.

1. **Scheme S2.** Synthesis of Gold(I)−NHC Complexes **6j** and **6k**.

Reagent and conditions: (I) K_2_CO_3_, CH_3_CN, 55 °C, 6 h; (II) CH_3_CN, 80 °C, 2-5 days; (III) a. Ag_2_O, DCM, N_2_, darkness, RT, overnight; b. (CH_3_)_2_SAuCl, DCM, N_2_, darkness, RT, 12 h; c. NaBr, DCM, N_2_, darkness, RT, 12 h.

1. **NMR Spectra for Synthesized Complexes**

**Figure S1.** ^1^H NMR spectrum of **6a** (500 MHz, CDCl_3_)

**Figure S2.** ^13^C NMR spectrum of **6a** (126 MHz, DMSO-*d_6_*)

**Figure S3.** ^1^H NMR spectrum of **6b** (500 MHz, CDCl_3_)

**Figure S4.** ^13^C NMR spectrum of **6b** (126 MHz, CDCl_3_)

**Figure S5.** ^1^H NMR spectrum of **6c** (500 MHz, DMSO-*d*_6_)

**Figure S6.** ^13^C NMR spectrum of **6c** (126 MHz, DMSO-*d_6_*)

**Figure S7.** ^1^H NMR spectrum of **6d** (500 MHz, CDCl_3_)

**Figure S8.** ^13^C NMR spectrum of **6d** (126 MHz, CDCl_3_)

**Figure S9.** ^1^H NMR spectrum of **6e** (500 MHz, DMSO-*d*_6_)

**Figure S10.** ^13^C NMR spectrum of **6e** (126 MHz, DMSO-*d_6_*)

**Figure S11.** ^1^H NMR spectrum of **6f** (500 MHz, DMSO-*d*_6_)

**Figure S12.** ^13^C NMR spectrum of **6f** (126 MHz, DMSO-*d_6_*)

**Figure S13.** ^1^H NMR spectrum of **6g** (500 MHz, DMSO-*d*_6_)

**Figure S14.** ^13^C NMR spectrum of **6g** (126 MHz, CDCl_3_)

**Figure S15.** ^1^H NMR spectrum of **6h** (500 MHz, DMSO-*d*_6_)

**Figure S16.** ^13^C NMR spectrum of **6h** (126 MHz, CDCl_3_)

**Figure S17.** ^1^H NMR spectrum of **6i** (500 MHz, DMSO-*d*_6_)

**Figure S18.** ^13^C NMR spectrum of **6i** (126 MHz, DMSO-*d_6_*)

**Figure S19.** ^1^H NMR spectrum of **6j** (500 MHz, CDCl_3_)

**Figure S20.** ^13^C NMR spectrum of **6j** (126 MHz, CDCl_3_)

**Figure S21.** ^1^H NMR spectrum of **6k** (500 MHz, CDCl_3_)

**Figure S22.** ^13^C NMR spectrum of **6k** (126 MHz, CDCl_3_)

1. **HPLC Chromatogram for purity assessment**

HPLC analysis was conducted utilizing Waters ACQUITY HPLC system (MA, USA), which was fitted with a C18 column. Acetonitrile/water was used as the mobile phase, and the flow rate was 0.7 mL/min. The detection was at 254 nm wavelength at 25 °C. The ratio of the mobile phases in the gradient elution changes with time as shown in the table below:

A.

| Time (min) | Rate (mL/min) | Water (%) | Acetonitrile |
| --- | --- | --- | --- |
| 0 | 0.8 | 95 | 5 |
| 5 | 0.8 | 0 | 100 |
| 9 | 0.8 | 0 | 100 |
| 13 | 0.8 | 95 | 5 |
| 15 | 0.8 | 95 | 5 |

B.

| Time (min) | Rate (mL/min) | Water (%) | Acetonitrile |
| --- | --- | --- | --- |
| 0 | 0.7 | 90 | 10 |
| 3 | 0.7 | 0 | 100 |
| 6 | 0.7 | 0 | 100 |
| 9 | 0.7 | 90 | 10 |
| 11 | 0.7 | 90 | 10 |

C.

| Time (min) | Rate (mL/min) | Water (%) | Acetonitrile |
| --- | --- | --- | --- |
| 0 | 0.6 | 90 | 10 |
| 6 | 0.6 | 0 | 100 |
| 9 | 0.6 | 0 | 100 |
| 15 | 0.6 | 90 | 10 |
| 20 | 0.6 | 90 | 10 |

D.

| Time (min) | Rate (mL/min) | Water (%) | Acetonitrile |
| --- | --- | --- | --- |
| 0 | 0.5 | 90 | 10 |
| 3 | 0.5 | 0 | 100 |
| 6 | 0.5 | 0 | 100 |
| 9 | 0.5 | 90 | 10 |
| 10 | 0.5 | 90 | 10 |

**Figure S23.** HPLC spectrum of **6a** (method A)

**Figure S24.** HPLC spectrum of **6b** (method A)

**Figure S25.** HPLC spectrum of **6c** (method A)

**Figure S26.** HPLC spectrum of **6d** (method B)

**Figure S27.** HPLC spectrum of **6e** (method C)

**Figure S28.** HPLC spectrum of **6f** (method C)

**Figure S29.** HPLC spectrum of **6g** (method C)

**Figure S30.** HPLC spectrum of **6h** (method C)

**Figure S31.** HPLC spectrum of **6i** (method C)

**Figure S32.** HPLC spectrum of **6j** (method D)

**Figure S33.** HPLC spectrum of **6k** (method D)

1. **Stability Test of 6d and AF**

**Figure S34.** ^1^H NMR spectra of **6d** in DMSO-*d_6_* measured over 72 h.

**Figure S35.** ^1^H NMR spectra of **AF** in DMSO-*d_6_* measured over 72 h.

**Figure S36.** ^1^H NMR spectra of **6d** in DMSO-*d_6_*/D_2_O (v_1_/v_2_ = 9:1) measured over 72 h.

**Figure S37.** ^1^H NMR spectra of **AF** in DMSO-*d_6_*/D_2_O (v_1_/v_2_ = 9:1) measured over 72 h.

**Figure S38.** ^1^H NMR spectra of **6d** in DMSO-*d_6_*/D_2_O (v_1_/v_2_ = 9:1) containing GSH measured over 72 h.

**Figure S39.** ^1^H NMR spectra of **AF** in DMSO-*d_6_*/D_2_O (v_1_/v_2_ = 9:1) containing GSH measured over 72 h.

| **Compound ID** | **Species** | **Percent Remaining (%)(Mean, n=2)** | | | | | | **-k** | **T_1/2_ (minute)** |
| --- | --- | --- | --- | --- | --- | --- | --- | --- | --- |
|  |  | **0 min** | **5 min** | **15 min** | **30 min** | **60 min** | **120 min** |  |  |
| 6d | Mouse | 100 | 98.8 | 107 | 103 | 103 | 108 | -0.000500 | ∞ |
| Propantheline bromide |  | 100 | 88.5 | 66.0 | 47.5 | 21.8 | 7.63 | 0.0216 | 32.1 |

**Table S1.** Remaining percentage and metabolic stability of test complex **6d** and control complex in whole blood.

1. **Solubility of 6d**


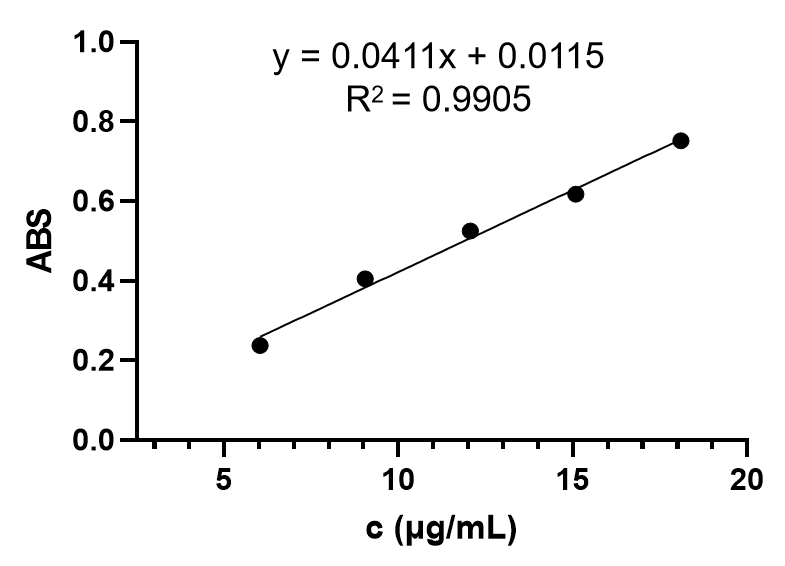


**Figure S40.** The standard curve of complex **6d** in PBS: DMF = 200 : 1 at 298K.

1. ***In vitro* researches of mechanism**


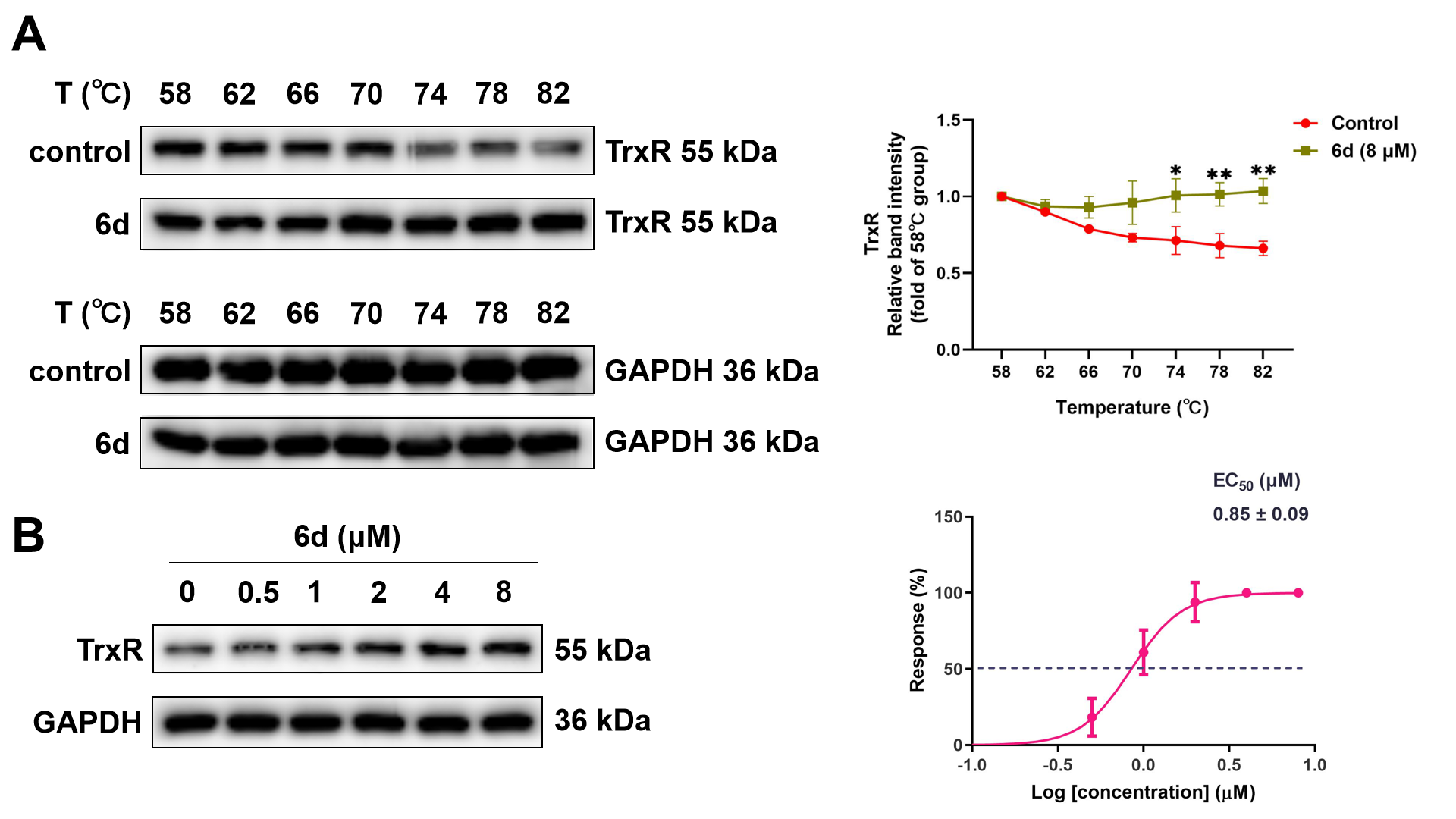


**Figure S41.** Assessment of the binding efficiency between the **6d** and TrxR target. (A) CETSA with TrxR and GAPDH antibody (8 μM of **6d**). (B) ITDR assay with TrxR and GAPDH antibody (78 °C). Data are expressed as the mean ± SD (n = 3); Student’s *t*-test, compared with Control group; **p* < 0.05 and ***p*< 0.01.


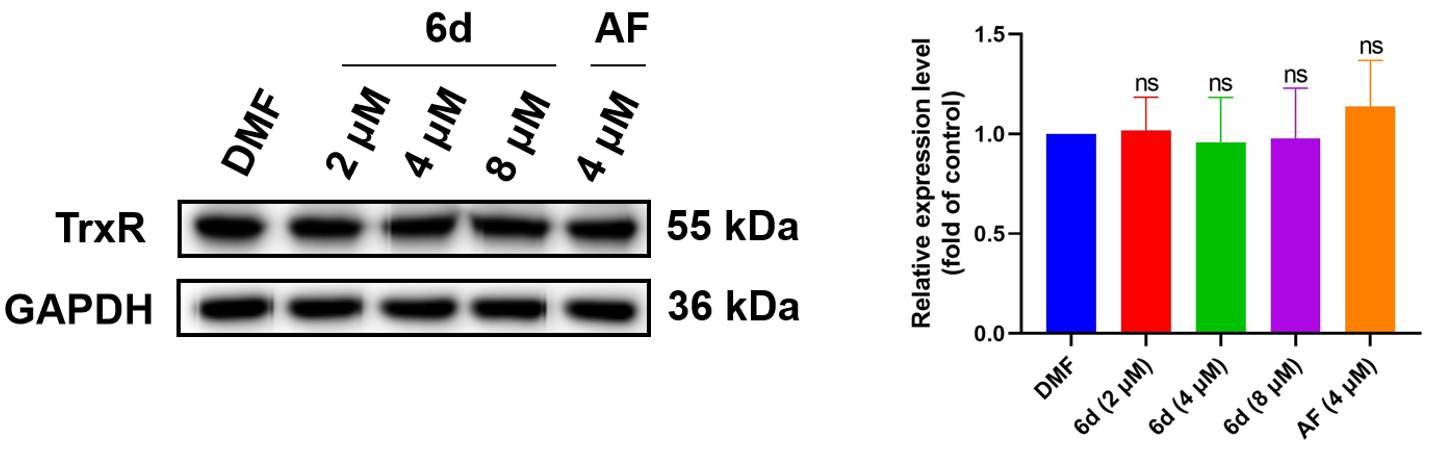


**Figure S42.** Western blot analysis for expression level of TrxR in HepG2 cells by 24 h complex **6d** treatment. Data are expressed as the mean ± SD (n = 3); Student’s *t*-test, compared with DMF group; ns > 0.05.


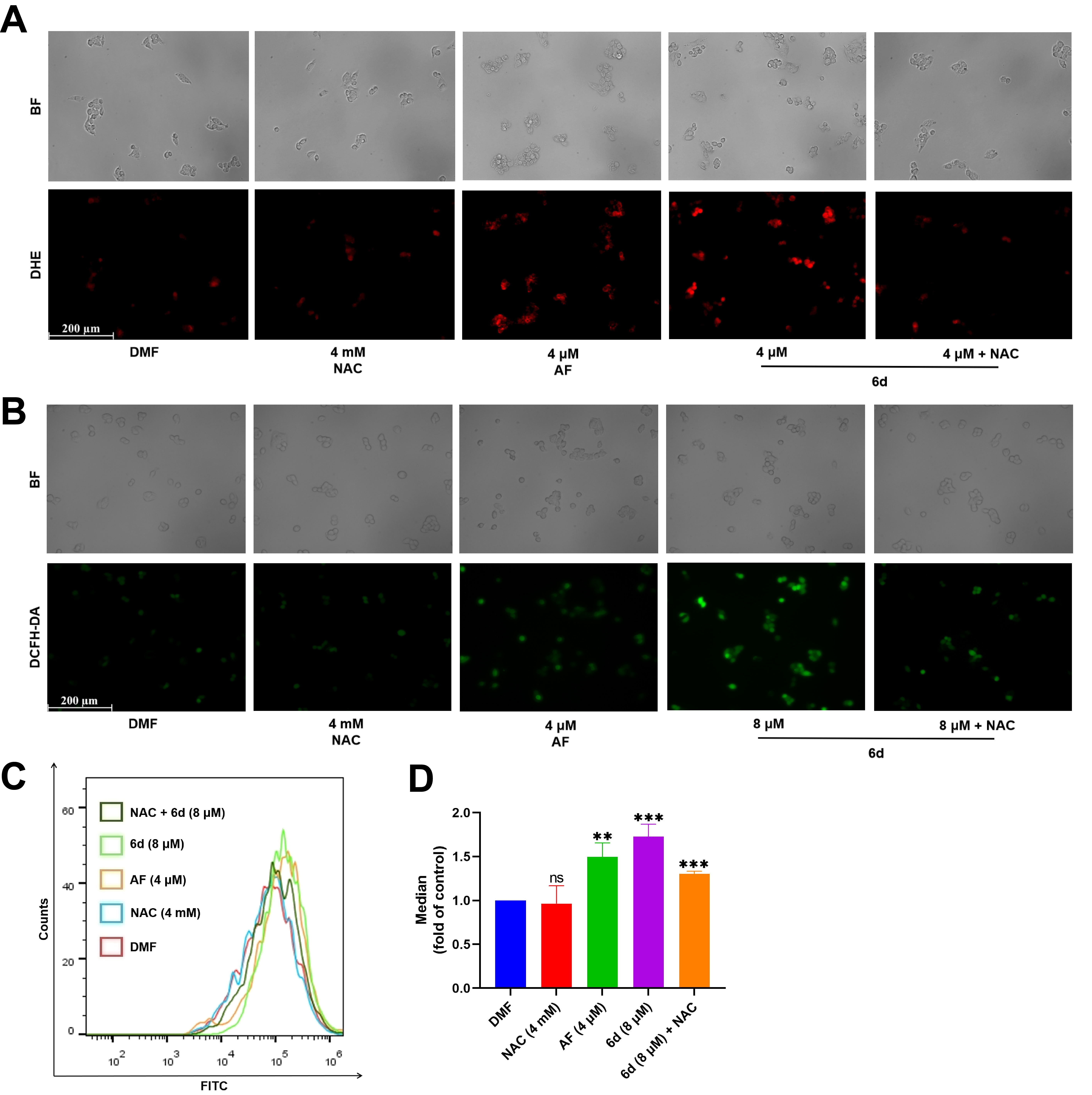


**Figure S43.** Effects of complex **6d** on intracellular ROS. (A) **6d**-induced ROS production. ROS positive cells were identified in images merging of bright-field and fluorescence images regions after **6d** treatment by DHE assays (scale bar = 200 μm). (B) ROS positive cells were identified in images merging of bright-field and fluorescence images regions after **6d** treatment by DCFH-DA assays (scale bar = 200 μm). (C, D) Quantification of intracellular ROS level in HepG2 cells by flow cytometry (using DCFH-DA probe). Data are expressed as the mean ± SD (n = 3); Student’s *t*-test, compared with DMF group; ns > 0.05, ***p* < 0.01 and ****p*< 0.001.


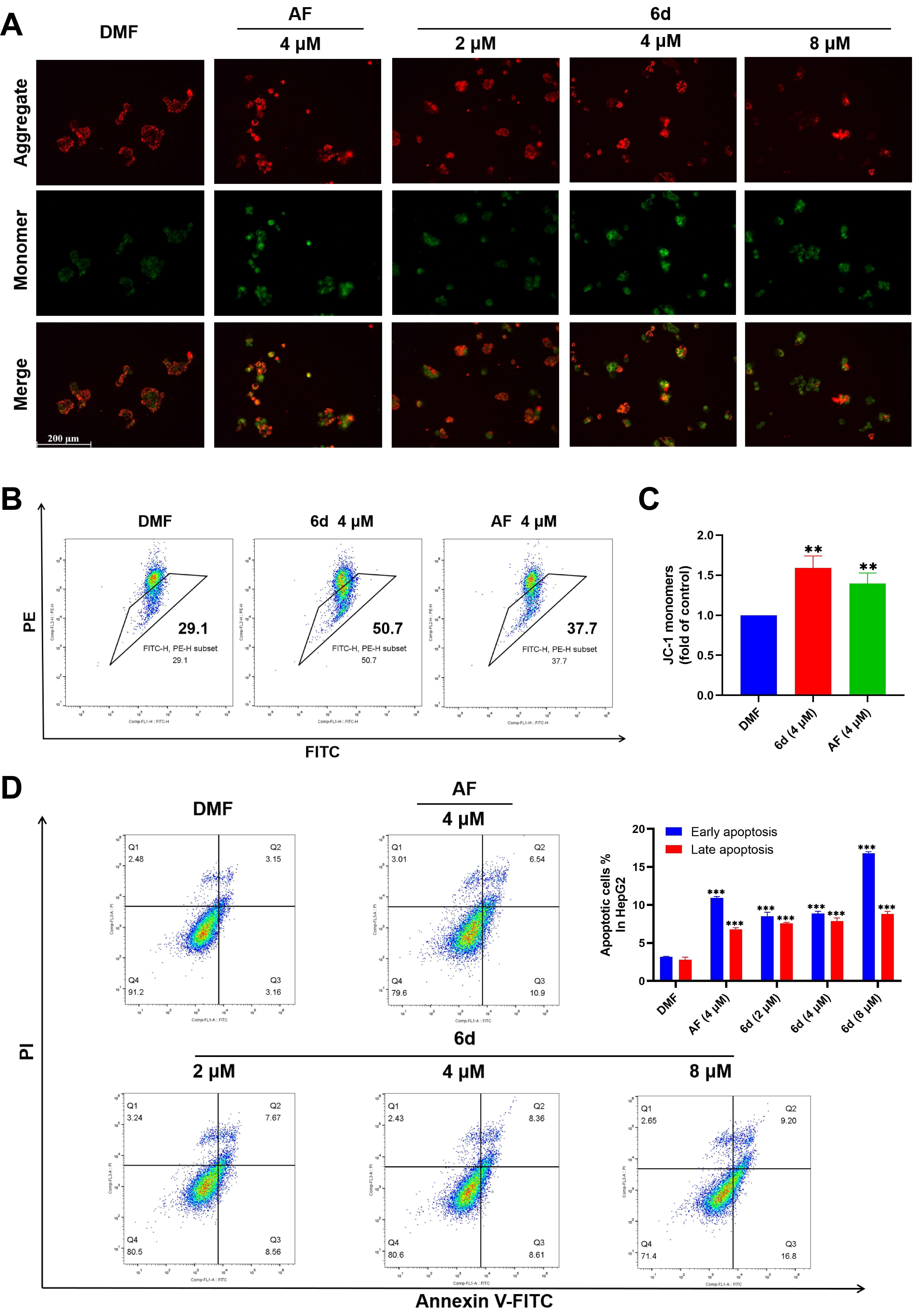


**Figure S44.** **6d** induced mitochondrial damage and apoptosis in HepG2 cells. (A) Mitochondrial membrane damage showed by JC-1 staining (scale bar = 200 μm). (B, C) Quantification of mitochondrial membrane potential (MMP) by flow cytometry. (D) Detection of apoptotic cells after treatment with **6d** for 24 h by flow cytometry. Data are expressed as the mean ± SD (n = 3); Student’s *t*-test, compared with DMF group; ***p* < 0.01 and ****p* < 0.001.


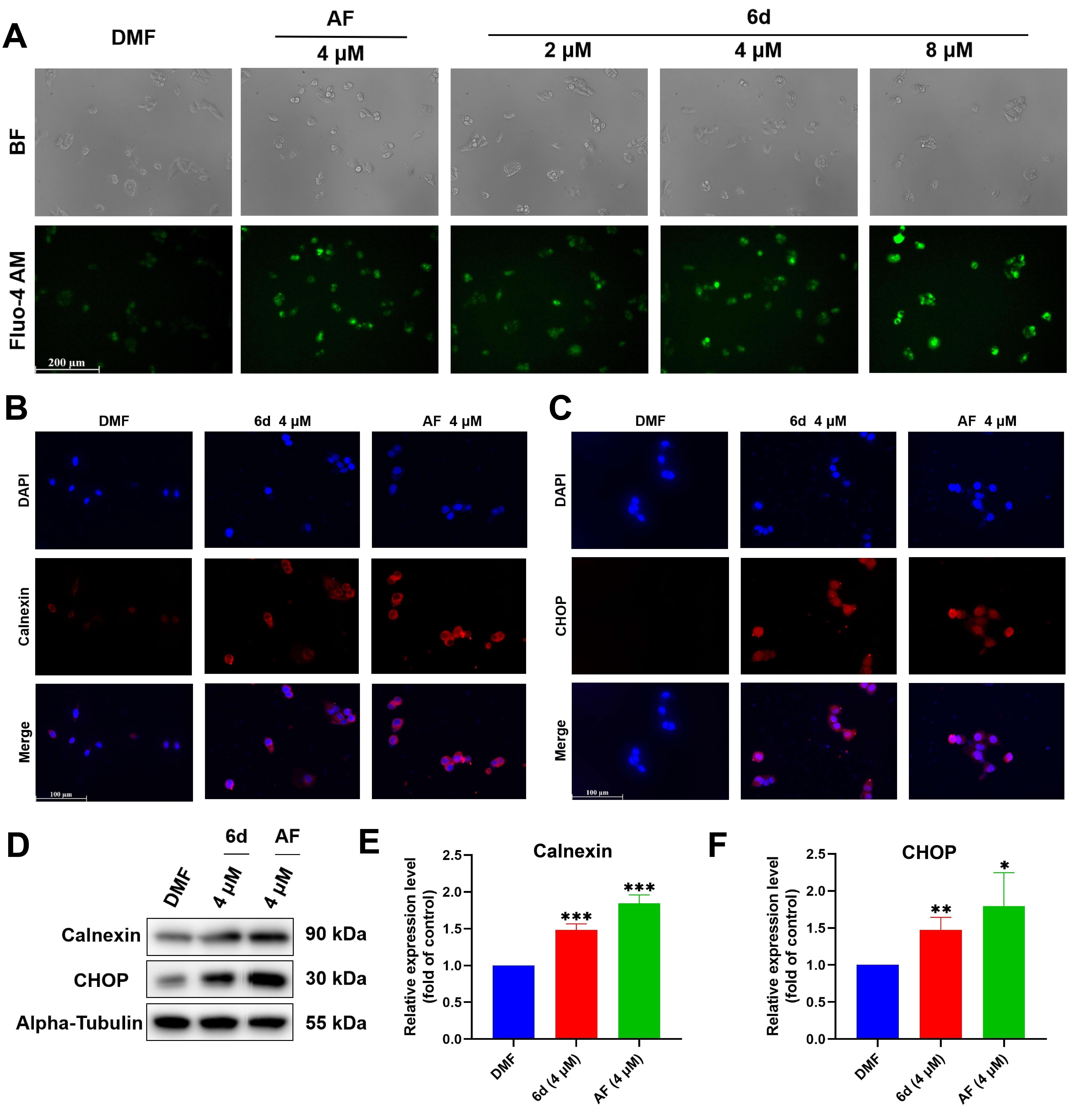


**Figure S45.** **6d** induced ERS. (A) Fluorescent microscopy images of a Fluo-4 AM assay (scale bar = 200 μm). (B, C) Detection of ERS associated protein calnexin and CHOP by immunofluorescent microscopy images (scale bar = 100 μm). (D, E, F) Western blot analysis of calnexin and CHOP. Data are expressed as the mean ± SD (n = 3); Student’s *t*-test, compared with DMF group; **p* < 0.05, ***p* < 0.01 and ****p* < 0.001.


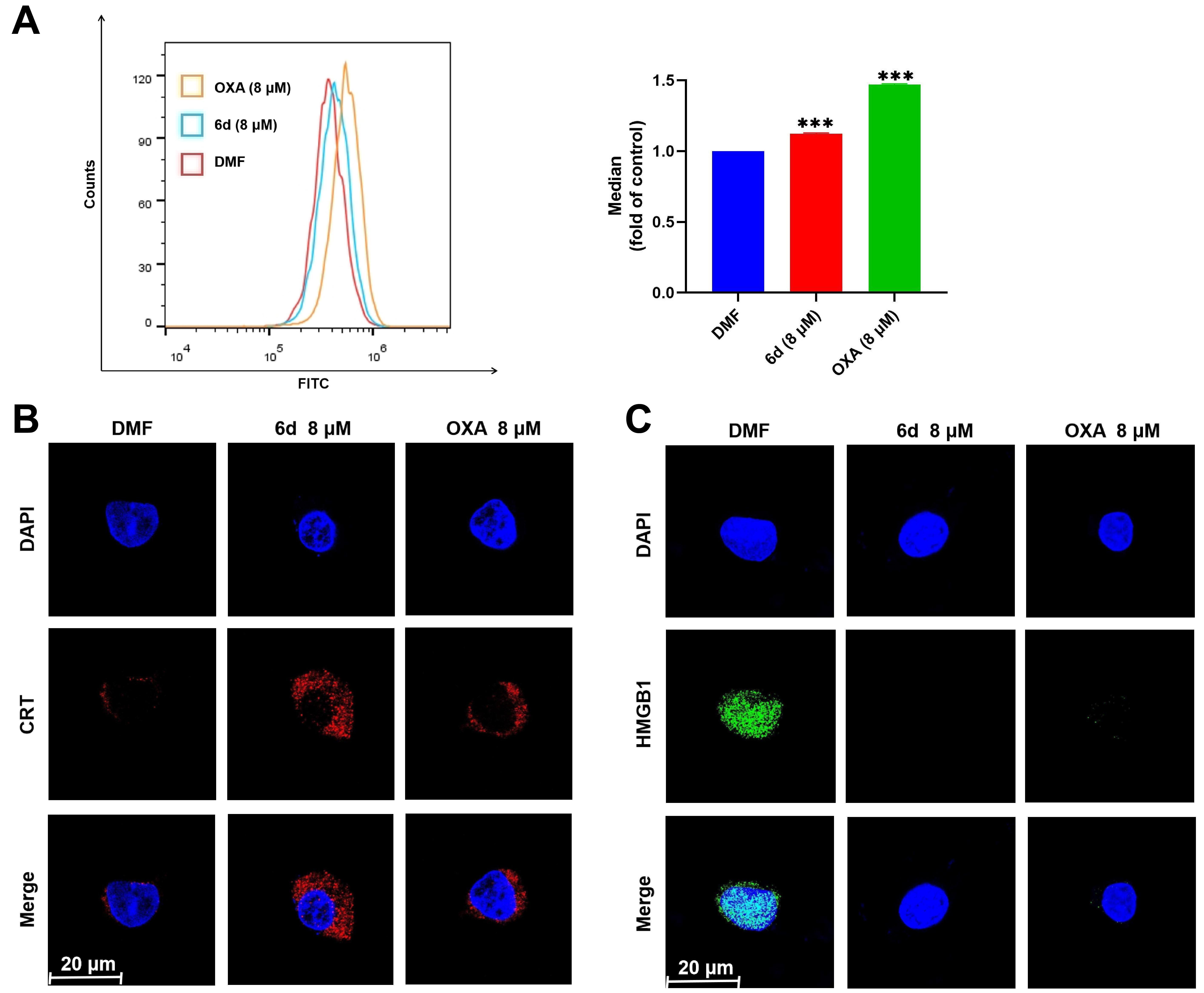


**Figure S46.** **6d** induced ICD. (A) Flow cytometry analysis of CRT exposure on HepG2 cell surface by **6d**. Data are expressed as the mean ± SD (n = 3); Student’s *t*-test, compared with DMF group; ****p* < 0.001. (B) Immunofluorescence analysis of CRT exposure on the surface of HepG2 cells after 24 h treatment (scale bar = 20 μm). (C) Immunofluorescence analysis of HMGB1 excretion from HepG2 cells after 24 h incubation (scale bar = 20 μm).


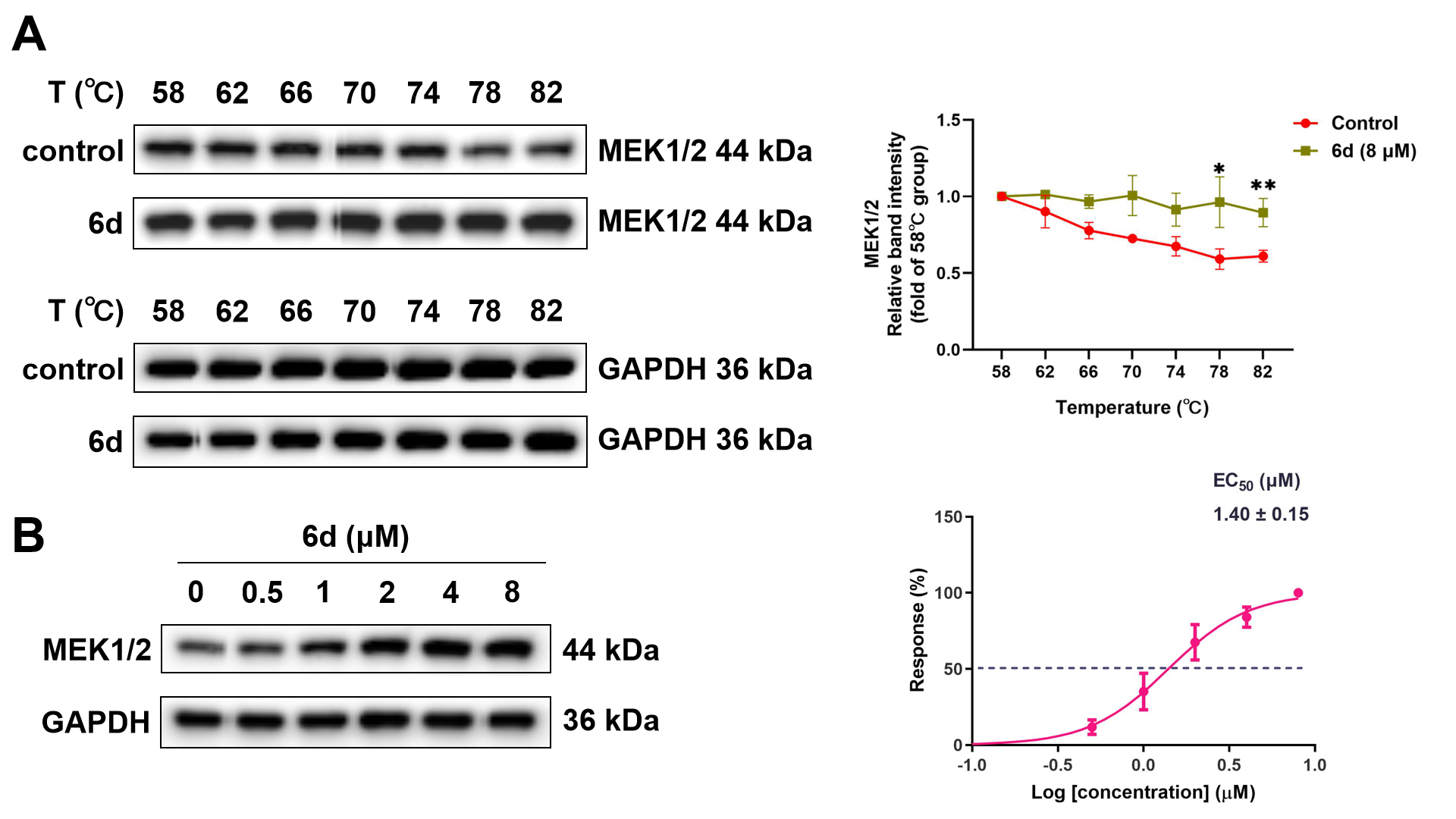


**Figure S47.** Assessment of the binding efficiency between the **6d** and MEK1/2 target. (A) CETSA with MEK1/2 and GAPDH antibody (8 μM of **6d**). (B) ITDR assay with MEK1/2 and GAPDH antibody (78 °C). Data are expressed as the mean ± SD (n = 3); Student’s *t*-test, compared with Control group; **p* < 0.05 and ***p*< 0.01.


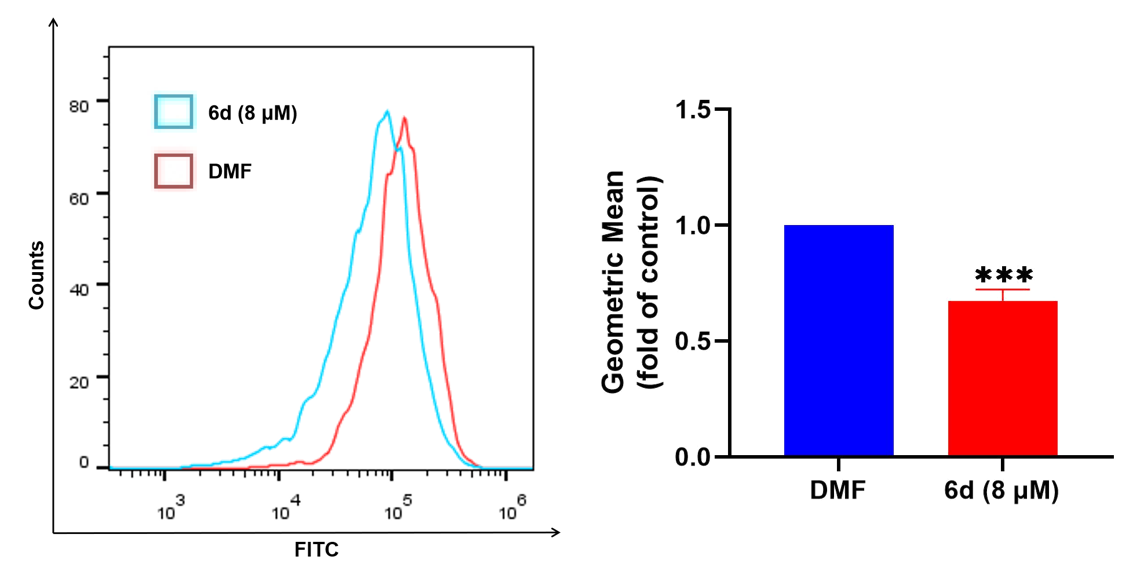


**Figure S48.** Flow cytometry analysis of PD-L1 expression suppression on HepG2 cell surface by **6d**. Data are expressed as the mean ± SD (n = 3); Student’s *t*-test, compared with DMF group; ****p* < 0.001.


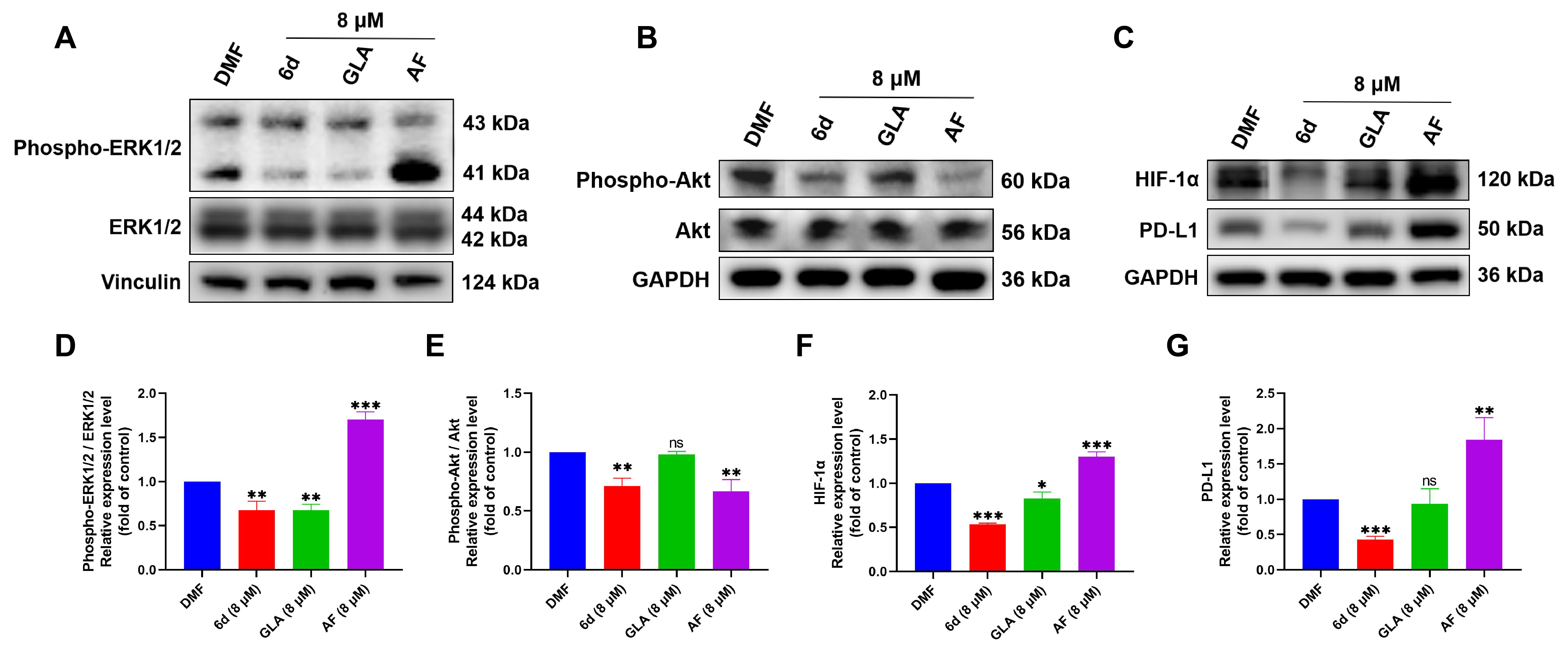


**Figure S49.** The synergistic effects of gold and GLA units in **6d**. (A) Western blot analysis of phosphorylated ERK1/2 levels after 24 h of **6d** treatment. (B) Western blot analysis of phosphorylated Akt levels after 24 h of **6d** treatment. (C) Western blot analysis of HIF-1α and PD-L1 levels after 24 h of **6d** treatment. (D) The relative expression quantifications of phosphorylated ERK1/2 in A. (E) The relative expression quantifications of phosphorylated Akt in B. (F) The relative expression quantifications of HIF-1α in C. (G) The relative expression quantifications of PD-L1 in C. Data are expressed as the mean ± SD (n = 3); Student’s *t*-test, compared with DMF group; ns > 0.05, **p* < 0.05, ***p*< 0.01 and ****p* < 0.001.

1. ***In vivo* researches of mechanism**


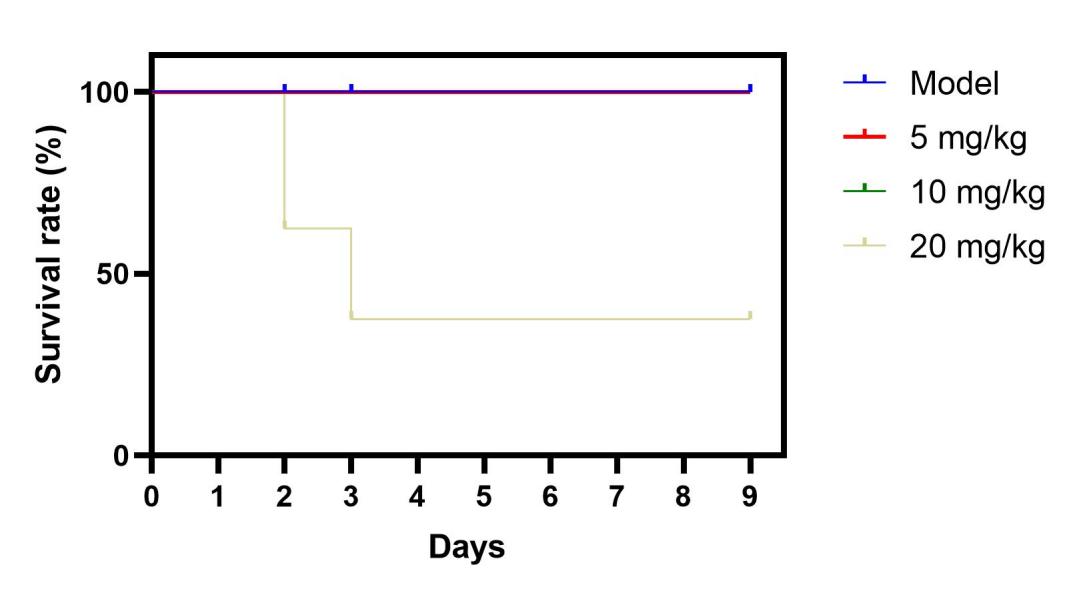


**Figure S50.** Kaplan-Meier curves of acute toxic effects of **6d** (n=8 per group). Under the present experimental conditions, the lethal median dose was between 10 and 20 mg/kg.

**
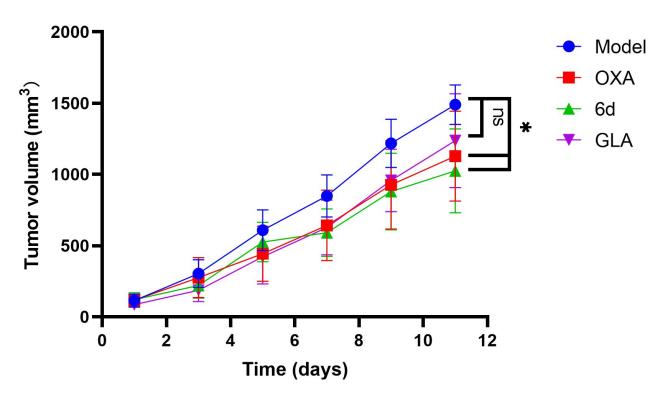
**

**Figure S51.** The recorded *in vivo* tumor volume every two days after treatment. Data are expressed as the mean ± SD (n = 5); Student’s *t*-test, compared with Model group; ns > 0.05 and **p* < 0.05.


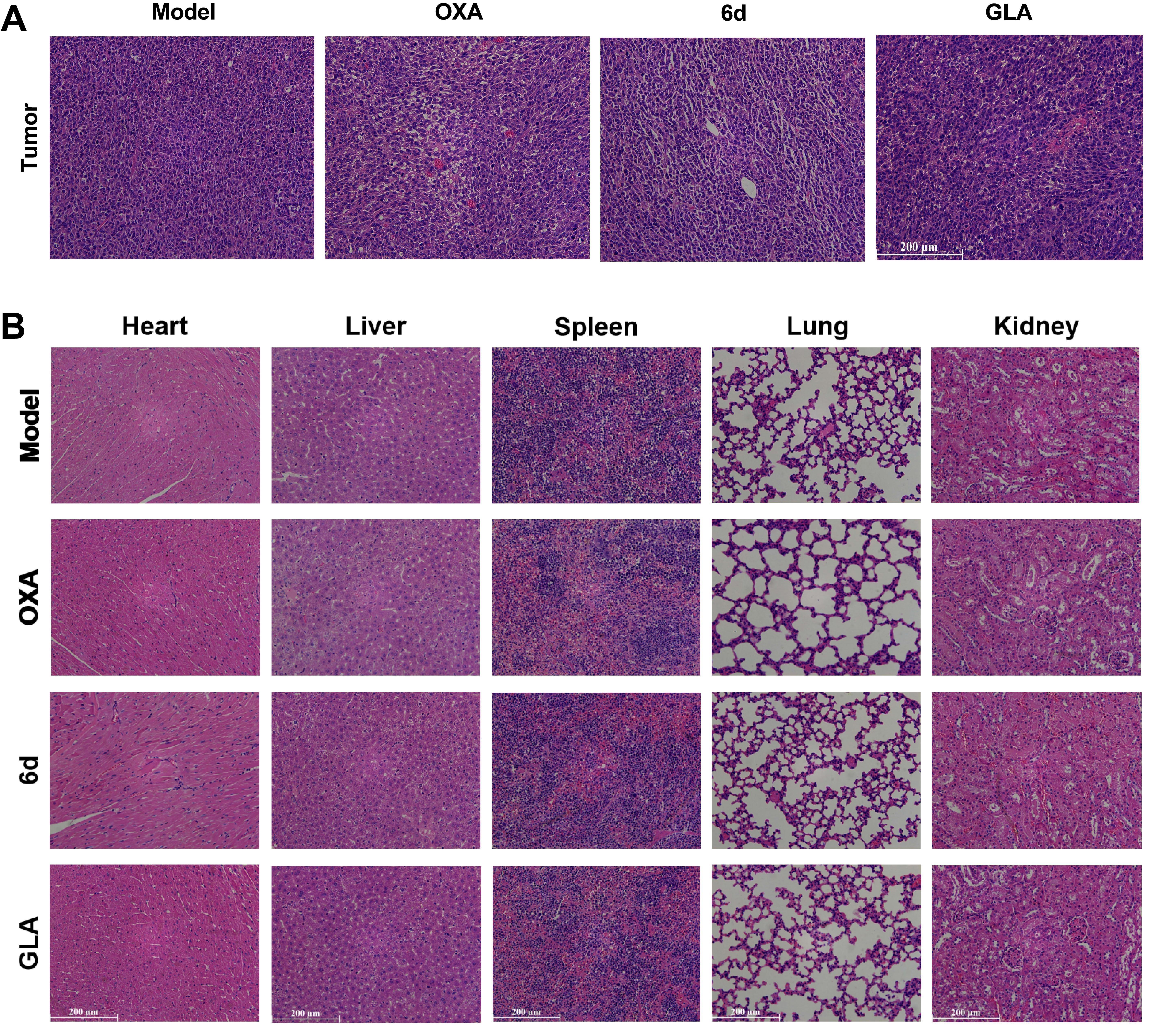


**Figure S52.** H&E staining images of mice tissue. (A) Tumor tissues. (B) Heart, liver, spleen, lung and kidney tissues. (scale bar = 200 μm)


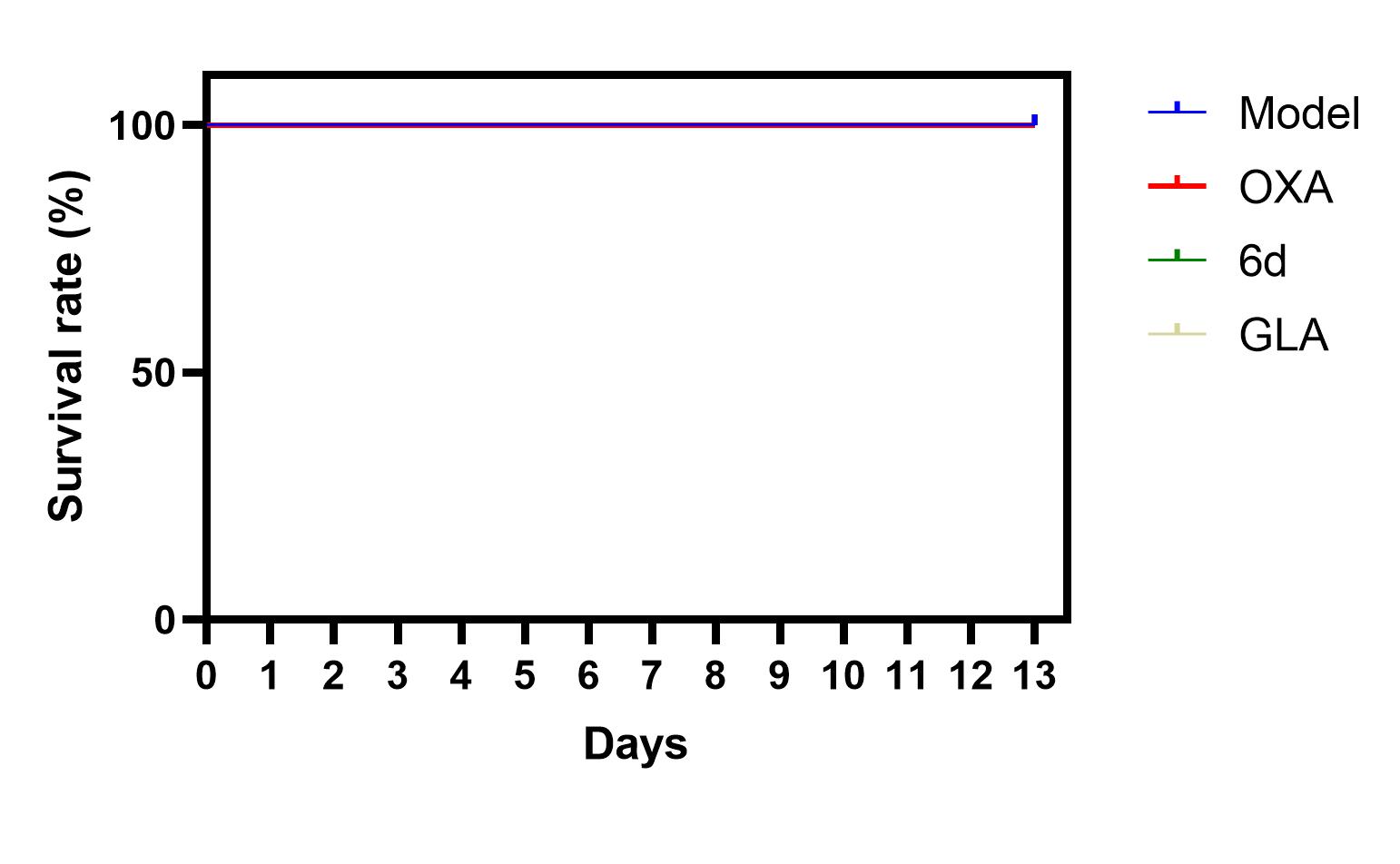


**Figure S53.** Kaplan-Meier curves of all groups (n=5 per group). The vertical tick at day 13 indicates scheduled sacrifice of all animals, and no deaths occurred during the study.


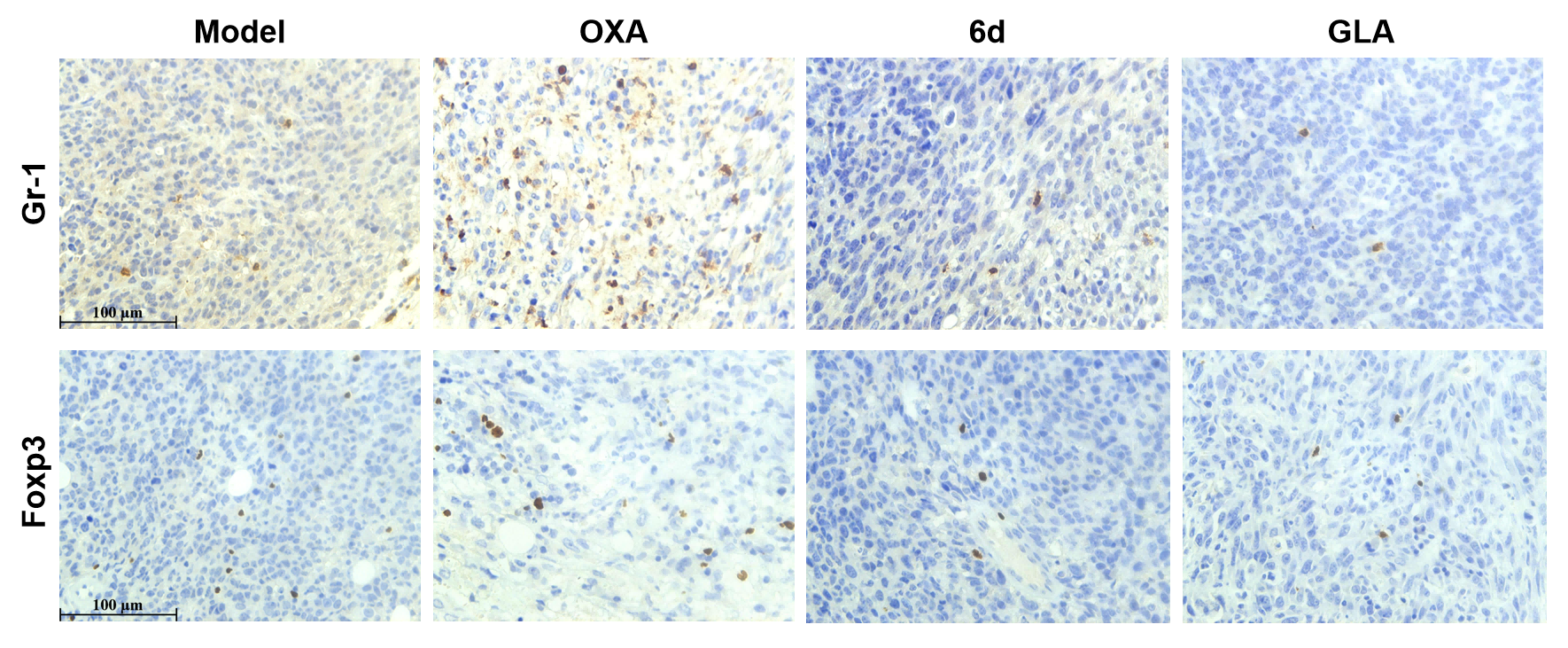


**Figure S54.** Immunohistochemistry analysis of MDSCs (Gr-1) and Tregs (Foxp3) infiltration in tumor tissue. (scale bar = 100 μm)


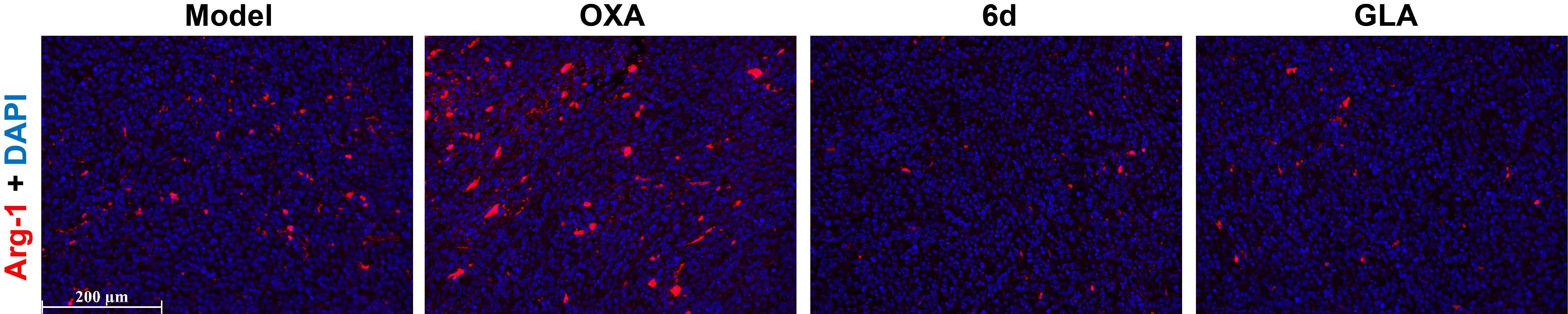


**Figure S55.** Immunofluorescence analysis of Arg-1 expression in tumor tissue (scale bar = 200 μm)


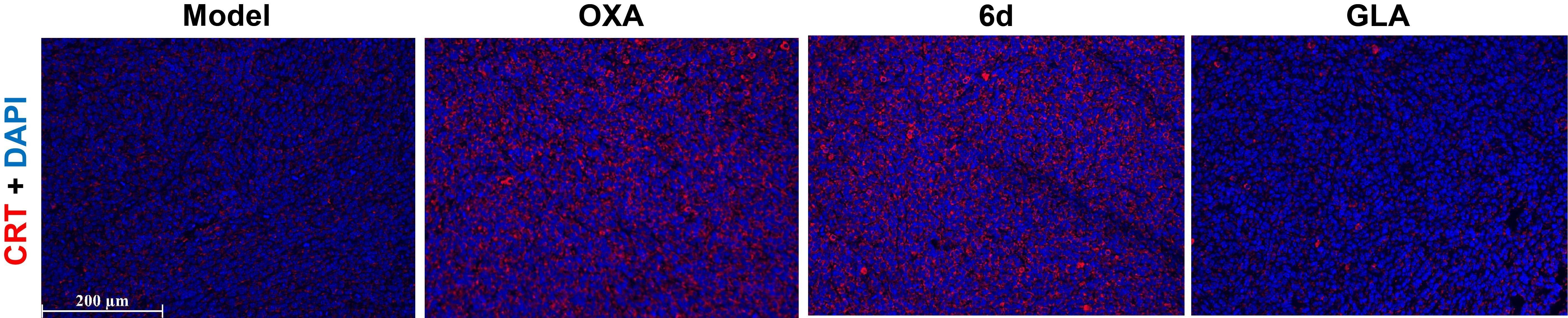


**Figure S56.** Immunofluorescence analysis of CRT exposure in tumor tissue (scale bar = 200 μm).


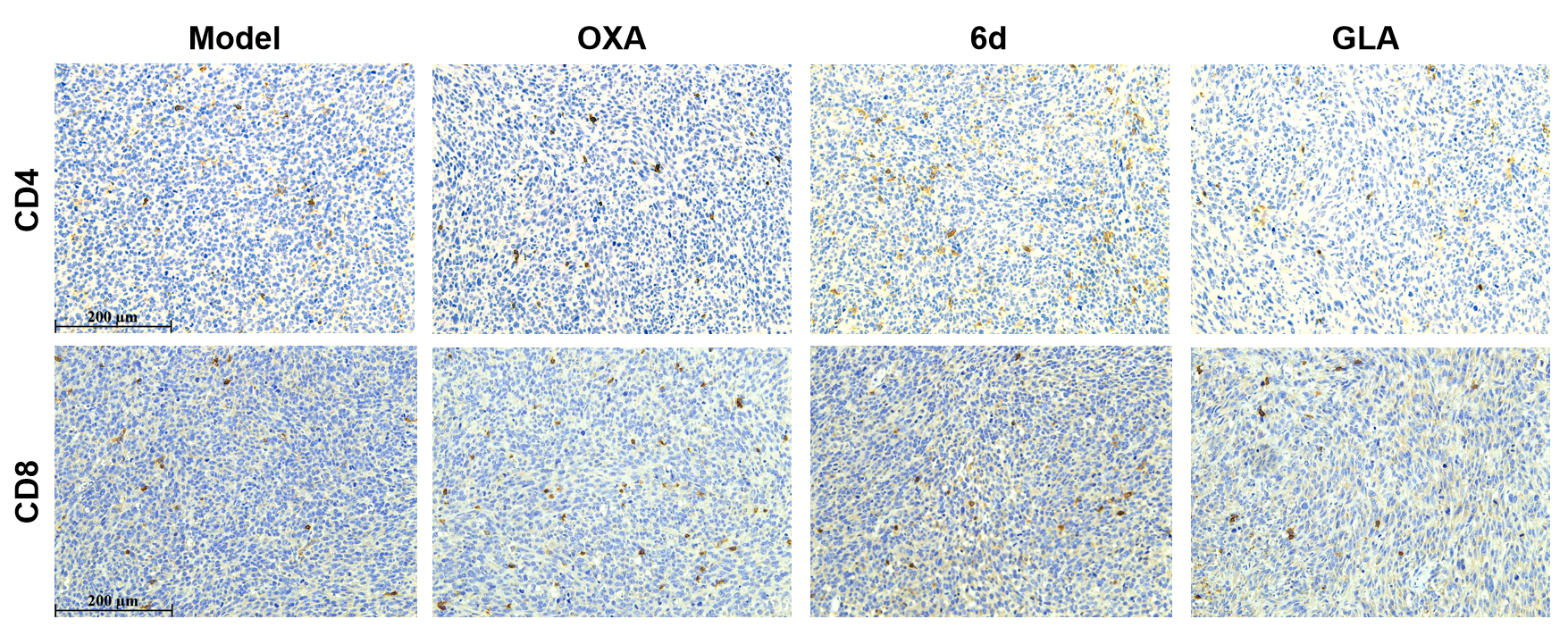


**Figure S57.** Immunohistochemistry analysis of CD4^+^ and CD8^+^ T cell infiltration in tumor tissue. (scale bar = 200 μm)


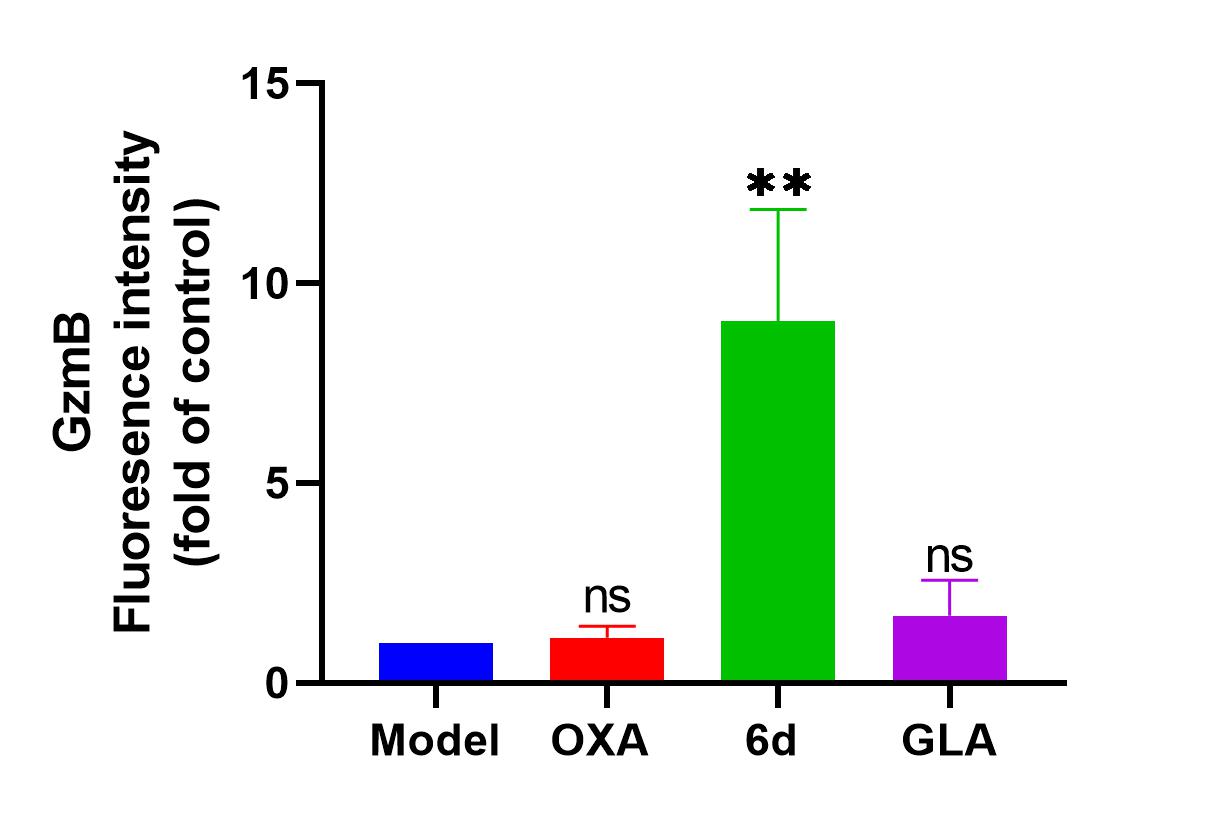


**Figure S58.** Quantification analysis of GzmB fluorescence intensity in Figure 6D. Data are expressed as the mean ± SD (n = 3); Student’s *t*-test, compared with Model group; ns > 0.05 and ***p* < 0.01.

**Author Contributions**

Zhaoran Wang and Meiyu Wang contributed equally to this work.
